# UDP-glucose 4, 6-dehydratase Activity Plays an Important Role in Maintaining Cell Wall Integrity and Virulence of *Candida albicans*

**DOI:** 10.1371/journal.ppat.1002384

**Published:** 2011-11-17

**Authors:** Manimala Sen, Bhavin Shah, Srabanti Rakshit, Vijender Singh, Bhavna Padmanabhan, Manikandan Ponnusamy, Koteppa Pari, Ram Vishwakarma, Dipankar Nandi, Parag P. Sadhale

**Affiliations:** 1 Department of Microbiology and Cell Biology, Indian Institute of Science, Bangalore, India; 2 Department of Biochemistry, Indian Institute of Science, Bangalore, India; 3 Piramal Life Sciences India Ltd., Mumbai, India; University of Toronto, Canada

## Abstract

*Candida albicans*, a human fungal pathogen, undergoes morphogenetic changes that are associated with virulence. We report here that *GAL102* in *C. albicans* encodes a homolog of dTDP-glucose 4,6-dehydratase, an enzyme that affects cell wall properties as well as virulence of many pathogenic bacteria. We found that *GAL102* deletion leads to greater sensitivity to antifungal drugs and cell wall destabilizing agents like Calcofluor white and Congo red. The mutant also formed biofilms consisting mainly of hyphal cells that show less turgor. The NMR analysis of cell wall mannans of *gal102* deletion strain revealed that a major constituent of mannan is missing and the phosphomannan component known to affect virulence is greatly reduced. We also observed that there was a substantial reduction in the expression of genes involved in biofilm formation but increase in the expression of genes encoding glycosylphosphatidylinositol-anchored proteins in the mutant. These, along with altered mannosylation of cell wall proteins together might be responsible for multiple phenotypes displayed by the mutant. Finally, the mutant was unable to grow in the presence of resident peritoneal macrophages and elicited a weak pro-inflammatory cytokine response *in vitro*. Similarly, this mutant elicited a poor serum pro-inflammatory cytokine response as judged by IFNγ and TNFα levels and showed reduced virulence in a mouse model of systemic candidiasis. Importantly, an Ala substitution for a conserved Lys residue in the active site motif YXXXK, that abrogates the enzyme activity also showed reduced virulence and increased filamentation similar to the *gal102* deletion strain. Since inactivating the enzyme encoded by *GAL102* makes the cells sensitive to antifungal drugs and reduces its virulence, it can serve as a potential drug target in combination therapies for *C. albicans* and related pathogens.

## Author Summary

*Candida albicans* is an opportunistic fungal pathogen which infects individuals with debilitated immune system either due to old age, diseases such as AIDS or immune suppressive treatments. The cell wall of *C. albicans,* like most pathogens, mediates interaction of the pathogen with the host and determines the outcome of the host-pathogen interaction. We discovered that inactivation of *GAL102* encoded UDP-glucose 4,6-dehydratase activity in *C. albicans* causes altered mannosylation of cell wall proteins and loss of cell wall integrity. The mutant cells thus show increased sensitivity to antifungal drugs that target cell wall. Importantly, these mutant cells show significantly lower virulence and reduced ability to elicit inflammatory cytokine responses from the host. Hence, inactivating the enzyme could significantly aid in controlling the infections by *C. albicans*. Since, the gene encoding the UDP glucose 4, 6-dehydratase is also present in many other fungal genomes, inhibitors of this activity could be useful in effective treatment of candidiasis and other fungal infections.

## Introduction

*Candida albicans* is a polymorphic fungus that causes infection of skin, nail, mucous membrane in healthy individuals and can lead to more severe infections of the vital organs in case of immune-compromised patients leading to death [1]. It is capable of growing in both yeast and hyphal forms and the yeast to hyphal transition has been reported to play a key role in virulence [2]. Environmental cues such as temperature, pH, serum, nutrient deprivation on solid media, etc. are known to trigger yeast to hyphal transition and *in vitro* studies have led to the identification of some of the key regulators such as *CPH1, EFG1, INT1, PRA1, RBF1, TUP1, UME6* etc. [3]–[5]. In general, mutants of some of these regulators show reduced hyphal morphology that correlates with reduced virulence, suggesting a direct correlation of the hyphal form with virulence of *C. albicans* [6]. Surprisingly, mutations in genes like *TUP1* and *NRG1* (global repressors of hyphal morphology) also show reduced virulence in spite of increased hyphal morphology [7]. This observation has raised questions about the validity of the direct correlation of hyphal morphology and virulence [8]. The morphological forms also differ in the cell wall composition [9]. Cell wall is the first cell organelle that comes in contact with the host and plays an important role in determining the outcome of the host pathogen interaction. Therefore, alterations in the cell wall composition and the associated transcriptional program, than the shape of the cell, per se, that might be crucial to virulence of *C. albicans*.

It has been reported that the cell wall architecture and virulence of microbial pathogens can be affected by presence of Galactose, a sugar that can act as a sole carbon source for many pathogens [10]. The effect of galactose on the biofilm development of *C. albicans* has been extensively studied. The rate of formation of biofilm is higher in the presence of galactose [11]. Further, galactose contributes to 3% of the dry weight of extra-cellular polymeric material of *C. albicans* biofilm [12]. Most organisms are able to metabolize galactose i.e. convert β-D-galactose to glucose 1-phosphate through four enzymes of the Leloir pathway which have been well characterized in *E. coli* and *Saccharomyces cerevisiae* [13]. The epimerase which catalyzes the third step in the Leloir pathway can clearly be an enzyme that may have a role beyond galactose metabolism, in that, the reversible reaction could be employed to generate galactose during growth on glucose as the sole carbon source. Indeed, various phenotypes are associated with mutations in this gene in different organisms [14], [15]. In *S. cerevisiae*, lack of Gal10p prevents growth on galactose as the sole carbon source [16] although, it has no reported effect on growth in the absence of galactose. Previous work from our laboratory has shown that *CaGAL10* (*orf19.3672*) encodes a true homolog of UDP-galactose-4-epimerase and can functionally complement the *S. cerevisiae gal10Δ* [17]. We have shown that the ultrastructure of the biofilm of Ca*gal10* mutant is distinctly different from that of the wild type [17]. In Arabidopsis, the impairment results in root-specific phenotypes, including increased root hair elongation, decreased root length, and root epidermal bulging etc. [18]. In humans, impairment of galactose epimerase causes one of two clinically distinct forms of galactosemia, an autosomal recessive epimerase-deficiency syndrome [19].

The *orf19.3674* which has been annotated as *CaGAL102* in the *Candida* Genome Database, encodes a protein very similar to the epimerase domain of the CaGal10p. We have previously shown that the full length CaGal10p as well as its epimerase domain alone complements the *S. cerevisiae gal10* deletion [17]. However, in light of a report [20] that *Cryptococcus neoformans* has two functional paralogs of *GAL10*, we asked the following questions: Does *CaGAL102* encode a functional galactose epimerase? If yes, what is the significance of two Gal10 paralogs in *C. albicans*? If it is not a functional epimerase what role does it play in *C. albicans*?

In the present study, we aimed to determine the role of this putative galactose epimerase in *C. albicans* using multiple experimental approaches. We found that the *GAL102* actually encodes a UDP-glucose 4,6-dehydratase activity and its loss affects the composition of cell wall mannans. The mutant cells lacking the activity also showed several defects in phenotypes associated with virulence of *C. albicans*. The mutant cells elicited a differential cytokine response from host immune system cells *in vitro* as well as *in vivo,* which reflected in attenuated virulence in mouse model of systemic candidiasis. These observations are in agreement with the reports that cell wall mannans contribute to host responses elicited by fungal pathogens [21], [22]. Our observations present a clear evidence for an enzyme activity that affects composition of cell wall mannans, cell morphology and contributes to virulence in *C. albicans*.

## Results

### A putative paralog of UDP-galactose 4- epimerase encoded by *GAL102* in *C. albicans* genome does not function as a galactose epimerase

**Figure 1:**
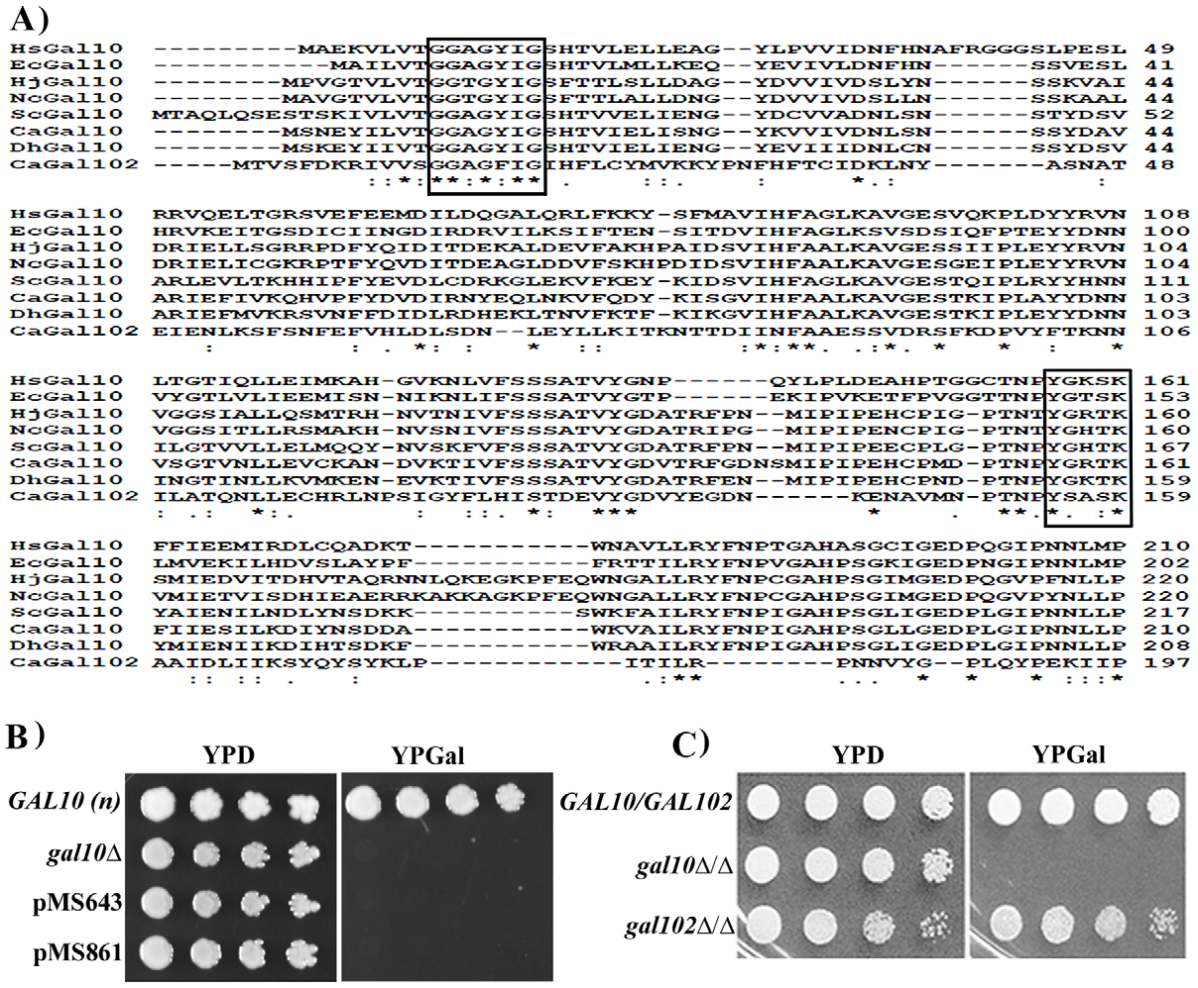
Putative Gal10 paralog does not encode functional Galactose epimerase. A) Multiple sequence alignment of Gal10 homologs from *E. coli* (Ec), *N. crassa* (Nc), *H. jecorina* (Hj), *S. cerevisiae* (Sc), *C. albicans* (Ca), *D. hansenii (Dh), H. sapiens* (Hs). The residues known to play a crucial role in catalysis or in NAD binding are shown in boxes. Identical residues are marked as * and similar residues are marked as ‘:’ B) *S. cerevisiae gal10* deletion strain transformed with pPS189 (empty vector) or, pMS643 (P*_TEF1_*-Gal102), pMS861 (P*_TEF1_*-Gal102*) were spotted on YPD and YPGal plates and incubated at 30°C. Plates were photographed next day. WT *S. cerevisiae* strain BY4741 (*GAL10*) was spotted as control. C) WT SC5314 (*GAL102/GAL102*), *Cagal10Δ/Δ* and *gal102Δ/Δ* cells were spotted on YPD and YPGal plates and checked for growth after 1 day.

*Orf19.3674/GAL102* in the Candida Genome Database has been annotated as a UDP-galactose 4-epimerase. Multiple sequence alignment of Gal102p with UDP-galactose 4-epimerase (Gal10p) homologs using the software ClustalW showed that the active site residues in the epimerase domain are highly conserved (Figure 1A). Incidentally, *CaGal10* shows 52% identity and ∼ 70% similarity to ScGal10p but Gal102p shows only 27% identity and 47% similarity. Most striking is the conservation of the YXXXK motif wherein the conserved tyrosine residue plays an important role during catalysis facilitating the transfer of a hydride from C4 of the sugar to C4 of NADH leading to a 4′-ketopyranose intermediate and NADH [23].

Most ORFs from *C. albicans* expressed in *S. cerevisiae* are able to complement loss of function mutants of corresponding homologs. To test whether *GAL102* encoded protein has UDP-galactose 4-epimerase activity we sub-cloned the ORF under the constitutive *TEF1* promoter on a multi-copy plasmid (pMS643). *C. albicans* uses an alternative genetic code with CUG codon encoding for Serine in place of Leucine of the universal genetic code. We replaced the only CUG codon with UCG to introduce Serine at the 314 position in the recombinant Gal102 ORF (pMS861) and then tested its ability to complement the *S. cerevisiae gal10* mutant strain PJB5. The *gal10* mutant is unable to grow in the presence of galactose as the sole carbon source. Transformants expressing the open reading frame grew on plates containing glucose as the sole carbon source but not in the presence of galactose (Figure 1B). We tested the presence of this protein in cells transformed with pMS861 using Western blot analysis to confirm that the failure to complement was not due to poor or lack of expression of the heterologous protein (Figure S1A).

We further disrupted the *GAL102* gene using a PCR based strategy as described earlier [24] and generated multiple isolate/mutants through independent transformations. We confirmed the deletion and the absence of the transcript by Southern and Northern analysis respectively (Figure S1B). We tested these mutants for various phenotypes and have presented here representative data from one of these isolates. We found that the null mutant *gal102Δ/Δ* had growth rate similar to that of the WT in normal rich media (YPD) at 30°C (data not shown). When spotted on YP glucose or YP galactose plates, the *gal102Δ/Δ* strain exhibited no growth defect (Figure 1C) while the *gal10Δ/Δ* mutant strain could not grow on galactose-containing medium [17]. This implied that the *GAL102* gene does not play any significant role in galactose metabolism in *C. albicans*. It has been reported that the two paralogs of *GAL10* in *C. neoformans* affect growth on galactose at different temperatures [20]. We saw no such difference in growth under suboptimal conditions such as high temperature or low galactose concentration in the absence of *GAL102* (data not shown).

### *GAL102* encodes a nucleotide sugar dehydratase

**Figure 2:**
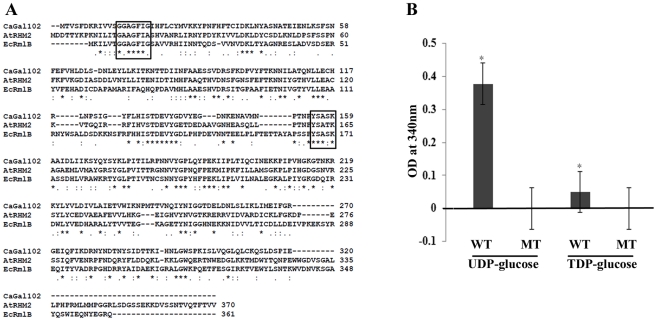
*GAL102* encodes a dTDP-glucose 4, 6-dehydratase. A) The sequence alignment of fungal homologs of dTDP-glucose 4, 6-dehydratase with Gal102 was generated using ClustalW. The homologs from *E. coli* and *A. thaliana* used for the multiple sequence alignments are shown. The conserved NAD binding motif and the active site YXXXK motif are boxed. B) Enzymatic activity of purified wildtype Gal102 (WT) and the catalytic mutant K159A (MT) was tested with UDP glucose, dTDP glucose as substrates. The activity was measured as NAD-NADH conversion by monitoring increase in A_340_. All the substrates and cofactor NAD were used at 3 mM concentration. The reactions were carried out at 37°C for 2 hr. Experiment was done in triplicate with 20 µg of recombinant protein expressed in *E. coli* and purified using Ni-NTA column per reaction. *p<0.05.

Since the deletion of *GAL102* showed no effect on galactose metabolism and associated phenotypes, we repeated the homology search through NCBI databases. We found that Gal102p showed 32% identity to dTDP-glucose 4,6-dehydratase (RmlB; Acc. No. AAB88398) of *E. coli* and 40% identity to the N-terminus of RHM2 (1–370 amino acids) of *A. thaliana.* The latter has been biochemically shown to have UDP-glucose 4,6-dehydratase activity. The dTDP/UDP-glucose 4,6-dehydratase enzyme is known to be involved in rhamnose biosynthesis in bacteria as well as in plants [25], [26]. Figure 2A shows the ClustalW alignment of Gal102p sequence with several other fungal homologs as well as those from *E. coli* and *A. thaliana*.

The dTDP/UDP-glucose 4,6-dehydratase enzyme is a member of the SDR (short chain dehydratase/reductase) family of proteins, which have a highly conserved YXXXK motif, crucial for the enzyme activity. In case of *A. thaliana,* the RHM2^K165A^ mutation completely abolishes UDP-glucose 4,6-dehydratase activity [26]. Lysine at position 159 in Gal102 lies within the putative catalytic motif, YXXXK. Therefore, we mutated it to alanine (AAA to GCA codon) as described in materials and methods section. We expressed the codon optimized Gal102p (as described above) and its mutant derivative Gal102p^K159A^ in *E. coli* and purified the recombinant proteins. We assayed for the dehydratase activity by measuring NAD- NADH conversion at 340 nm using either UDP glucose or dTDP glucose as substrates. After 2 hr the native Gal102p showed significant activity with UDP glucose as the substrate but only very low activity with dTDP glucose as the substrate. This activity could not be detected with the same amount of Gal102^K159A^ mutant protein (Figure 2B). This confirmed that Gal102p actually encodes a UDP-glucose dehydratase activity and that the lysine (Lys 159) in the conserved motif YXXXK, is essential for the activity of the enzyme.

### Deletion of *GAL102* leads to altered cell morphology and reduced cell wall integrity

**Figure 3:**
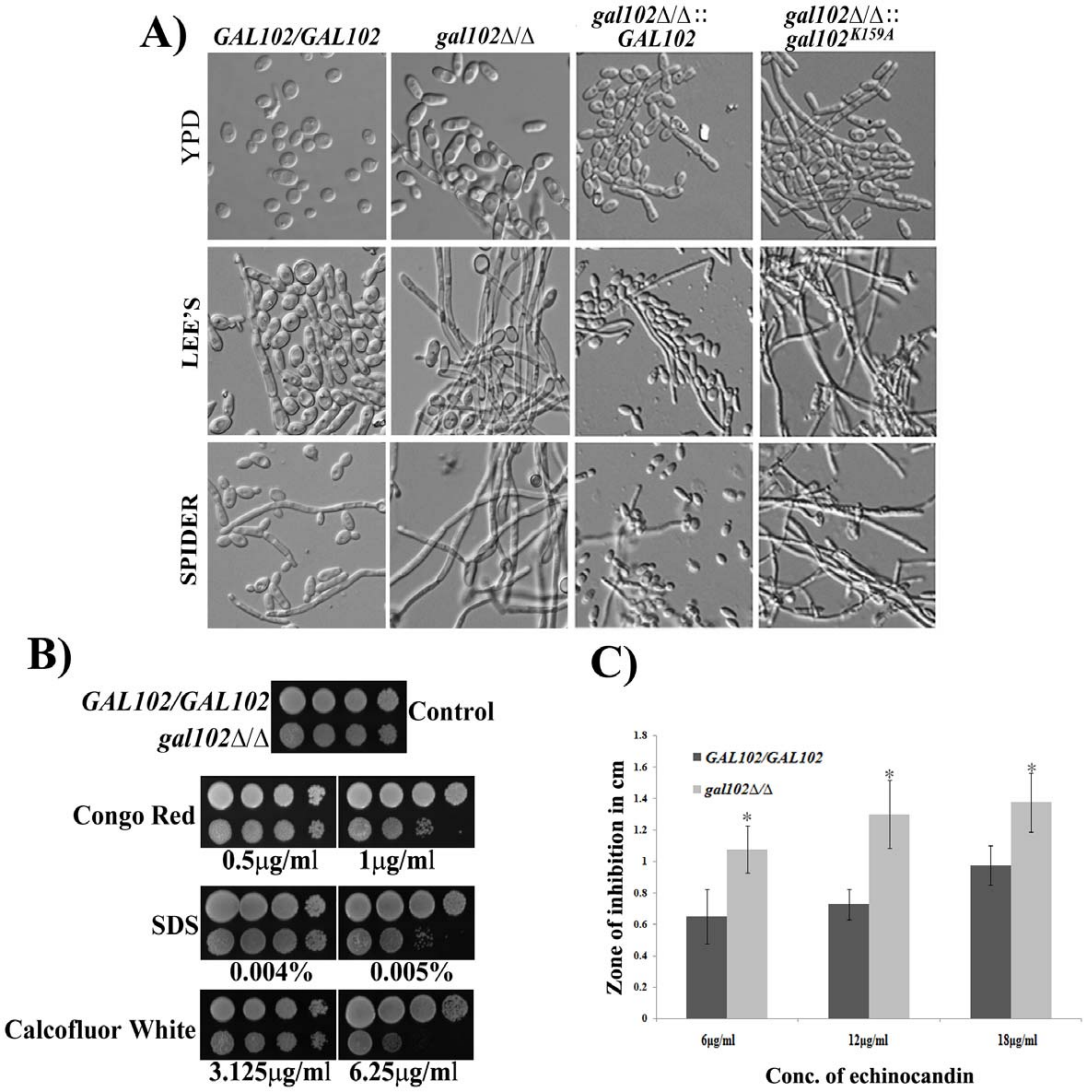
Deletion of *GAL102* affects cell morphology and cell wall integrity. A) Cells of *GAL102/GAL102, gal102Δ/Δ,* WT reintegrant and the K159A mutant reintegrant in *gal102Δ/Δ* strain were grown overnight at 30°C in YPD supplemented with uridine or Lee's or Spider medium. Cells were photographed using Olympus BX51 microscope with Nomarsky optics at 400X magnification. B) The *gal102Δ/Δ* and the *GAL102/GAL102* strain were incubated in YPD containing the indicated cell wall damaging agents at the indicated concentrations. C) The *gal102Δ/Δ* and the WT DAY286 strain were mixed with top agar and poured on top of YPD plates. Various concentration of Echinocandin (5–15 µg) was added to the wells and photographs were taken after 48 hr of incubation at 37°C. The results from 3 different plates were used to measure the diameter of zone of inhibition and plotted in the bar graph (lower panel). Grey bars represent WT while the black bars represent mutant. *p<0.05.

We observed that the cells of the *gal102Δ/Δ* strain were more elongated when grown in normal rich media (YPD) at 30°C as compared to the WT (SC5314) strain (Figure 3A). Thus, the deletion of *GAL102* has an effect on cell morphology which is also reflected in the wrinkled colony morphology (Figure S1C). When grown under hyphae inducing conditions, like growth in Lee's or Spider medium, the mutant showed much more enhanced filamentation as compared to the WT (SC5314).

To establish that the loss of enzyme activity of the Gal102p is responsible for the phenotypes associated with the deletion, we replaced the *gal102::HIS1* allele with either WT *GAL102* or *gal102^K159A^* allele. All these strains thus have *URA3* gene integrated at the *GAL102* locus whose expression from non-native locus has been reported to be associated with attenuation of virulence. The morphology of these marker matched reintegrant strains was then tested in hyphal inducing media like Lee's medium and Spider medium. It was observed that (Figure 3A) WT *GAL102* integrant at the gal102::HIS1 locus reduced the extent of filamentation close to that of the WT (SC5314) strain. In contrast, the catalytically inactive *gal102* ^K159A^ integrant at the same locus continued to show high filamentation phenotype. This shows that the morphological phenotypes associated with the deletion are indeed due to the lack of the glucose dehydratase activity associated with the protein.

We tested the sensitivity of *gal102Δ/Δ* strain to various cell wall damaging agents, e.g. SDS, Congo red and Calcofluor white as well as an antifungal antibiotic echinocandin. The mutant cells showed greater sensitivity to the cell wall damaging agents as compared to the WT (SC5314) strain (Figure 3B). The increased sensitivity to several cell wall damaging agents suggests that the cell wall composition of the mutant is altered in such a way as to affect the integrity of the cell wall. The antibiotic echinocandin specifically showed larger zones of inhibition as compared to the WT, signifying greater sensitivity of the mutant to this antibiotic supporting the observed cell wall integrity defect A bar graph consolidating three independent measurements of zone of inhibition at the indicated concentration underscores the significance of the observation (Figure 3C).

### Deletion of *GAL102* leads to altered biochemical composition of the cell wall mannans

**Figure 4:**
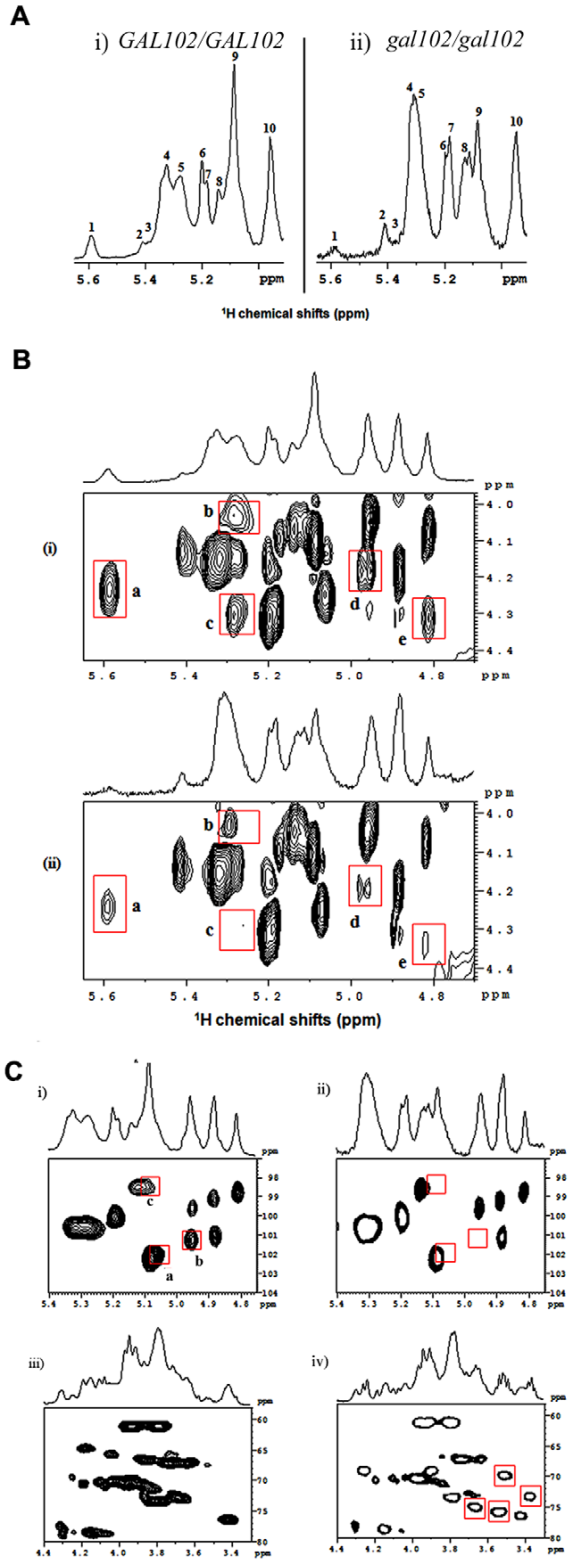
Deletion of the *GAL102* affects cell wall mannan composition. A) Anomeric region of ^1^H-NMR spectra of mannans from the WT (i) and *gal102Δ/Δ* (ii) of *C. albicans* recorded at 40°C temperature on a 500 MHz NMR spectrometer equipped with a triple resonance probe. Important changes that appear in the spectra for WT and the mutant samples are highlighted (and peaks are labeled with numbers). B) 2D ^1^H-^1^H TOCSY spectra of mannans from the WT (i) and the *gal102Δ/Δ* (ii) recorded on a 500 MHz NMR spectrometer equipped with a triple resonance probe. The mixing time for the TOCSY was fixed at 100 ms. The data was acquired at 40°C. Highlighted contours with red colors indicate the peaks of interest for comparison. C) Anomeric region of the 2D ^1^H-^13^C HSQC spectra of mannans from the WT (i) and *gal102Δ/Δ* (ii) recorded at 40°C on a 500 MHz NMR spectrometer equipped with a triple resonance probe. The panels (iii) and (iv) show 2D ^1^H-^13^C HSQC spectra of the WT and *gal102Δ/Δ* strain in the C-2, C-3 and C-6 proton/carbon region of the spectra. Highlighted contours with red box indicate the appearance of the new peaks in the spectra for mutant sample.

The above results indicated that the cell wall composition may be altered significantly in the *gal102Δ/Δ* mutant. Since the product of the Gal102 catalyzed reaction is a nucleotide sugar which might act as a sugar donor in cell wall protein glycosylation, we surmised, that the most likely target of the deletion was cell wall mannans which form the outermost layer of the *C. albicans* cell wall [9]. We analyzed the mannan composition of the mutant cell wall and compared it to that of the WT (SC5314). Earlier reports on NMR analysis of mannans from *C. albicans* cells have revealed differences among the yeast form and the hyphal form [9]. To ascribe the difference in mannan composition to the lack of Gal102 activity and not to the elongated cell morphology associated changes, we isolated mannans as described [9] from both the WT and the mutant cells grown at 25 °C at which the *gal102Δ/Δ* cells were much less elongated (Figure S1D). In our studies we performed ^1^H NMR and ^31^P NMR to study presence/absence of linkages among the mannans, and TOCSY, ROESY and HSQC to study the details of gross defects in specific bonding. The anomeric region of the 1D ^1^H-NMR spectra of mannans from WT [(i)], and *gal102Δ/Δ* [(ii)] are shown (Figure 4A).

A close observation of the corresponding proton NMR spectra revealed interesting differences in the structural assembly of mannan from the mutant as compared to WT. Both peaks #1 and #9 showed drastic reduction in the intensity. The former represents the loss or reduction of the entire β-1,2 link branched chain mannose units attached through phosphodiester linkage, and the latter, indicates the loss of considerable length of the α-1,6 backbone moiety. The internal change after the loss of α-1,6 backbone is reflected in the downfield shift of the peak 5 (merged into peak 4). The small, peak 3 which accounts for α-1,2 linked mannans in the acid-labile terminus is missing in the mutant. The mutant showed no change in the intensity of peak 10 suggesting the retention of the β-1,2 linked units (in the side-chain of acid resistant- terminus). The analysis of ^31^P NMR spectra (Figure S2) showed that the phosphodiester moiety in the side chain is absent in the mutant spectra implying that such linkage is either missing or substantially reduced.

A comparison of 2-dimensional ^1^H-^1^H-total correlation spectra (2D-TOCSY) of WT and the *gal102Δ/Δ* samples revealed significant difference in the spectra (Figure 4B). The reduced peak **a** indicated the loss of phosphodiester linkage with the β-1,2 mannan moiety. Peaks **b** and **c** come from the correlations between β 1 protons on the PO_4_-linked mannans (in the acid-labile region) to their respective C-6 and C-2 protons which are reduced in the spectra of the mutant samples. Additionally, the loss of intensity in peaks **d** and **e** indicates lack or substantial loss of part of the α-1,6 backbone mannan skeleton (revealing a reduction in the size of the α-1,6 backbone) which was further supported by the analysis of 2D ^1^H-^1^H ROESY (2-dimensional ^1^H-^1^H Rotating-frame Overhauser Effect Spectroscopy) spectra for both WT and the mutant (Figure S3).

**Figure 5:**
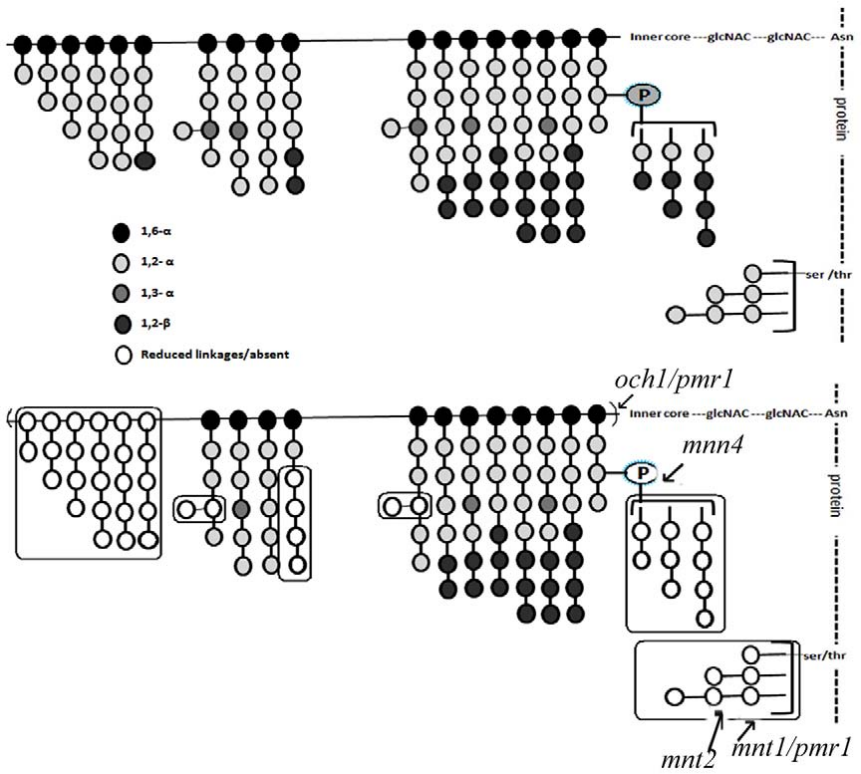
The mutant mannan composition is significantly different from that of the WT and is qualitatively distinct from the other known mannosylation mutants. Top panel represents the mannan profile of WT (SC5314) strain, based on Netea *et al.* [22] illustration. Bottom panel, represents the mannan profile of *gal102Δ/Δ*. Effects of other known mannosylation mutants on the mannan structure is also as represented by Netea *et al.* [22]. These are indicated to allow comparison with gal102 mutant. The various linkages between the mannosyl residues have been indicated by the shading of the residues. The legend for the link and the shade intensity is given at the bottom of the figure. The boxes highlight the most significant differences in the mannan profile between the WT and the mutant linkages that are either reduced or absent (denoted by empty circles) in the mutant.

A detailed analysis of a 2-dimensional ^1^H-^13^C-Heteronuclear Single Quantum Coherence (2D ^1^H-^13^C-HSQC) spectrum of WT and mutant samples revealed an interesting feature [Figure 4C (i) and (ii)]. Peak **a** in Figure 4C (i) corresponds to α-1 proton (δ_H_ 5.04 ppm; with its attached carbon, δ_C_ 101.73 ppm) of the 1,3,6-trisubstituted mannan moiety in the α-1,3 linkage of the acid-resistant terminus. The absence of this peak in the spectra for mutant reveals that the mutation leads to the loss of α-1,3 mannan linkage in the acid-resistant terminus. Peak **b** belongs to α/β proton of the terminal mannan β-1 unit (extreme left part of the α-1,6 backbone, see Figure 5) attached to α-1,2 mannose side-chain moiety missing in the mutant spectra, demonstrating the loss of entire α-1,2 side-chain in the acid-resistant terminal region of the mannan assembly. The significance of peak **c** has not been ascertained yet.

The proton up-field region of the HSQC spectra [Figure 4C (iii) and (iv)] indicates loss of a part of the α-1,6 backbone mannan assembly and highlighted peaks with red boxes indicate the appearance of new peaks in the spectra for mutant. These new peaks are attributed to C-2, C-3 and C-6 protons/carbons of the β-1,2 mannose side-chain units in the acid-resistant terminus. The sharp and narrower peaks appearing in the anomeric proton regions of the HSQC spectra demonstrate reduction in chain length and other branching side-chains in the mutant sample. Thus, these observations offer details of the structure of the mannan assembly in *C. albicans* which corroborate published reports [9] and highlight the changes occurring in it upon deletion of *GAL102*.

Based on the above 1D as well as 2D NMR analyses we propose the mutant mannan structure to be much different than the WT mannan as shown in Figure 5. The upper panel refers to the WT mannan structure as accepted in the literature [22]. Our study clearly shows that the length of the mutant mannan is substantially reduced and it lacks specific linkages in the branches including the phospho-mannans (Figure 5, lower panel). Significance of these alterations in relation to the other known mannosylation defective mutants is discussed below (see discussion section).

### Genome-wide differential gene expression in *gal102Δ/Δ* strain shows distinct reduction in expression of genes involved in cell wall morphogenesis and virulence

Since the mutant cells showed such distinct phenotypes as elongated cell morphology, and reduced cell wall integrity, we tested the genome wide expression profile of the mutant and compared it to that of the WT. Both WT and *gal102Δ/Δ* mutant were grown at 30°C in YPD till A_600_ = 1.2 and RNA was isolated from cells, labeled and used to probe the Agilent custom microarrays as described (see Materials and Methods).

In agreement with the filamentous morphology, the expression profile of the mutant cells showed several hyphal specific genes like *HWP1* and a transcriptional regulator *UME6* expressed at high levels while yeast phase specific genes like *YWP1* were down regulated. Differential expression of some of the genes like *HWP1*, *YWP1* and *GAL102* was validated using semi-quantitative RT PCR (data not shown). The levels of transcripts encoding several (characterized as well as putative) GPI-anchored proteins, e.g. *ALS7, PGA31, PGA37, RBR1* etc. were high in the mutant. While a negative regulator of hyphal morphology *NRG1* was also found to be up-regulated (Table 1); several genes known to play a role in biofilm formation, along with genes like *CHK1, CRK1, RRH2, SOD4* etc. that contribute to virulence of *C. albicans,* were significantly down-regulated (Tables S1, S2 in Text S1). While the gene expression profiling showed genes being expressed as expected from the observed phenotypes, it also provided hints that some of the other attributes associated with virulence such as biofilm formation and survival in disseminated candidiasis mouse model may be altered in the mutant. Apart from the GPI anchored protein coding genes and those involved in biofilm formation, several classes of genes also showed differential expression such as those encoding proteins intrinsic to membrane (GO:31224), α1,3 mannosyl transferase activity (GO:331), etc. Several genes associated with cell wall (GO: 5618) were also highly overexpressed.

**Table 1:** The list of representative genes differentially expressed in *C. albicans gal102Δ/Δ* as compared to the WT.

Classification	Percentage	Representative genes
Down regulated		
Biofilm associated	6	*ILV3, PHO89, MET15, HCA4, AHP1, CLN3, RHR2*
Virulence	12	*SOD4, CHK1, CRK1, RAS1, MNT2, RHR2*
Cell wall associated	11	*YWP1, GSC1, CHK1, DOT4, SKN1, SCH9, BMT7, orf19.1995*
Macrophage associated	8	*MAE1, ILV3, RHR2, orf19.4211, orf19.3131*
Misc.	63	iron proteins, metabolism involved genes, membrane proteins putative transcription factors chromatin associated genes
Up regulated		
Oligopeptide and ion transporters	10	*OPT4, OPT5,OPT7,SGE11,AAP1,IFC3, TPO3TNA12, HAK1*
Stress associated	11	*HSP31,IPF525,AHA1,IFA4,GPX1, OPT7, PGA28*
Cell wall and GPI anchored proteins	18	*RBT1, HWP1, SPR1, PGA31, PGA37, PGA55, RHD3, SSA2, SYG1, MDR1, ALS7, RBR1, HS10, FGR12, HYR4, CHT2*
Misc	61	*NRG1, LIP1, PHO114, CAS1, LYS143*, genes involved in metabolism, chromatin related genes

The genes are categorized based on available annotation in the Candida genome database.

### Macrophages inhibit the growth of *gal102Δ/Δ* strain *in vitro*

**Figure 6:**
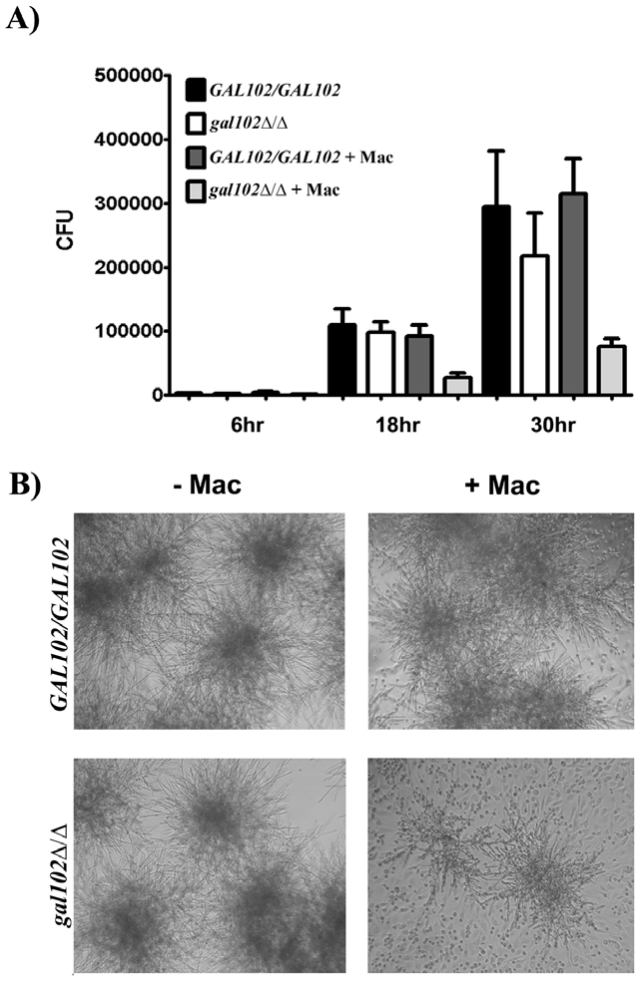
Macrophages suppress the growth of *gal102Δ/Δ in vitro.* A) Live *C. albicans* (WT or *gal102Δ/Δ*) were cultured in the absence or presence of adherent resident peritoneal macrophages for different time periods. Following incubation, *C. albicans* were washed and plated onto YPD agar plates. Data are pooled from at least two separate experiments with a total of three experiments (mean ± SD), *p < 0.05, *t* test. B) Representative micrographs depicting growth of WT and *gal102Δ/Δ* strains of *Candida* in the absence (left panel) or presence (right panel) of resident peritoneal macrophages for 18 hr is shown.

Since the cell wall mannans constitute the outermost layer of the *C. albicans* cell wall we expected that the altered mannan composition might alter interactions with the host cells. The outcome of the interaction between phagocytic cells such as macrophages and pathogens such as *C. albicans* can be studied using cell viability assays *in vitro* [27]. To study the response of macrophages to *gal102Δ/Δ* strain, both WT and *gal102Δ/Δ* mutant cells were incubated with mouse resident peritoneal macrophages and their growth was monitored at indicated time points. At 6 hr, there was no difference in the growth as measured by the CFU numbers, in the presence or absence of macrophages. However, by 18 hr there was a slight reduction in the growth of *gal102Δ/Δ* strain in the presence of macrophages and by 30 hr there was a substantial reduction in the proliferation relative to cultures without macrophages. The WT strain, on the other hand, proliferated to the same extent in the presence or absence of macrophages (Figure 6A). This inhibition of growth of the *gal102Δ/Δ* strain in the presence of macrophages is illustrated in micrographs (Figure 6B). These observations demonstrate that resident macrophages suppressed the proliferation of the *gal102Δ/Δ* strain but not WT *C. albicans*.

To determine cytokine response mediated by WT and *gal102Δ/Δ in vitro*, live and heat-killed *C. albicans* strains were incubated with resident peritoneal macrophages for varying time points. Incubation of macrophages with live WT *Candida* induced 3–4 fold higher levels of TNFα and IFNγ compared to the *gal102Δ/Δ* strain and the amounts steadily increased from 6 to 30 hr (Figure S4A and B; left panel). In case of heat-killed *Candida*, the levels were reduced but differences in the cytokine amounts between WT and *gal102Δ/Δ* were still significant (Figure S4A and B; right panel) and reflected the same pattern as that induced by the live cells. Interestingly, the *gal102Δ/Δ* strain induced higher amounts of IL4 production compared to that induced by WT (Figure S4C). These observations demonstrate that peritoneal macrophages elicit distinct cytokine patterns in response to WT and the *gal102Δ/Δ* strains.

### Deletion of *GAL102* leads to changes in biofilm characteristics

**Figure 7:**
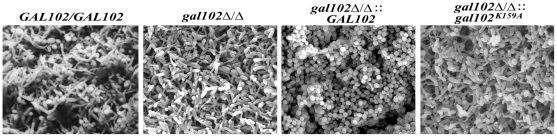
Biofilm structure and diffusion properties are seriously compromised in *C. albicans* lacking *GAL102*. The biofilms of WT (SC5314), *gal102Δ/Δ,* and WT and the K159A mutant reintegrant in *gal102Δ/Δ* strain were allowed to be developed as described in the Materials and Methods section. The biofilms were observed under a scanning electron microscope after appropriate treatment. Representative pictures at 1500X magnification are shown.

The problem in treating *Candida* infection becomes more complicated when it adheres to host surface or to the inner walls of catheters in patients and forms a biofilm, which is resistant to a variety of antifungal agents [28]. The biofilm formed by the WT strain is made up of both the yeast and the hyphal forms and the mutants that are unable to show yeast to hyphal transition, do not form effective biofilms [28]. The ability of *gal102Δ/Δ*, which shows predominantly elongated cell morphology, to form biofilm was determined using protocols described previously [17]. The WT SC5314 cells formed biofilm which consisted of a mixture of yeast form and filamentous cells which appear healthy and exhibit turgor (Figure 7). Although the ability of the *gal102Δ/Δ* to form biofilm was not affected, the ultrastructure observed under a scanning electron microscope was quite different (Figure 7). In case of the *gal102Δ/Δ* mutant strain, the biofilm consisted mainly of elongated cells that showed relatively less turgor, which could reflect weakened structure of the cell wall. The WT reintegrant biofilm showed presence of much higher proportion of yeast form cells as observed in case of biofilms formed by DAY286 WT strain (data not shown). The mutant reintegrant showed biofilm comparable to that of the *gal102Δ/Δ* strain.

### The *gal102Δ/Δ* strain is less virulent and causes much less organ damage than the WT in a mouse model of systemic candidiasis

**Figure 8:**
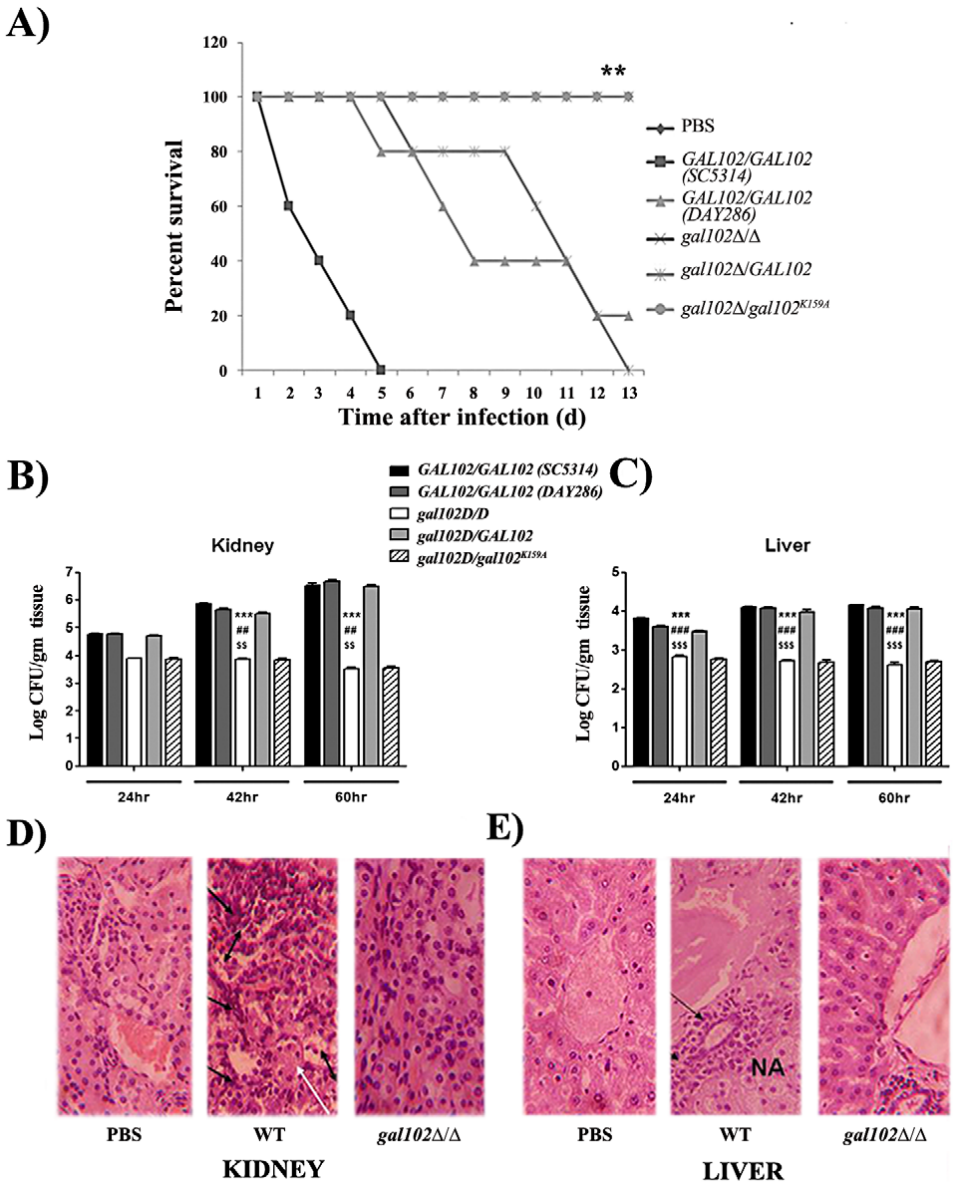
Infection of mice with *gal102Δ/Δ* leads to enhanced survival and reduced fungal tissue burden. Mice were infected intravenously with 5×10^6^ cells of WT (SC5314 and DAY286), *gal102Δ/Δ,* and the *GAL102* or the *gal102^K159A^* mutant reintegrants in *gal102Δ/Δ* strain. Mice were sacrificed at 24, 42 and 60 hr after infection and disease progression was assessed. A) The survival curve and statistical differences between strains were analyzed by Prism software using the Log-rank (Mantel-Cox) test. Data from one experiment using five mice is shown and is representative of three identical, separate experiments. **p < 0.01, *t* test. (B and C) Subgroups of three to five mice were sacrificed at 24, 42 and 60 hr after infection Quantitative fungal burden in kidney and liver was measured by serial dilution and expressed as log CFU/gram tissue. Data are represented as mean ± standard errors of the means from three separate experiments with three to five mice per strain. ***p<0.0001, ^##^ *p*<0.01 and ^$$^ *p*<0.01 compared with SC5314, DAY286 and WTRI (for (*gal102Δ/Δ*) injected mice) in kidney at 42 and 60 hr. ***p<0.0001, ^###^ *p*<0.0001 and ^$$$^p<0.0001 compared with SC5314, DAY286 and WTRI (for (*gal102Δ/Δ*) injected mice) in liver at 24, 42 and 60 hr. (D and E) Kidney and liver tissue sections were dissected from mice sacrificed at 60 hr, stained with hematoxylin & eosin and histological changes were observed. Sections from PBS treated mice were used as control. A representative histopathological examination is shown (magnification 400X for both panels). Black arrows indicate inflammatory cells, NA denotes necrotic areas in liver and white arrows indicate tubular casts and double headed arrows indicate sloughing of the tubular epithelial cells in the kidney.

To test the effect of alteration in the cell wall mannan composition on virulence of the *gal102Δ/Δ,* we performed intravenous injection into the lateral tail vein of female BALB/c mice with the WT and the *gal102Δ/Δ* strain. To ensure that high filamentation of *gal102Δ/Δ* strain does not lead to clogging of the tail vein during injection, or lead to inaccurate estimation of cell numbers we grew both WT and *gal102Δ/Δ* strain at 25°C. As mentioned earlier, at this temperature *gal102Δ/Δ* strain exhibited much less elongated cell morphology (Figure S1D). The resulting survival curves demonstrate that virulence of *gal102Δ/Δ* strain is greatly reduced. At 5×10^6^ cells of WT *C. albicans* per animal, 50% of the mice died within 3–4 days and the rest by 6 days (Figure 8A). However, ∼80% or more of mice injected with the *gal102Δ/Δ* strain survived the entire 30 day duration of observation (data not shown). As mentioned above, the *gal102Δ/Δ* and the WT and the *gal102^K159A^* reintegrant strains, all have *URA3* gene integrated at the *gal102* locus. We tested the virulence of these genotype matched strains in a mouse model of systemic candidiasis test as before. The clinical isolate SC5314 which is routinely used as the standard for virulence studies showed that it kills the host with faster kinetics than the DAY286, a strain with *URA3* reintegrated at the *ARG4* locus. This is consistent with earlier reports that the DAY286 strain is ∼70% as virulent as compared to SC5314 [29]. As seen in the survival curve (Figure 8A), the WT reintegrant showed virulence levels comparable to the WT DAY286 strain while the mutant *gal102^K159A^* reintegrant strain, as expected, showed low virulence, comparable to the *gal102Δ/Δ* parent strain. These results clearly show that the deletion/inactivation of *GAL102,* is responsible for the alteration in morphology as well as compromised virulence and not the expression of *URA3* gene from a non-native locus [30].

To evaluate whether reduced virulence of *gal102Δ/Δ* strain was due to its reduced multiplication *in vivo*, in a parallel set of experiment mice infected with both WT and mutant were sacrificed and fungal colonization of both, kidney and liver, the major target organs in systemic candidiasis, was determined. Increased fungal burden was observed in WT-infected mice from 24 hr to 60 hr after initiation of infection. On the other hand, CFUs recovered from the kidney and liver of mice infected with the *gal102Δ/Δ* strain were significantly lower than the WT and steadily decreased with time (Figure 8B and C). Both the reintegrants showed expected phenotypes similar to the WT and *gal102Δ/Δ* strains respectively. The *Candida* cells isolated from kidneys of mice infected with the *gal102Δ/Δ* strain clearly showed the wrinkled colony morphology characteristic of the mutant strain (data not shown) confirming the presence of the mutant in the infected hosts. The other markers of kidney and liver damage namely elevated levels of serum urea [31] and of the enzyme alanine aminotransferase (ALT) [32] were also monitored in case of mice infected with either the WT (SC5314) or the *gal102Δ/Δ* mutant and corroborated the damage to these organs by the WT but not the *gal102Δ/Δ* strain (Figure S5A and B). The liver sections from the control (PBS-treated) mice revealed normal hepatic architecture whereas mice infected with WT *C. albicans* showed necrotic patches. Moreover, the kidney sections of WT infected mice showed damage probably due to an excessive inflammatory response. In WT kidney, massive sloughing of the tubular epithelial cells into tubular lumen and deposition of tubular casts was observed compared to PBS and *gal102Δ/Δ*. Intra-tubular infiltration of inflammatory cells was also high in WT compared to PBS and *gal102Δ/Δ*. In WT infected liver, significant infiltration of the inflammatory cells around the peri-arterial space was observed compared to the PBS and *gal102Δ/Δ*. The hepatocytes around the peri-arterial space also exhibited nuclear pycnosis and hydrophobic degeneration in WT compared to PBS and *gal102Δ/Δ* group. However, liver of mice infected with the mutant strain did not display any necrotic regions or infiltration in the kidney by cells involved in inflammatory response and was comparable to PBS treated mice (Figure 8D and E; Figure S6A and B). These observations demonstrate that WT infection causes organ damage which is not seen upon infection with *gal102Δ/Δ.*

### The reduced virulence of *gal102Δ/Δ* is associated with its inability to elicit a strong pro-inflammatory response

**Figure 9:**
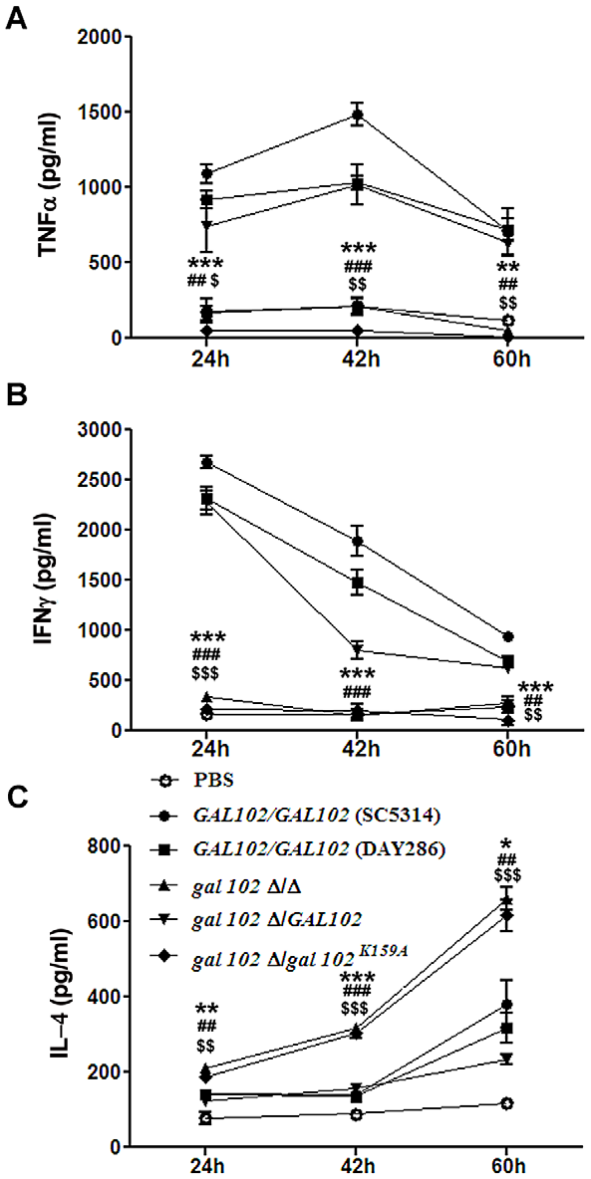
Reduced virulence is associated with the inability of *gal102Δ/Δ* to elicit pro-inflammatory cytokine response in the host. (A, B and C) Mice were infected intravenously with WT (SC5314 and DAY286), *gal102Δ/Δ,* and the *GAL102* or the *gal102^K159A^* mutant reintegrants in *gal102Δ/Δ* strains and were sacrificed at different time points for measurement of serum cytokine amounts. TNFα (A), IFNγ(B) and IL-4 (C) amounts were determined by ELISA. Sera from PBS treated mice were taken as control. Data are represented as means ± standard errors of the means from three separate experiments with sera of five mice. ***p<0.001, **p<0.01, *p<0.05; ^###^ *p*<0.001, ^##^ *p*<0.01,^ #^ *p*<0.05 and ^$$$^ *p*<0.001, ^$$^ *p*<0.01, ^$^ *p*<0.05 compared with SC5314, DAY286 and WTRI (for (*gal102Δ/Δ*) treated).

The observation that the *gal102Δ/Δ* cells displayed reduced virulence led us to study their ability to elicit cytokine production. Mice were injected (as above) with equal number of cells of WT (SC5314 and DAY286), *gal102Δ/Δ,* and the *GAL102* or the *gal102^K159A^* mutant reintegrants in *gal102Δ/Δ* strain and sacrificed at various intervals. The amounts of different cytokines in serum were monitored by ELISA. We observed that the amounts of the pro-inflammatory cytokines, TNFα and IFNγ were significantly higher in mice infected with WT than those infected with *gal102Δ/Δ* strain (Figure 9A and B). It is interesting that IFNγ amounts were initially high and decreased later; however TNFα was induced early and remained high for the WT. At the later time points, the lowered IFNγ amounts correlated with increase in the anti-inflammatory cytokine IL-4. In striking contrast, infection with the *gal102Δ/Δ* strain was characterized by a rapid and increased production of IL-4 (Figure 9C). These experiments demonstrate that infection with the *gal102Δ/Δ* strain resulted in an altered cytokine response in the host. The WT cells elicited high levels of pro-inflammatory cytokines, such as TNFα and IFNγ, whereas these cells elicited much higher levels of anti-inflammatory cytokine response, e.g. IL-4 (Figure 9A–C). This was also true with the WT and the mutant reintegrant indicating that the activity of the enzyme was restored in the WT reintegrant and lack of which in the mutant reintegrant is responsible for the observed opposing effects on cytokine production.

## Discussion

We initiated this work following the observation that *C. albicans* genome carries two genes encoding putative paralogs of UDP-galactose epimerase enzyme. We had earlier shown that Ca*GAL10* gene encodes a UDP-galactose epimerase [17]. During the course of this study we found that although the gene in *C. albicans* was annotated as UDP-galactose epimerase (Ca*GAL102*), the gene does not appear to encode a functional galactose epimerase but an enzyme with UDP-glucose 4,6-dehydratase activity. The *RHM2* gene of *A. thaliana*, is the only other eukaryotic homolog which has been demonstrated to have this activity [26]. Prokaryotic homologs of this enzyme typically use dTDP-glucose as the substrate [25]. The dTDP/UDP-glucose 4,6-dehydratase enzyme homologs control cell wall composition in bacteria and plants and in both cases the enzyme functions in the rhamnose (and/or related sugar) biosynthetic pathway. Rhamnose, is a component of the cell wall of these organisms and inhibition of its biosynthesis leads to cell wall integrity defects. In pathogenic bacteria the defect in cell wall integrity leads to loss of virulence. In *A. thaliana* mutants, it is known to lead to root morphology defects [33]. However, in the genomes of very few fungal species has the homolog of this enzyme been reported and is clearly absent in the filamentous fungi, e.g. *Aspergillus*. The homolog in some of the above fungal species has been annotated as a putative dTDP-glucose 4,6-dehydratase without any experimental evidence and in *C. albicans* it was annotated incorrectly as galactose epimerase as evidenced from our present study. Since the presence of rhamnose has not been reported in any of these fungal species, and the homologs of downstream enzymes in the rhamnose biosynthetic pathway are absent in their genomes, we addressed the following questions with *C. albicans*, an important fungal pathogen as the representative species: a) what is the significance of presence of this gene in *C. albicans*? b) Does it affect cell wall biogenesis and c) is it relevant to its virulence?

### The inactivation of UDP-glucose dehydratase drastically affects composition of cell wall mannan

Cell wall is the major organelle of a pathogen that comes in contact with the host cells and plays an important role in the outcome of the host-pathogen interaction. Many studies have revealed that the major components of the fungal cell wall such as mannans, glycans and proteins all contribute to morphology as well as the host response elicited by the pathogen. The mutants of *C. albicans* which lack specific components of mannan, the outermost layer of the cell wall, show reduced virulence indicating subtle contributions of individual components to virulence [34]. The two morphological forms of *C. albicans*, the yeast and the hyphae, are known to differ in the proteins associated with the cell wall, as well as the mannans which greatly contribute to the host response [34]. Several mutants have been studied which show alterations in various features of the cell wall. It has been reported that the *och1* mutant is defective in outer, branched *N*-linked glycans [35]. The *mnt1 mnt2* mutant lacks all the 4 terminal *O*-linked α1,2-mannosyl residues, but has normal *N*-linked mannan [36]. The *pmr1* mutant has defects in both *N*- and *O*-linked mannosylation [37] and the *mnn4* mutant lacks phosphomannan [38]. While the *gal102* mutant lacked the phosphomannan like the *mnn4* mutant, it showed substantially reduced levels of the O-linked mannosyl residues and had less drastic effect on the N-linked mannan as compared to the *pmr1* mutant. Thus, the *gal102* mutant displayed a mannan profile that is distinct from any of these above mentioned known mannosylation defective mutants (Figure 5).

### Alteration in cell wall composition is associated with differential expression of several cell wall protein coding genes

Cell wall has been considered a dynamic rather than a static assemblage of macromolecules and recent studies have indicated that the *Candida* cells exhibit altered mannoprotein composition in response to environmental stimuli [39]. Our genome-wide differential gene expression studies of the mutant showed that a large number of hypha-specific genes were upregulated while yeast form-specific Ywp1 protein was clearly downregulated which is commensurate with the morphology of the cells and increased tendency of the mutant cells to show hyper-filamentation. Among the classes of downregulated genes, the most significant were membrane protein encoding genes as well as those involved in biofilm formation. Interestingly, among the over expressed genes under the categories like cell wall (*p* = 4×10^−8^) hyphal cell wall, carbohydrate metabolism etc. were much higher and most significant. Some of the genes encoding cell-surface proteins such as *ALS2*, *ALS9* which are known to be expressed during infection were also found to be downregulated while *ALS6/ALS7* appear to be upregulated. It has been suggested that in response to cell wall integrity defects some compensatory mechanisms cause increase in expression of several cell wall proteins [40]. In the *gal102Δ/Δ* mutant the genes coding for GPI-anchored cell wall proteins showed increased expression levels. It has been known from work based on *S. cerevisiae* system as well, that expression of GPI-anchored proteins is drastically altered when cell wall synthesis is defective [40]. It has been suggested through many studies that cell wall is an important organelle in fungi, whose complex structure is carefully managed by the cell and altered according to various environmental and internal cues. The cell wall composition in turn affects survival of the fungal cell in the host as it elicits specific response from host immune system.

### Altered cell wall composition of the *gal102* mutant induces altered host cytokine response which correlates with reduced virulence

Mammalian hosts can respond to the presence of a pathogen by producing a variety of inflammatory molecules which help curtail the infection. These can be categorized into two broad groups: those involved in pro-inflammatory responses (e.g. TNFα & IFNγ) and anti-inflammatory responses (e.g. IL4 & IL13). The balance of pro- and anti-inflammatory response is thought to be important for the outcome of fungal infections. TNFα, a pro-inflammatory cytokine is a key mediator in protecting against disseminated candidiasis, as demonstrated by the fact that the lack of TNFα worsens the course of disseminated candidiasis [41]. Furthermore, IFNγ another pro-inflammatory cytokine is thought to play a dual role during candidiasis. On one hand, it is required for host resistance as shown in studies using *Ifnγ* ^−/−^ mice [42]; on the other hand, excess IFNγ production during infection leads to host damage leading in turn, to increased susceptibility to *C. albicans* [43]. Most importantly, dendritic cells pulsed (*ex vivo*) with yeast cells, when transferred into mice, protect them from challenge with *C. albicans* [44]. This protection was not observed with dendritic cells pulsed with hyphal cells. These studies clearly underscore the importance of pro-inflammatory cytokines in the host resistance to *C. albicans* infection. There are conflicting reports on the role of IL-4 during candidiasis. One report involving intra-peritoneal challenge with *C. albicans* blastoconidia showed that IL-4 was not involved in resistance [42]. However, the unrestrained IFNγ response during early stages of *C. albicans* infection in *Il4* ^−/−^ mice, leads to resistance and only at later stages are the *Il4* ^−/−^ mice susceptible due to failure in mounting a strong Th1 response. In fact, treatment with exogenous IL4 boosts immunity against *C. albicans* [45].

The *gal102* mutant showed virulence defect in disseminated mouse model system and the same reflected in its inability to grow in the presence of macrophages *in vitro*. *In vitro* as well as *in vivo* studies showed that the mutant was unable to induce pro-inflammatory cytokine production in contrast to that observed with WT *C. albicans* infection. This was indeed surprising since reduced pro-inflammatory cytokine response could be expected to increase survival of the pathogen in the host. It is well known that *C. albicans* yeast and the hyphal forms elicit different responses from the host immune system with the yeast form inducing pro-inflammatory cytokines, and the hyphal form inducing anti-inflammatory cytokines [34], [44]. The high pro-inflammatory cytokine response is required for host defence; however excessive inflammatory responses cause damage to the host [46], [47]. The better survival and growth of the WT pathogen in the host leads to increased production of pro-inflammatory cytokines, resulting in excessive tissue damage. On the other hand, it is possible that the defective cell wall of *gal102* mutant renders these cells susceptible to killing by host cells (Figure 6) and at the same time lower levels of pro-inflammatory cytokines induced by the mutant cause reduced tissue damage (Figure 8D and E) resulting in increased host survival (Figure 8A). The *gal102* mutant induces increased production of IL4, an anti-inflammatory cytokine which is responsible for the increased killing and reduced growth of mutant cells (Figure 9). These results clearly underscore the important roles of pro- and anti-inflammatory cytokines, which have major implications in terms of immunity against fungal pathogens.

In *C. albicans* marker gene expression has been shown to be drastically affected depending on the locus of integration. Among the most favorite of the marker genes *viz. URA3* gene in *C. albicans* has been shown to affect virulence attributes depending on its chromosomal location [30]. Re-integration of the WT ORF and the catalytically inactive mutant ORF in the native locus allowed us to compare the phenotypes of the deletion mutant irrespective of the *URA3* expression levels since all the three strains, i.e. *gal102Δ/Δ and* the WT or mutant reintegrant carried the *URA3* marker at the *GAL102* locus. The WT reintegrant showed rescue of most phenotypes tested e.g. filamentous morphology, virulence attributes and the cytokine response elicited by the mutant, while the catalytically inactive mutant with only a single lysine to alanine change in the YXXXK motif showed phenotypes akin to the parent *gal102Δ/Δ* mutant.

### UDP glucose 4,6-dehydratase activity although present in non-pathogenic *S. pombe* affects virulence of *C. albicans*

In summary, our characterization of *gal102,* a putative paralog of UDP-galactose 4-epimerase in *C. albicans*, has led to the conclusion that it is not a galactose epimerase. Although sequence analysis reveals that it is a member of the Short chain dehydratase reductase (SDR) family, whose members have a conserved, glycine rich, NAD/NADP-binding motif as well as a catalytic YXXXK motif [48]. The present study for the first time has presented evidence that a fungal homolog actually possesses the UDP-glucose 4,6-dehydratase activity. Yet, the homologs of enzymes participating in the subsequent steps involved in rhamnose biosynthesis have not been identified and the product rhamnose has not been detected in *C. albicans* or other related fungi. This raises a distinct possibility that Gal102p may not be involved in the rhamnose biosynthetic pathway in these fungi. It is known that nucleotide sugar moieties are used as sugar donors in glycosylation reactions. Since we have observed significant differences in the mannosylation profile of the mutant, it is possible that this enzyme functions upstream of a variety of glycosylation reactions involved in cell wall protein mannosylation in *C. albicans* and many of the related fungal pathogens. We have shown here that in *C. albicans,* the absence of the activity affects cell wall mannans, cell wall integrity and morphology with consequences on survival in the host and virulence. The product of this enzyme activity likely acts as a donor of sugar residues in the synthesis of mannans and might affect GPI anchoring of proteins which are known to affect cell wall integrity and morphology of cells [49], [50]. We speculate that loss of cell wall integrity might trigger over-expression of several GPI proteins as a salvage response. The mutant with reduced cell wall integrity is also more susceptible to extracellular killing in the host as compared to WT cells. At the same time altered mannan composition elicits weaker pro-inflammatory cytokine response reducing host tissue damage and further allowing host to survive longer to be able to overcome the infecting mutant cells. The WT cells, on the other hand, can cause greater mortality due to the increased tissue damage induced by high levels of pro-inflammatory cytokines. Hence, in spite of the elongated cell morphology, the *gal102* mutant remains avirulent. Perhaps, it's not the cell morphology, but the composition of the cell wall that plays a key role in virulence.

Interestingly, Ca*GAL102* was not included among the 674 target genes studied by Noble et al. [51] as those likely to be involved in virulence of *C. albicans.* It turns out that this study had excluded genes that had sequence homologs in either *S. cerevisiae* or *S. pombe*, two of the well-studied non-pathogenic yeasts. CaGal102p is highly homologous to the protein encoded by the *S. pombe* gene SPBPB2B2.11 and recent phylogenetic analysis of GAL cluster has indicated that *S. pombe* might have acquired the entire *GAL* cluster including Ca*GAL102* homolog from a *Candida* species [52]. Hence it is not surprising that the above interesting study [51] missed Ca*GAL102* as one of the genes affecting virulence of *C. albicans*. It is also well known that genes whose products contribute to virulence can have homologs in related non-pathogenic microorganisms and, thus, are not necessarily unique to the genomes of pathogens. Assigning the direct role of the biochemical activity of CaGal102p in virulence of the fungal pathogens will require further in-depth studies through biochemical, genetic and molecular analyses.

## Materials and Methods

### Animal ethics statement

All experiments involving mice were conducted in compliance of the Ministry of Environment and Forests Act on breeding of and experiments on animals (control and supervision) rules, 1998. Mice were housed in the Central animal facility of IISc and studies were performed as per the guidelines laid out by the institutional animal ethics committee, IISc (permit no. CAF/Ethics/174/2009). The Central animal facility is accredited to the Ministry of Environment and Forests, Government of India. The guidelines followed are approved in consultation with the Committee for the purpose and control and supervision of experiments on animals (CPCSEA) can be seen in detail in the following document: http://envfor.nic.in/divisions/awd/cpcsea_laboratory.pdf.

### Strains and growth medium

The *C. albicans* strains used in this study are listed in Table 2. Standard growth media were used for *C. albicans*. Details of composition are provided in supplementary methods section. Prior to challenge in the animal model, a suspension was made by transferring to YPD broth and incubating at 30°C with shaking for a period of 16–24 hr. The *S. cerevisiae* strain was transformed with the plasmid constructs listed in Table 3 to generate the appropriate strains assayed for various phenotypes in this study.

**Table 2:** List of *C. albicans* and *S. cerevisiae* strains.

Strain	Genotype	Reference
*C. albicans*		
SC5314	Clinical isolate	[58]
BWP17	*ura3::λimm434/ura3::λimm434; his1::hisG/his1::hisG; arg4::hisG/arg4::hisG*	[59]
DAY286	*BWP17, ura3*::*imm434*/*ura3*::*imm434, pARG4*::*URA3*::*arg4*::*hisG*/*arg4*::*hisG his1*::*hisG*/*his1*::*hisG*	
CAS8	BWP17, *gal10::URA3/gal10::gal10, MET3::pFA-ARG4*	[16]
CAS12	BWP17, *gal102::HIS1/gal102::URA3, MET3::pFA-ARG4*	This study
CAS17	BWP17, *gal102:: GAL102/gal102::URA3, MET3::pFA-ARG4*	This study
CAS18	BWP17, *gal102:: gal102^K159A^/gal102::URA3, MET3::pFA-ARG4*	This study
*S. cerevisiae*		
PJB5	*MATa, ade2-101, ile, ura3-52, leu2-3112, trp1-HIII his3Δ-1,* MEL1gal10::LEU2	[60]

**Table 3:** List of plasmids.

Plasmid	Alias	Description	Reference
pVM602		*CaGAL102* in pGEM-Teasy (Promega)	This study
pPS189	pABE448	Yeast cloning vector, P_TEF1_, *URA3*, 2 µ	[61]
pMS643		*CaGAL102* under P_TEF1_ in pPS189	This study
pMS835		Codon optimized *CaGAL102* in pBSKS	This study
pMS836		Codon optimized *CaGAL102*subcloned in pET32(a) for expression	This study
pMS837		Codon optimised catalytic mutant of *CaGAL102* in pBSKS	This study
pMS838		Codon optimised catalytic mutant of *CaGAL102*subcloned in pET32(a) for expression	This study
pMS861		Codon optimized *CaGAL102*subcloned in pPS189	This study
pMS889		420 bp downstream region of *CaGAL102* cloned in pGEM-Teasy	This study
pMS890		420 bp downstream region of *CaGAL102*subcloned in pMS826	This study
pMS891		Wildtype Ca*GAL102*subcloned from pMS823(a) to pMS890 for genomic reintegration	This study
pMS892		Catalytic mutant of Ca*GAL102*subcloned in pMS890 for genomic reintegration	This study

In all assays such as mouse infection, etc. where number of cells is critical, the cells were counted using hemocytometer.

### Mice

Female BALB/c mice, aged 6–8 weeks and weighing ∼18–25 g, were obtained from the Central Animal Facility, Indian Institute of Science, Bangalore. All mice received care according to institutional guidelines and were maintained under controlled conditions and fed with a standard diet.

### DNA manipulation

*GAL102* (http://www.candidagenome.org/) was amplified with the CaputGAL10(f) and CaputGAL10(r) primers. The 963 bp fragment was cloned in pGEM-Teasy vector (Promega) to construct pVM602. pVM603 (*pGAL1-ORF19.3674*) was constructed by cloning of pYES2 (Invitrogen). pMS643 (*pTEF1-ORF19.3674*) was constructed by cloning *Bam*HI*/Xho*I insert from pVM603 in the same sites in pPS189. The construct thus obtained was used to transform *S. cerevisiae gal10* deletion strain, PJB5.Codon optimized *CaGAL102* was also cloned in the same sites in pPS189 (pMS861) and transformed in pJB5. A list of all the plasmids generated during this study is provided in Table 3.

To generate K159A mutation in Gal102, the first 490 bp of the wildtype open reading frame was amplified using Pfu (Fermentas) with primers CaputGAL10(f) and Gal102 _int _r (Table 4) harbouring the TTT to TGC change in the primer. The remaining 473 bp was amplified using *Pfu* (Fermentas) using primers Gal102 _int _f harbouring the AAA to GCA change and CaputGAL10(r). The final product was generated by overlap PCR using *Pfu* using primers CaputGAL10(f) and CaputGAL10(r) and cloned in pBSKS (Promega) in *Eco*RV site thus generating plasmid pMS833 The clone had K159A mutation in the Gal102 open reading frame and this was confirmed by sequencing. The Gal102 ^K159A^ open reading frame was released as a *Bam*HI-*Xho*I fragment from pMS833 and subcloned in pSJ821 in *Bgl*II-*Xho*I sites thus generating plasmid pMS834.

**Table 4:** List of primers.

Primer	Primer sequence
CaACT1(f)	5′CGTTGTTCCAATTTACGCTGG 3′
CaACT1(r)	5′CAGCAATACCTGGGAACATGG 3′
Ca putGal10 (r)	5′CAGTTCATGGCAAGGGAACC 3′
Ca put Gal10 int (f)	5′CAGTTCATGGCAAGGGAACC 3′
Ca put Gal10 _del _F	5′CTAAACTATGCTAGTAATGCTACTGAAATCGAAAATCTTAAGAGTTTCTCAAACTTTGAATTTGTTCACTTGGATTTATCAGAGAAGCTTCGTACGCTGCAGGTC 3′
Ca put Gal10_del _R	5′CCTTAACAAGAGATATTTTCGGAGACCATCCCAAATTATGGATCTTTTGTTGTGTCTATGGAATAATTAGTATCGTTGTAATTTCGATCTTTGATAAATTTCTGATATCATCGATGAATTGAG 3′
Gal102 CTG* r	5′ TTATTCGATGGGAGAGTCAAGCGATTGTTT 3′
Psfs2_f	5′GTCGAGCGTCAAAACTAGAG 3′
Psfs2_r	5′ GCAGGACCACCTTTGATTGT 3′
Gal102 _int _r	5′AATCAATAGCTGC**TGC**ACTTG 3′
Gal102 _int _f	5′CAAGT**GCA**GCAGCTATTGATT 3 ′
Ca putGal10 (f)	5′ GGGATCCCAACTTGACCTTAAAGG 3′

In order to express CaGal102 in *E. coli*, the only CUG codon in Gal102 (seventh codon from the C- terminus) was converted to other serine encoding codon to compensate for the alternative genetic code in *C. albicans.* The ORF was amplified using Caput Gal10 (f) and a reverse primer Gal102 CTG* r using *Pfu* polymerase (Fermentas) and cloned in pBSKS. The mutant CaGal102 hereafter referred to as Gal102^CTG*^,was further subcloned in pET32(a) to generate plasmid pMS836.To express the catalytic mutant in *E. coli*, the mutant ORF was PCR amplified using primers Ca put Gal10 (f) and Gal102 CTG* r from pMS833 (described above) and cloned in pET32(a) thus generating plasmid pMS838 (Table 3).

### Transformation of *C. albicans* and *S. cerevisiae*

The lithium acetate procedure as described previously was used to transform *C. albicans* [53] and *S. cerevisiae* [54]. For Nourseothricin based plasmids transformation was done by electroporation and selected on plates containing 200 µg/ml of nourseothricin at 30°C [55].

### Total protein extraction and Western Blot Analysis

Protein extracts were prepared from PJB5 cells transformed with pPS189 and pMS643 by glass bead lysis method. Protein samples were quantified using Bradford Reagent (Sigma) and approximately 50 µg of total protein was electrophoresed in 12.5% SDS-PAGE transferred to Immobilon^TM^-P Transfer Membrane (Amersham), and probed with rabbit polyclonal 1° antibodies raised against bacterially expressed orf19.3674p. The blots were probed with HRP-conjugated anti-rabbit 2° antibody (Sigma) and developed using Western Lighting ^TM^Chemiluminescence Reagent *plus* (Perkin Elmer) and X-ray film reagents.

### RNA isolation, RT PCR and microarray analysis

Strains were grown in YPD and YPGal at 30°C. Overnight saturated cultures were diluted in their respective media to an A_600_ of 0.1 and were allowed to grow until the A_600_ reached 1.2 following which total RNA was extracted as described below. WT SC5314 cells were induced to form hyphae using various hyphae inducing media described above. RNA was isolated by hot phenol method using liquid nitrogen [56]. RNA samples were quantified by measuring the absorbance at 260 nm and equal amounts of RNA were run on a 1% agarose-formaldehyde gel to check the quality. Total RNA was used for Genome wide expression profiling through Genotypic technology. Agilent's *C. albicans* custom arrays (AMADID 16356) were used and hybridized with the cDNAs that were labeled using the Agilent's RNA linear amplification kit following manufacturer's protocol. Two biological replicates were used for both WT and mutant NA samples and single color hybridization was performed on four independent arrays. The differentially expressed genes were identified using Genespring software based normalization methods (Typically LOWESS method was applied for array wide normalization and only mutant/WT ratio of >2 or <0.5 was considered as significant differential expression). The entire data has been deposited at the GEO database and can be accessed at http://www.ncbi.nlm.nih.gov/geo/query/acc.cgi?acc=GSE20883. (Text S2).

### Biofilm production and biofilm permeability assay

Biofilms were produced as described [57] and biofilm permeability assay was performed as described [17]. The concentration of echinocandin used for this assay was 300 µg/ml and 600 µg/ml.

### Scanning electron microscopy

Biofilm developed was fixed with 4% (v/v) formaldehyde made in 1X phosphate buffer (pH 7.5) for 15 min followed by washing with 1X phosphate buffer. Then the biofilm was treated with 1% osmium tetroxide made in 1X phosphate buffer for 1 hr in dark. Then the subsequent dehydration steps with ethanol were carried out as follows: 70% for 10 min, 95% for 10 min, 100% for 20 min, followed by air drying in a desiccator overnight prior to gold coating in a sputter.

### Spot assay for analyzing sensitivity to cell wall damaging agents

Cultures were grown in 5 ml of YPD medium until the exponential phase and then diluted to an optical density at 600 nm (OD_600_) of 0.5. Each undiluted culture and its four 10 fold serial dilutions were spotted onto YPD plates containing the following: Calcoflour White (10–40 µg/ml), sodium dodecyl sulfate (SDS; 0.005–0.04%), Congo red (5–30 µg/ml). Growth differences were recorded after incubation of the plates at 37°C for 72 hr.

### Preparation of mannans

*Candida* strains were grown at 30°C for 36 hr with shaking in YPD medium containing 1% yeast extract, 2% peptone and 2% dextrose. Mannan was extracted from the cells with water at 120°C in an autoclave for 3 hr [9]. Briefly, after centrifugation the supernatant was treated with equal volume of Fehling's reagent for a short term precipitation. The copper-mannan complex was dissolved in 3N HCl and further precipitated drop-wise in Methanol: Acetic acid (8∶1) solvent. The carbohydrate was dried, dissolved in water, purified and then lyophilized till further use.

### NMR spectroscopy of cell wall mannans

The mannans obtained so from the above mentioned procedure were then subjected to NMR analysis (all NMR studies were carried at the NMR Facility, Piramal Life Sciences Ltd., Mumbai, India). All NMR spectra were recorded for a solution sample of each (purified and lyophilized) mannan in 700 µl of deuterium oxide (D_2_O) at 40°C using BrukerAvance 500 MHz spectrometer equipped with *z*-gradient triple resonance (^1^H, ^13^C, X) probe. Acetone (δ_H_ 2.17 ppm) was used as an internal standard for the spectral referencing. The 2D TOCSY spectra (pulse, *mlevphpr*) were recorded containing 256 increments, consisting of 64 scans. The mixing time for each spectrum was set to 100 ms. 2D ^1^H-^13^C HSQC (pulse, *hsqcetgp)*) spectra were composed of 256 increments consisting of 64 scans. 2D ROESY spectra were acquired using a pulse sequence *roesyphpr*, with 256 increments consisting of 128 scans. 1D NMR spectra were recorded suing a standard pulse sequence, *zg30*. The ^31^P NMR spectra were recorded on Bruker 300 MHz spectrometer equipped with a BBO probe at the Piramal Life Sciences Ltd. research facility, Mumbai. Orthophosphoric acid was used as an external standard (δ^31^_P_ 7 ppm) for ^31^P NMR. All spectra were processed and analyzed using Topsin (version 2.1, Bruker, March 2007) program package.

### Co-culture studies with peritoneal macrophages and *C. albicans*

Cells were isolated from the peritoneal cavity of mice by flushing with 5 ml of ice-cold 0.32 M sucrose. 2×10^5^ cells/well were seeded in 96-well flat-bottom tissue culture plates in 100 µl/well culture medium (RPMI 1640, 5% FBS, 2 mM L-glutamine, 100 U/ml penicillin, 100 µg/ml streptomycin, 10 µg/ml gentamycin, 10% nonessential amino acids, 10% HEPES, 10% sodium pyruvate, 50 µM 2-ME). After incubation for 2 hr at 37°C with 5% CO_2_, wells were washed with medium to remove non-adherent cells and adherent cells were used for subsequent co-culture studies with *C. albicans* strains. Adherent resident peritoneal macrophages were incubated with 100 µl of various strains of live or heat-killed (30 min at 75°C) *C. albicans* cells at a concentration 10^4^ cells/ml. Supernatants were harvested at different time points (6, 18 and 30 hr) and stored at –80°C until analysis of cytokines. The experiments were performed in triplicate with samples from three mice. The differences between strains were analyzed by using the Student's *t* test, and the level of significance was set at p < 0.05.

### Infection of mice with *C. albicans* strains

Age-matched mice were challenged intravenously with 5×10^6^ *C. albicans* WT (SC5314/DAY286), *gal102Δ/Δ,* or WT or *gal102^K159A^* re-integrants in *gal102Δ/Δ* per mouse via lateral tail vein injection in 100 µl PBS. Control mice were injected with 100 µl PBS as vehicle control. Cohorts of five mice per *C. albicans* strain were inoculated for survival study. Mice were monitored daily for signs of morbidity (weight loss, ruffled fur, hunched appearance, and decreased activity).

### Tissue burden assay and histological examination

For the tissue burden assays same dose was injected as above (five mice per strain per experiment) and mice were sacrificed at varying time points (24, 42 and 60 hr) post-infection. Kidney and liver from individual mice (three to five mice per group) were removed aseptically, weighed and homogenized in 1X PBS buffer using homogenizer. CFU was determined by plating serial dilutions on YPD agar medium. The colonies were counted after 24 hr at 37°C, and results were expressed as log CFU/g tissue. These experiments were performed thrice with 5/6 mice per group. For histological examinations the kidney and liver tissues were dissected, fixed in 10% neutral formalin buffer and embedded in paraffin wax. The sections were mounted on slides and stained with haematoxylin-eosin (H&E). Examination was performed under a light microscope and photographs were taken by Nikon camera fitted to the microscope.

### Cytokine assays

The mice injected with WT (SC5314/DAY286), *gal102Δ/Δ,* or WT or *gal102^K159A^* re-integrants in *gal102Δ/Δ* or PBS was sacrificed at 24, 42 and 60 hr post-infection. Blood was collected by cardiac puncture, allowed to clot at room temperature and centrifuged (10,000 rpm for 10 min) to separate serum for subsequent assays. Cytokine concentrations were measured by commercial ELISA kits from eBioscience, San Diego, USA according to the manufacturer's instructions using 96 well plates. The serum samples were diluted 1∶5 and the concentrations of cytokines were determined, using a standard curve. The linear range of detection for TNFα, IFNγ and IL-4 was 31.25–1,000 pg/mL, 62.5–2,000 pg/mL and 15.125–500 pg/mL respectively. This experiment was performed thrice with 5 mice per group-per time point.

### UDP-glucose 4,6- dehydratase activity assay

The enzyme activity was measured as described [26]. Briefly, the recombinant protein expressed in *E. coli* was purified using Ni-NTA column and 20 µg of protein was incubated at 37°C for 2 hr in reaction buffer containing 50 mM Tris-Cl (pH-7.6). At various time points the A_340_ nm was measured. The assay was repeated with two independent purified enzyme preps. The results include data from three replicates.

### Statistical analysis

Data were analyzed using commercial software (GraphPad Prism 5 Software). Differences in *C. albicans* colonization of tissues and cytokine levels between two groups were analyzed by Student's *t* test and comparison between the survival curves were done using the Logrank test.

## Supporting Information

Figure S1 A) The *scgal10Δ* strain PJB5 was transformed with either the vector pPS189 or the *GAL102* orf expressing clone pMS643 and the codon optimized *GAL102* orf expressing clone pMS861. The proteins isolated from the transformants were blotted on nitrocellulose membrane and probed with anti Gal102p polyclonal serum. Single specific band of expected size was detected both in the pMS643 and pMS 861 transformants. B) Disruption of *GAL102* was carried out and confirmed as described in supplemental file materials and methods (Text S3). The genomic DNA from WT and homozygous strain *gal102Δ/Δ* was digested with HindIII- XbaI or AvaII and run on 1% agarose gel followed by blotting on nitrocellulose membrane and probed with the probe as described (Text S3). The total RNA isolated from the parent and the *gal102Δ/Δ* strain was probed with the same probe to show that the RNA could not be detected in *gal102Δ/Δ* mutant confirming the disruption of both alleles as indicated by the southern result. C) The *gal102Δ/Δ* mutant and the WT (SC5314) cells were grown on YPD. The effect on cell morphology is also reflected in the wrinkled colony morphology recorded after 3 days of growth at 37°C. D) The *gal102Δ/Δ* cells at 30°C show much more elongated cell morphology but at 25°C show morphology similar to *GAL102*.
(TIF)

Figure S2 ^31^P NMR spectra of mannans from *C. albicans* for the samples hyphal form (i), and the mutant *gal102Δ/Δ* (ii). The data was recorded at room temperature (25°C). The peak corresponding to the phosphodiester group in the mannan structural assembly is shown with a arrow mark. The peak is missing in samples corresponding to mutant form.
(TIF)

Figure S3 The analysis of 2D ^1^H-^1^H ROESY (2-dimesnional ^1^H-^1^H Rotating-frame Overhauser Effect SpectroscopY) spectra for both hyphal form and the mutant, further corroborated the loss of a portion of α-1,6 mannan backbone skeleton and other branched side chains of the cell wall mannans.
(TIF)

Figure S4 Resident mouse peritoneal macrophages were incubated for different time periods with either live or heat-killed WT or *gal102Δ/Δ*. The amounts of TNFα (A), IFNγ (B) and IL-4 (C) in the culture supernatants were measured as in case of the *in vivo* experiment. Experiments were set up in triplicate and repeated three times, with similar results. Results are presented as the mean ± SD. *p<0.05, **p<0.01 and ***p<0.005, *t* test.
(TIF)

Figure S5 Reduction in serum urea and ALT levels are observed upon infection of mice with *gal102Δ/Δ*. Mice were infected with WT or *gal102Δ/Δ* and were sacrificed at different time points after infection and serum urea (A) and ALT levels (B) were measured.
(TIF)

Figure S6 Kidney and liver tissue sections were dissected from mice sacrificed at 60 hr, stained with hematoxylin & eosin and histological changes were quantified as (A) percent immune cell infiltration and (B) percent area of inflammation by examining multiple fields. Sections from PBS treated mice were used as control. **p<0.01 compared with PBS (for WT injected mice) and ^##^ *p*<0.01, ^#^ *p*<0.05 compared with WT (for *gal102Δ/Δ* injected mice). Damage observed in *gal102Δ/Δ* injected mice was not significant compared to PBS.
(TIF)

Text S1 The compilation of microarray data in the form of two tables.
(DOC)

Text S2 The Microarray data was submitted to the GEO database. The relevant information on the experimental conditions are supplied as the GEO submission file information.
(DOC)

Text S3 Supplemental materials and methods.
(DOC)
