# Supplementary figures and images for: UDP-glucose 4, 6-dehydratase Activity Plays an Important Role in Maintaining Cell Wall Integrity and Virulence of Candida albicans
Source: PLoS Pathog. 2011 Nov 17;7(11):e1002384. doi: 10.1371/journal.ppat.1002384 (PMC3219719; doi:10.1371/journal.ppat.1002384)

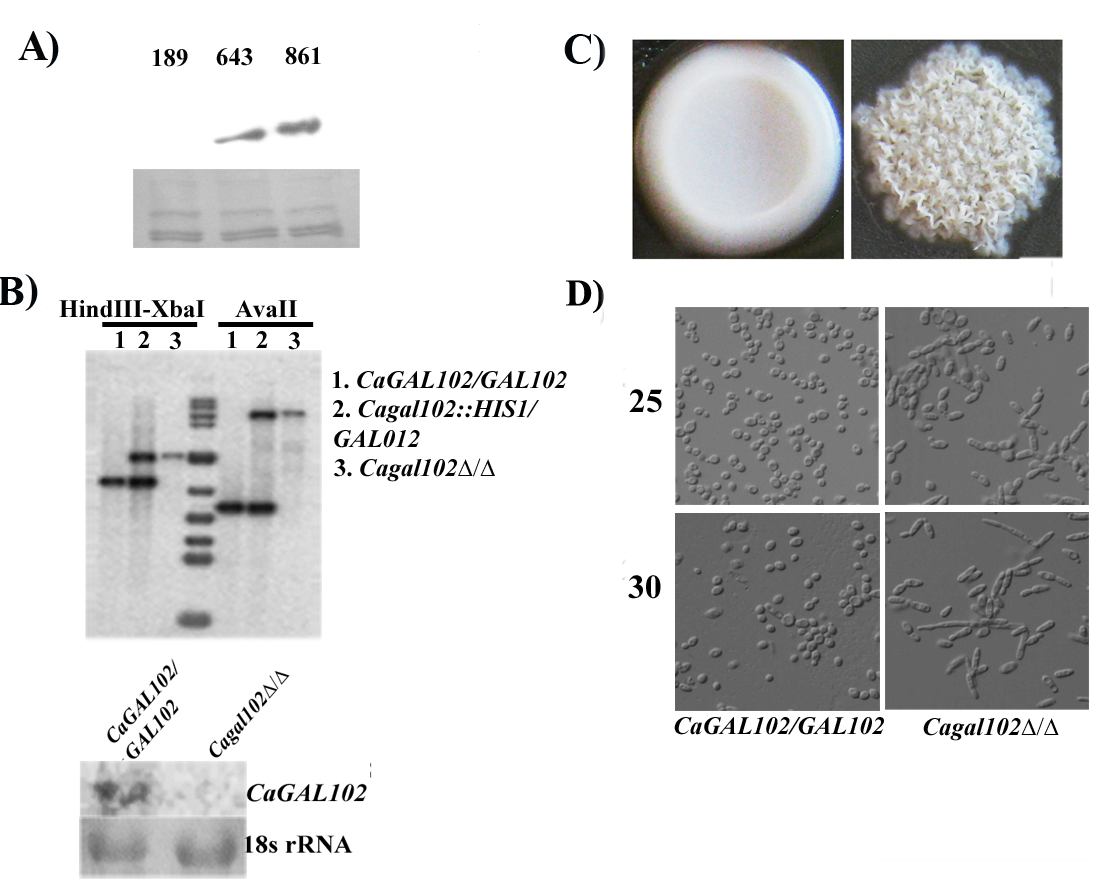

Supplement: Figure S1 — A) The scgal10Δ strain PJB5 was transformed with either the vector pPS189 or the GAL102 orf expressing clone pMS643 and the codon optimized GAL102 orf expressing clone pMS861. The proteins isolated from the transformants were blotted on nitrocellulose membrane and probed with anti Gal102p polyclonal serum. Single specific band of expected size was detected both in the pMS643 and pMS 861 transformants. B) Disruption of GAL102 was carried out and confirmed as described in supplemental file materials and methods (Text S3). The genomic DNA from WT and homozygous strain gal102Δ/Δ was digested with HindIII- XbaI or AvaII and run on 1% agarose gel followed by blotting on nitrocellulose membrane and probed with the probe as described (Text S3). The total RNA isolated from the parent and the gal102Δ/Δ strain was probed with the same probe to show that the RNA could not be detected in gal102Δ/Δ mutant confirming the disruption of both alleles as indicated by the southern result. C) The gal102Δ/Δ mutant and the WT (SC5314) cells were grown on YPD. The effect on cell morphology is also reflected in the wrinkled colony morphology recorded after 3 days of growth at 37°C. D) The gal102Δ/Δ cells at 30°C show much more elongated cell morphology but at 25°C show morphology similar to GAL102. (TIF) [file ppat.1002384.s001.tif]

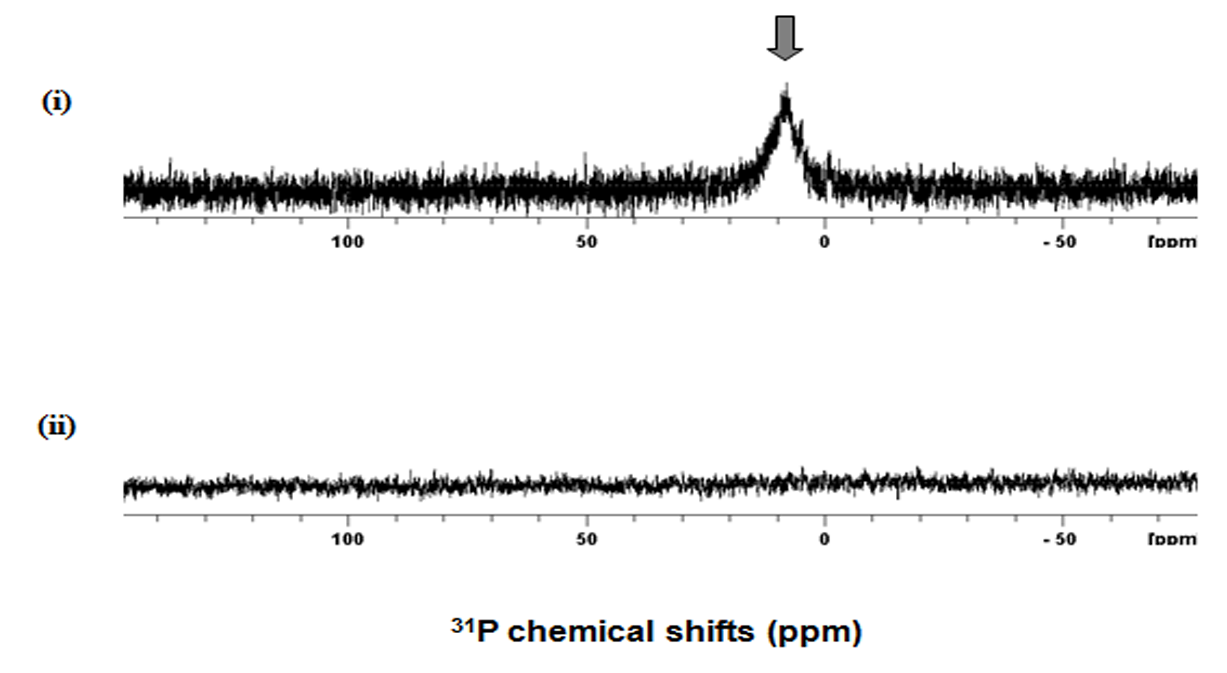

Supplement: Figure S2 — 31P NMR spectra of mannans from C. albicans for the samples hyphal form (i), and the mutant gal102Δ/Δ (ii). The data was recorded at room temperature (25°C). The peak corresponding to the phosphodiester group in the mannan structural assembly is shown with a arrow mark. The peak is missing in samples corresponding to mutant form. (TIF) [file ppat.1002384.s002.tif]

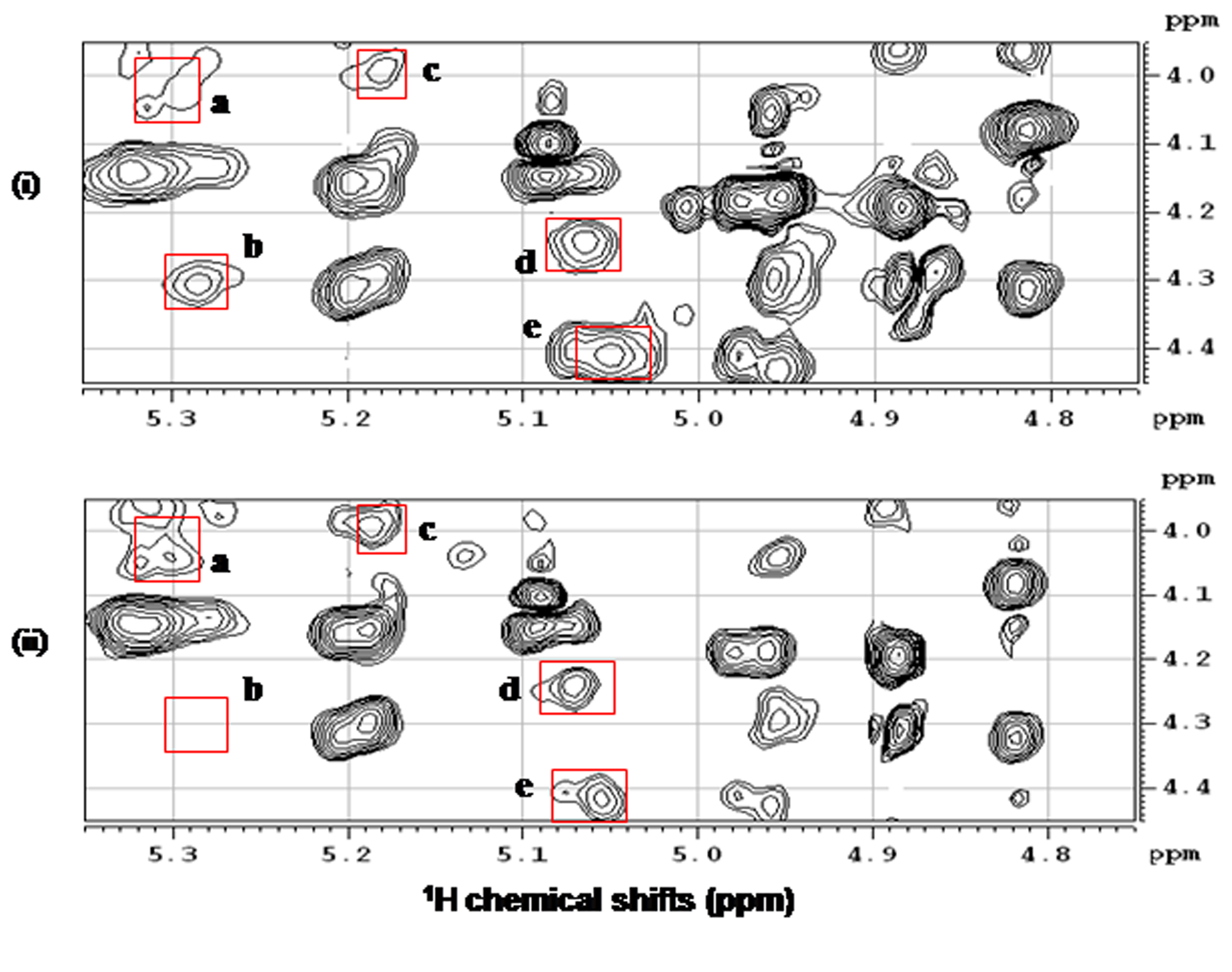

Supplement: Figure S3 — The analysis of 2D 1H-1H ROESY (2-dimesnional 1H-1H Rotating-frame Overhauser Effect SpectroscopY) spectra for both hyphal form and the mutant, further corroborated the loss of a portion of α-1,6 mannan backbone skeleton and other branched side chains of the cell wall mannans. (TIF) [file ppat.1002384.s003.tif]

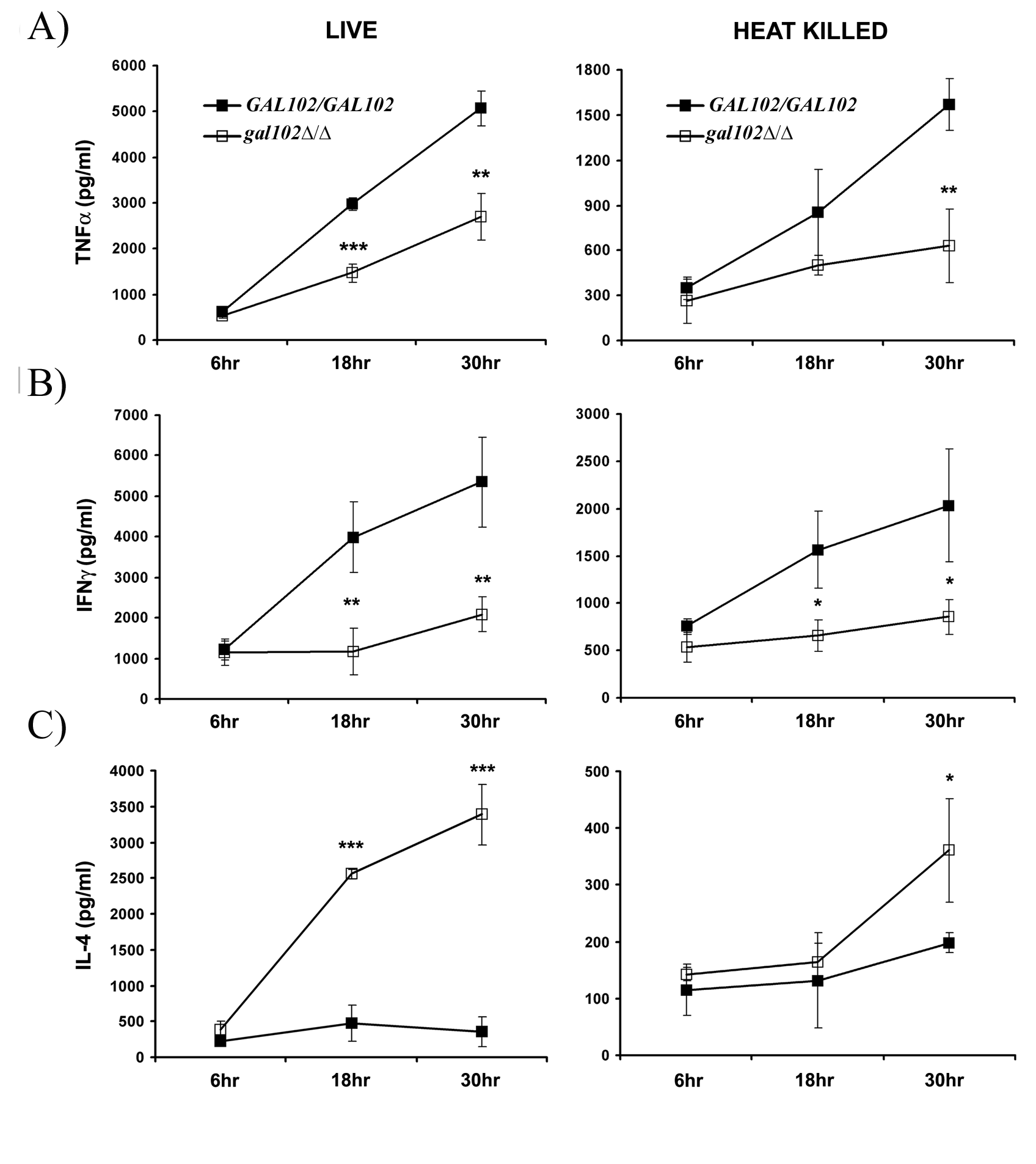

Supplement: Figure S4 — Resident mouse peritoneal macrophages were incubated for different time periods with either live or heat-killed WT or gal102Δ/Δ. The amounts of TNFα (A), IFNγ (B) and IL-4 (C) in the culture supernatants were measured as in case of the in vivo experiment. Experiments were set up in triplicate and repeated three times, with similar results. Results are presented as the mean ± SD. *p<0.05, **p<0.01 and ***p<0.005, t test. (TIF) [file ppat.1002384.s004.tif]

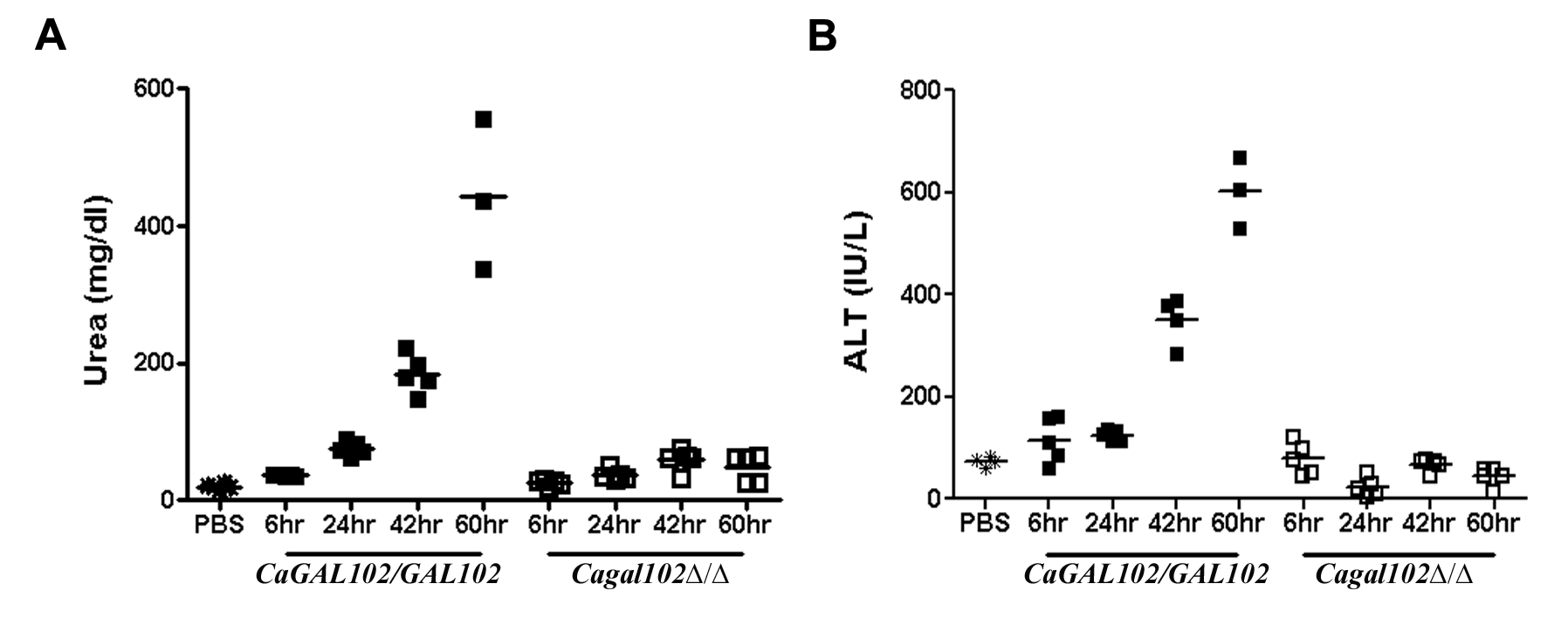

Supplement: Figure S5 — Reduction in serum urea and ALT levels are observed upon infection of mice with gal102Δ/Δ. Mice were infected with WT or gal102Δ/Δ and were sacrificed at different time points after infection and serum urea (A) and ALT levels (B) were measured. (TIF) [file ppat.1002384.s005.tif]

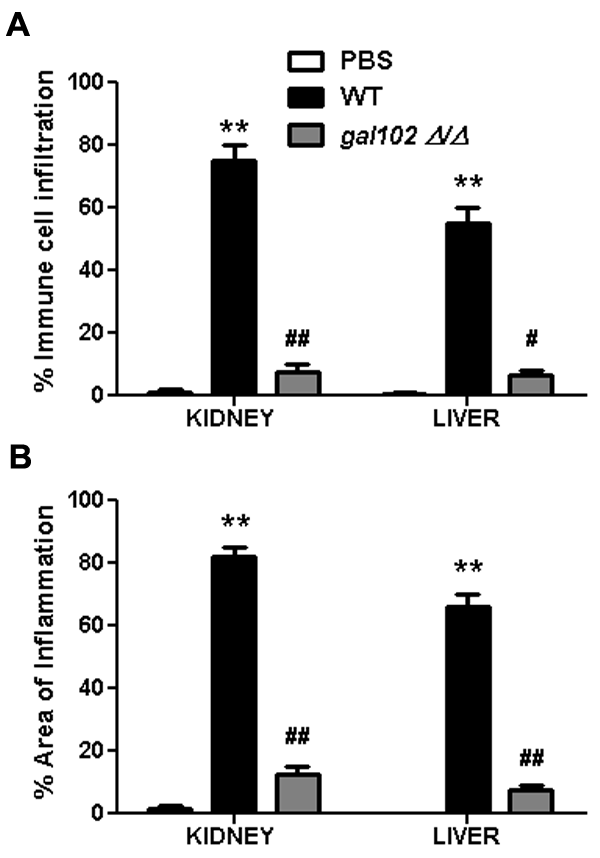

Supplement: Figure S6 — Kidney and liver tissue sections were dissected from mice sacrificed at 60 hr, stained with hematoxylin & eosin and histological changes were quantified as (A) percent immune cell infiltration and (B) percent area of inflammation by examining multiple fields. Sections from PBS treated mice were used as control. **p<0.01 compared with PBS (for WT injected mice) and ## p<0.01, # p<0.05 compared with WT (for gal102Δ/Δ injected mice). Damage observed in gal102Δ/Δ injected mice was not significant compared to PBS. (TIF) [file ppat.1002384.s006.tif]
