# Supplementary material for: UDP-glucose 4, 6-dehydratase Activity Plays an Important Role in Maintaining Cell Wall Integrity and Virulence of Candida albicans
Source: PLoS Pathog. 2011 Nov 17;7(11):e1002384. doi: 10.1371/journal.ppat.1002384 (PMC3219719; doi:10.1371/journal.ppat.1002384)
Supplement: Text S1 — The compilation of microarray data in the form of two tables. (DOC) [file ppat.1002384.s007.doc]

**Table S1.** List of major functional categories of probes for genes differentially expressed in the *gal102* mutant as compared to the WT. The significance of the representation of these functional categories is indicated by the p value.

| Category | Probes in Category | % of Probes in Category | Probes in List in Category | % of probes in List in Category | p-Value |  |
| --- | --- | --- | --- | --- | --- | --- |
| GO:31224: intrinsic to membrane | 384 | 8.083 | 42 | 16.6 | 3.52E-06 | Down regulated genes |
| GO:16021: integral to membrane | 368 | 7.746 | 39 | 15.42 | 1.76E-05 |
| GO:44010: single-species biofilm formation | 17 | 0.343 | 7 | 2.31 | 3.39E-05 |
| GO:15891: siderophore transport | 3 | 0.0605 | 3 | 0.99 | 0.0002 |
| GO:42928: ferrichrome transport | 3 | 0.0605 | 3 | 0.99 | 0.0002 |
| GO:7162: negative regulation of cell adhesion | 3 | 0.0605 | 3 | 0.99 | 0.0002 |
| GO:5886: plasma membrane | 456 | 9.598 | 42 | 16.6 | 0.0002 |
| GO:44011: single-species biofilm formation on inanimate substrate | 16 | 0.323 | 6 | 1.98 | 0.0002 |
| GO:4727: prenylated protein tyrosine phosphatase activity | 3 | 0.0659 | 3 | 1.024 | 0.0003 |
| GO:31226: intrinsic to plasma membrane | 103 | 2.168 | 15 | 5.929 | 0.0003 |
| GO:42710: biofilm formation | 32 | 0.645 | 8 | 2.64 | 0.0005 |
| GO:33: alpha-1,3-mannosyltransferase activity | 12 | 0.264 | 5 | 1.706 | 0.0006 |
| GO:6493: protein amino acid O-linked glycosylation | 33 | 0.666 | 8 | 2.64 | 0.0006 |
| GO:9277: cell wall (sensu Fungi) | 101 | 2.126 | 14 | 5.534 | 0.0008 |
|  |  |  |  |  |  |  |
| GO:5618: cell wall | 130 | 2.736 | 30 | 8 | 4.50E-08 | Up regulated genes |
| GO:6092: main pathways of carbohydrate metabolism | 83 | 1.674 | 22 | 6.061 | 6.11E-08 |
| GO:15980: energy derivation by oxidation of organic compounds | 175 | 3.53 | 34 | 9.366 | 8.09E-08 |
| GO:5975: carbohydrate metabolism | 86 | 1.735 | 22 | 6.061 | 1.22E-07 |
| GO:9986: cell surface | 261 | 5.494 | 44 | 11.73 | 6.73E-07 |
| GO:16614: oxidoreductase activity, acting on CH-OH group of donors | 84 | 1.845 | 21 | 5.983 | 8.39E-07 |
| GO:16616: oxidoreductase activity, acting on the CH-OH group of donors, NAD or NADP as acceptor | 72 | 1.581 | 19 | 5.413 | 1.16E-06 |
| GO:9277: cell wall (sensu Fungi) | 101 | 2.126 | 23 | 6.133 | 2.27E-06 |
| GO:45026: plasma membrane fusion | 7 | 0.141 | 5 | 1.377 | 3.80E-05 |
| GO:30446: hyphal cell wall | 56 | 1.179 | 14 | 3.733 | 7.70E-05 |
| GO:8194: UDP-glycosyltransferase activity | 32 | 0.703 | 7 | 1.994 | 0.0095 |
| GO:5199: structural constituent of cell wall | 18 | 0.395 | 5 | 1.425 | 0.0098 |

**Table S2**

The differentially regulated genes in the absence of *gal102* which show at least two fold higher or lower expression as compared to WT are listed below. The average value of extent up and down regulation is given as the ratio of normalized intensities.

| Upregulated genes | | |
| --- | --- | --- |
| Ratio | Common | Description |
| 2.450203 | CaACO1 | aconitate hydratase (by homology) |
| 2.285814 | CaACR1 | Succinate-fumarate transporter (by homology) |
| 5.429188 | CaADH2 | alcohol dehydrogenase I (by homology) |
| 2.985789 | CaAGP3 | amino acid-permease (by homology) |
| 2.326142 | CaALD4 | aldehyde dehydrogenase, mitochondrial (by homology) |
| 2.774934 | CaALG5 | dolichol-P-glucose synthetase (by homology) |
| 4.143688 | CaALK2 | n-alkane inducible cytochrome P-450 (by homology) |
| 2.097055 | CaALS3.3eoc | agglutinin-like protein, 3-prime end |
| 72.43881 | CaALS6 | agglutinin-like protein |
| 6.506462 | CaALS7 | agglutinin-like protein |
| 2.468711 | CaAMYG1 | glucoamylase |
| 2.835994 | CaAMYG2 | glucoamylase |
| 3.119267 | CaAOX1 | alternative oxidase (by homology) |
| 2.051304 | CaAOX2 | alternative oxidase (by homology) |
| 2.366972 | CaAPL3 | AP-2 complex subunit, alpha-adaptin (by homology) |
| 2.732247 | CaAPR1 | aspartyl protease |
| 2.133451 | CaAPS3 | AP-3 complex subunit, sigma3 subunit (by homology) |
| 3.954825 | CaARA1 | D-arabinose dehydrogenase (by homology) |
| 2.892808 | CaARE2 | acyl-CoA sterol acyltransferase-like (by homology) |
| 3.193682 | CaARG2 | acetylglutamate synthase (by homology) |
| 2.358039 | CaARG81 | transcription factor possibly involved in arginine metabolism, 3-prime end (by homology) |
| 2.642821 | CaARG81.3eoc | transcription factor possibly involved in arginine metabolism, 3-prime end (by homology) |
| 2.186771 | CaARO9 | aromatic amino acid aminotransferase II(by homology) |
| 2.68102 | CaATP1.exon3 | F1F0-ATPase complex, F1 alpha subunit, exon 3 |
| 2.138332 | CaATP14.exon2 | F1F0-ATPase complex, subunit h, exon 2 (by homology) |
| 2.229109 | CaATP17.3 | F1F0-ATPase complex, F1 delta subunit f, 3-prime end (by homology) |
| 2.18995 | CaATP4 | F1F0-ATPase complex, F0 subunit B (by homology) |
| 2.887633 | CaBIO2 | biotin synthetase (by homology) |
| 5.904349 | CaBMR1 | benomyl/methothrexate resistance protein (by homology) |
| 3.806851 | CaBNI1 | regulator of budding (by homology) |
| 2.617429 | CaCAN5 | amino acid permease (by homology) |
| 2.546946 | CaCAT8 | transcription factor involved in gluconeogenesis (by homology) |
| 2.289882 | CaCDC10 | cell division control protein |
| 2.764774 | CaCDC21 | thymidylate synthase (by homology) |
| 2.964049 | CaCDC3 | Cell division control protein |
| 2.071169 | CaCDC43 | geranylgeranyltransferase I |
| 2.487745 | CaCDC46 | cell division control protein (by homology) |
| 2.718194 | CaCDC48 | microsomal ATPase (by homology) |
| 2.815655 | CaCDC8 | dTMP kinase (by homology) |
| 10.20743 | CaCDC9 | DNA ligase (by homology) |
| 2.851079 | CaCHL1 | protein of the DEAH box family (by homology) |
| 2.574418 | CaCHL4 | chromosome segregation protein (by homology) |
| 5.719065 | CaCHS21 | Chitin synthase (by homology) |
| 2.538593 | CaCHT1 | endochitinase 1 precursor |
| 2.998365 | CaCHT2 | chitinase 2 precursor |
| 9.046662 | CaCirt3 | Putative transposase |
| 2.193247 | CaCOX5A | cytochrome-c oxidase chain V.A precursor (by homology) |
| 2.420909 | CaCOX7 | Subunit VII of cytochrome c oxidase (by homology) |
| 2.984477 | CaCOX9 | CYTOCHROME C OXIDASE (by homology) |
| 2.189763 | CaCPR3 | cyclophilin (peptidylprolyl isomerase), mitochondrial (by homology) |
| 3.563827 | CaCPR6 | cyclophylin (by homology) |
| 3.162722 | CaCRH11 | Probable membrane protein (by homology) |
| 2.592459 | CaCYC1 | cytochrome-c isoform 1 |
| 3.204485 | CaDAL1 | allantoinase |
| 2.251006 | CaDAL2 | Allantoinase |
| 2.223561 | CaDAL52 | allantoate permease (by homology) |
| 4.512262 | CaDAL53 | allantoate permease (by homology) |
| 5.695323 | CaDBF2 | putative ser/thr protein kinase by homology |
| 3.859728 | CaDIP53.exon2 | dicarboxylic amino acid permease, exon 2 (by homology) |
| 7.425782 | CaDIT2 | putative cytochrome P450 |
| 2.845775 | CaDOG2 | 2-deoxyglucose-6-phosphate phosphatase (by homology) |
| 2.41318 | CaDPB4 | DNA-directed DNA polymerase epsilon, subunit D (by homology) |
| 2.789882 | CaDPM1 | dolichol-phosphate (beta-D) mannosyltransferase 1 by homology |
| 2.194162 | CaDPP1 | Diacylglycerol Pyrophosphate Phosphatase by homology |
| 2.348681 | CaDUR34 | Urea transport protein (by homology) |
| 3.409036 | CaDYN1 | dynein heavy chain, cytosolic (by homology) |
| 2.077139 | CaECM21.3 | Involved in cell wall biogenesis and architecture (by homology) |
| 2.967599 | CaELC1 | Transcription elongation factor (by homology) |
| 2.344111 | CaERG2 | C-8 sterol isomerase |
| 2.613924 | CaERK2 | mitogen-activated protein kinase (MAP kinase) |
| 2.259398 | CaERV1.3 | Mitochondrial biogenesis and regulation of cell cycle, 3-prime end (by homology) |
| 6.800664 | CaEXG2 | glucan 1,3-beta-glucosidase-like by homology |
| 2.117525 | CaFAA21 | long-chain-fatty-acid-CoA ligase (by homology) |
| 2.801424 | CaFAB1 | phosphatidylinositol 3-phosphate 5-kinase (by homology) |
| 3.235741 | CaFAD1 | flavin adenine dinucleotide (FAD) synthetase (by homology) |
| 3.085784 | CaFBP1 | Fructose-1,6-bisphosphatase |
| 3.185217 | CaFCY24 | Putative purine-cytosine transport protein |
| 5.204153 | CaFDH11.3 | glutathione-dependent formaldehyde dehydrogenase, 3-prime end (by homology) |
| 14.06136 | CaFDH12 | Formate dehydrogenase (by homology) |
| 11.08349 | CaFDH2 | Formate dehydrogenase (by homology) |
| 11.21542 | CaFDH4.3f | Formate dehydrogenase, 3-prime end (by homology) |
| 2.80392 | CaFUM11 | fumarate hydratase |
| 2.495674 | CaGAD1 | Glutamate decarboxylase (by homology) |
| 2.475781 | CaGAL1 | galactokinase |
| 2.477038 | CaGDH2 | NAD-specific glutamate dehydrogenase (NAD) (by homology) |
| 3.387887 | CaGDH3 | NADP-glutamate dehydrogenase (by homology) |
| 4.685247 | CaGEF2 | Putative voltage-gated chloride channel protein (by homology) |
| 2.686661 | CaGND1 | 6-phosphogluconate dehydrogenase |
| 2.438934 | CaGPI8 | essential for GPI anchor attachment (by homology) |
| 7.99635 | CaGPX1 | glutathione peroxidase (by homology) |
| 2.558876 | CaGRP1 | dihydroflavonol-4-reductases (by homology) |
| 3.037578 | CaGRP2 | Reductase (by homology) |
| 2.323132 | CaGRX5 | Glutaredoxin |
| 2.143246 | CaGTT1.3 | glutathione S-transferase, 3-prime end (by homology) |
| 2.232181 | CaHEM4 | uroporphyrinogen III synthase |
| 2.661622 | CaHGT12 | hexose transporter |
| 2.107939 | CaHHF22 | histone H4 (by homology) |
| 2.476101 | CaHHT22 | Histone H3 |
| 4.143961 | CaHNT2 | Diadenosine polyphosphate hydrolase |
| 4.386406 | CaHSP10.3 | 10 kDa mitochondrial heat shock chaperonin, 3-prime end (by homology) |
| 2.492325 | CaHSP104 | Heat shock protein (by homology) |
| 5.159032 | CaHSP31 | heat shock protein (by homology) |
| 2.795415 | CaHSP90 | heat shock protein |
| 2.747663 | CaHTA1 | Histone H2A (by homology) |
| 26.74686 | CaHWP1 | Hyphal wall protein |
| 2.7864 | CaHYU1 | hydantoin utilization protein A (hyuA) homolog |
| 2.269712 | CaIFD2 | putative oxidoreductase (by homology) |
| 2.910118 | CaIFD3 | OXIDOREDUCTASE (by homology) |
| 3.58419 | CaIFD4 | Putative aryl-alcohol dehydrogenase (by homology) |
| 6.36356 | CaIFD5 | Putative aryl-alcohol dehydrogenase (by homology) |
| 11.02278 | CaIFD6 | Putative aryl-alcohol dehydrogenase (by homology) |
| 2.55076 | CaIFK1 | probable monoxygenase (by homology) |
| 2.590769 | CaIFK2 | probable monoxygenase (by homology) |
| 5.802471 | CaIFK3 | Probable monooxygenase (by homology) |
| 3.36174 | CaIFM3 | 2-hydroxyacid dehydrogenase by homology, Glycerate-formate-dehydrogenases-like |
| 2.631205 | CaIFN1 | glycerophosphoinositol transporter (by homology) |
| 4.412523 | CaIFN2 | glycerophosphoinositol transporter (by homology) |
| 7.46327 | CaIFS4 | Pirin protein (by homology) |
| 5.614825 | CaIPL1 | Ser/thr protein kinase (by homology) |
| 2.417214 | CaIRR1.3f | cohesin complex subunit, 3-prime end (by homology) |
| 2.808061 | CaKGD1 | 2-oxoglutarate dehydrogenase |
| 3.257783 | CaKGD2 | 2-oxoglutarate dehydrogenase complex E2 component (by homology) |
| 4.415841 | CaKIP3 | Kinesin-related protein required for nuclear migration (by homology) |
| 2.971382 | CaLAP41 | aminopeptidase yscI precursor (by homology) |
| 3.076912 | CaLAS17 | actin assembly factor (by homology) |
| 9.190227 | CaLIP1 | Secretory lipase |
| 4.106986 | CaLIP4 | secretory lipase |
| 2.78303 | CaLSC1 | succinate-CoA ligase / synthetase (by homology) |
| 2.216829 | CaMAK3 | N-acetyltransferase (by homology) |
| 2.794582 | CaMCM2 | replication licensing factor (by homology) |
| 2.716607 | CaMDH11 | Malate dehydrogenase (by homology) |
| 2.760037 | CaMGM101 | mitochondrial genome maintenance protein (by homology) |
| 2.778861 | CaMIR1 | phosphate transport protein, mitochondrial (MCF) (by homology) |
| 2.614806 | CaMNN3 | Golgi alpha-1,2-mannosyltransferase (by homology) |
| 2.2052 | CaMNN4 | regulates the mannosylphosphorylation (by homology) |
| 2.799948 | CaMSL5 | branch point bridging protein (by homology) |
| 2.467748 | CaNDH2 | NADH dehydrogenase (by homology) |
| 2.410083 | CaNIT3 | nitrilase (by homology) |
| 2.153569 | CaNPL4 | nuclear protein localization factor and ER translocation component (by homology) |
| 2.215272 | CaNPR1 | nitrogen permease reactivator protein (by homology) |
| 6.268751 | CaNRG1 | similar to transcriptional repressor Nrg1p/Nrg2p |
| 2.429281 | CaOGG1 | 8-oxoguanine DNA glycosylase (by homology) |
| 8.244402 | CaOPT2.53f | Oligopeptide transporter, internal fragment (by homology) |
| 2.167672 | CaORC1 | Origin recognition complex protein 1 |
| 2.691044 | CaPCK1 | phosphoenolpyruvate carboxykinase |
| 2.111247 | CaPCM1 | phosphoacetylglucosamine mutase (by homology) |
| 2.622631 | CaPET117 | cytochrome c oxidase assembly factor (by homology) |
| 48.15725 | CaPHO12 | Acid phosphatase, secreted (by homology) |
| 5.090099 | CaPHO8.5 | repressible alkaline phosphatase, 5-prime end (by homology) |
| 2.309588 | CaPLB4.3f | phospholipase, 3-prime end (by homology) |
| 2.029388 | CaPMM1 | phosphomannomutase |
| 2.900701 | CaPOL12 | DNA-directed DNA polymerase alpha (by homology) |
| 2.094282 | CaPOL30 | Proliferating Cell Nuclear Antigen (by homology) |
| 3.591612 | CaPOR1 | mitochondrial outer membrane porin (by homology) |
| 3.119605 | CaPPH3.3b | protein serine/threonine phosphatase, 3-prime end (by homology) |
| 3.208866 | CaPPR1 | transcription factor regulating pyrimidine pathway (by homology) |
| 3.375476 | CaPRA1 | pH-regulated antigen |
| 2.542576 | CaPRB2 | Protease B, vacuolar (by homology) |
| 2.122378 | CaPRC3 | Carboxypeptidase Y precursor (by homology) |
| 2.933408 | CaPRO3 | delta 1-pyrroline-5-carboxylate reductase (by homology) |
| 3.265264 | CaPRY2 | putative pathogen related proteins (by homology) |
| 3.128782 | CaPST2 | 1,4-benzoquinone reductase by homology |
| 3.842064 | CaPXP2 | acyl-CoA oxidase peroxisomal (by homology) |
| 3.153629 | CaPYC2.exon2 | Pyruvate carboxylase 2, exon 2 (by homology) |
| 2.740593 | CaQCR2 | Ubiquinol--cytochrome-c reductase 40KD chain II (by homology) |
| 2.867015 | CaQCR8 | ubiquinol-cytochrome-c reductase chain VIII (by homology) |
| 3.346826 | CaRAD51 | DNA repair protein by homology |
| 2.406269 | CaRAM2 | geranylgeranyltransferase type I alpha subunit |
| 3.173598 | CaRBL2 | Beta-tubulin binding protein (by homology) |
| 16.37792 | CaRBT1 | repressed by TUP1 protein 1 |
| 2.511421 | CaREV1 | DNA repair protein (by homology) |
| 2.218577 | CaRFA1 | DNA replication factor A, 69 KD subunit (by homology) |
| 2.646115 | CaRFA2 | DNA replication factor by homology to S. cerevisiae |
| 2.798273 | CaRFC3 | DNA replication factor C, 40 kDa subunit (by homology) |
| 3.097981 | CaRFC5 | DNA replication factor C (by homology) |
| 2.747352 | CaRGA2 | rho-GTPase activating protein 2 (by homology) |
| 2.758679 | CaRIB1 | GTP cyclohydrolase II by homology to S.cerevisiae |
| 2.564073 | CaRIB4.3 | 6,7-dimethyl-8-ribityllumazine synthase, 3-prime end (by homology) |
| 3.368152 | CaRIM1 | telomere-binding protein (by homology) |
| 2.423101 | CARIP1 | Ubiquinol cytochrome-c reductase (by homology) |
| 3.82997 | CaRNH35 | RNase H (by homology) |
| 2.14605 | CaRNR21 | ribonucleoside-diphosphate reductase (by homology) |
| 3.783508 | CaRPL10E | Ribosomal protein L10, cytosolic (by homology) |
| 2.670432 | CaRPP1B | Acidic ribosomal protein L44 (by homology) |
| 2.786426 | CaSAP1 | secreted aspartyl proteinase |
| 5.483947 | CaSAP4 | secreted aspartyl proteinase |
| 8.491717 | CaSAP6 | secreted aspartyl protease |
| 2.094779 | CaSAS2 | Zinc finger protein involved in silencing (by homology) |
| 4.293885 | CaSBA1 | Hsp90 (Ninety) Associated Co-chaperone (by homology) |
| 2.477122 | CaSDH41 | succinate dehydrogenase membrane anchor subunit for sdh2p (by homology) |
| 3.464436 | CaSDS24 | Similar to S. cerevisiae YBR214w which presents strong similarity to hypothetical protein YGL056c |
| 2.519458 | CaSEC11 | signal peptidase subunit (by homology) |
| 2.086992 | CaSIS1 | heat shock protein (by homology) |
| 2.822278 | CaSKP1 | kinetochore protein complex CBF3 by homology |
| 3.402263 | CaSMC2 | chromosome segregation protein (by homology) |
| 2.711507 | CaSMC3 | required for structural maintenance of chromosomes (by homology) |
| 2.475577 | CaSMD2 | U1 snRNP protein of the Sm class protein (by homology) |
| 2.457454 | CaSMI1 | beta-1,3-glucan synthesis protein (by homology) |
| 2.764525 | CaSNQ2 | multidrug resistance protein (by homology) |
| 2.466701 | CaSNZ1 | stationary phase protein by homology |
| 2.831134 | CaSPC3 | signal peptidase subunit (by homology) |
| 2.175806 | CaSPE3 | putrescine aminopropyltransferase by homology |
| 2.345135 | CaSPO70.3f | involved in meiosis and sporulation, 3-prime end |
| 2.116545 | CaSPO72 | required for sporulation (by homology) |
| 8.933435 | CaSPR1 | exo-1,3-beta-glucanase precursor (by homology) |
| 6.295437 | CaSSA1 | Heat shock protein of HSP70 family |
| 3.457914 | CaSSA4 | cahsp70 mRNA for heat shock |
| 2.815298 | CaSSC1 | Mitochondrial heat shock protein 70-related protein (by homology) |
| 2.703573 | CaSTL2.3f | sugar transporter, 3-prime end (by homology) |
| 3.497382 | CaSUL1 | High-affinity sulfate transport protein (by homology) |
| 2.316381 | CaSWI4 | transcription factor (by homology) |
| 4.949105 | CaSWI6 | Transcription factor (by homology) |
| 2.65675 | CaTAD3 | tRNA-specific adenosine-34 deaminase subunit (by homology) |
| 2.54759 | CaTAL1 | transaldolase (by homology) |
| 2.103636 | CaTEM1 | GTP-binding protein of the RAS superfamily |
| 3.86168 | CaTERT2 | telomerase reverse transcriptase 2 |
| 2.295221 | CaTHI80 | Thiamin pyrophosphokinase (by homology) |
| 2.7367 | CaTHO1 | suppressor of the hpr1 ts phenotype (by homology) |
| 4.720603 | CaTIM22 | Mitochondrial import inner membrane translocase subunit (by homology) |
| 3.091502 | CaTOF1 | Topoisomerase I interacting factor 1 (by homology) |
| 4.57668 | CaTOP3 | DNA topoisomerase III (by homology) |
| 2.527469 | CaTPM2.3 | Tropomyosin, 3-prime end |
| 2.874536 | CaTRS23 | targeting and fusion of ER to golgi transport vesicles by homology |
| 3.406488 | CaTUB4.3 | gamma-tubulin, 3-prime end |
| 3.999948 | CaUGA11.exon2 | 4-aminobutyrate aminotransferase, exon 2 (by homology) |
| 3.200664 | CaUGT51 | UDP-glucose:sterol glucosyltransferase |
| 3.40316 | CaUNG1 | Uracil-DNA glycosylase (by homology) |
| 2.606427 | CaURH1 | Uridine ribohydrolase (by homology) |
| 3.35113 | CaVMA1 | vacuolar ATPase V1 domain subunit G |
| 2.836828 | CaVPH2 | H+-ATPase assembly protein (by homology) |
| 2.483427 | CaVPS8 | Vacuolar sorting protein (by homology) |
| 3.708798 | CaXKS1 | xylulokinase (by homology) |
| 2.533424 | CaYAK1 | Ser/thr protein kinase (by homology) |
| 2.141497 | CaYDJ1 | Mitochondrial and ER import protein (by homology) |
| 3.209303 | CaYHC1 | SMALL NUCLEAR RIBONUCLEOPROTEIN C (by homology) |
| 2.303344 | CaYKE2.3 | Gim complex component, 3-prime end (by homology) |
| 2.247719 | CaYPT1 | GTP-binding protein of the rab family (by homology) |
| 2.629386 | CaYPT71 | GTP-binding protein of the RAB family (by homology) |
| 2.62342 | IPF10077 | 3-oxoacid CoA-transferase by homology |
| 5.990964 | IPF1009 | Weak similarity to S. cerevisiae RFX1 |
| 4.08612 | IPF10223 | putative serine/threonine kinase |
| 2.17826 | IPF10258 | similar to Saccharomyces cerevisiae Rad24p cell cycle checkpoint protein (by homology) |
| 2.304846 | IPF10422 | Similar to APG12, component of the autophagic system |
| 3.026201 | IPF10866 | similar to Saccharomyces cerevisiae Ira2p GTPase-activating protein for RAS proteins (by homology) |
| 2.542109 | IPF10919 | Similar to Flo1p (by homology) |
| 2.402245 | IPF11123 | similar to Saccharomyces cerevisiae Sdh2p succinate dehydrogenase iron-sulfur protein subunit (by homology) |
| 2.576223 | IPF11271 | by homology to S. cerevisiae: ATP19 subunit K of the dimeric form of mitochondrial F1F0-ATP synthase |
| 9.248744 | IPF11281 | similar to Saccharomyces cerevisiae Gpr1p G-protein coupled receptor (by homology) |
| 2.185233 | IPF11550.3f | Ca2+-transporting P-type ATPase, 3-prime end (by homology) |
| 5.21047 | IPF11589 | similar to Saccharomyces cerevisiae Pho13p 4-nitrophenylphosphatase |
| 3.474406 | IPF11598 | by homology to S. cerv.:hexokinase I |
| 5.135056 | IPF11977 | asaprtic proteinase (by homology) |
| 2.199788 | IPF1210 | similar to Saccharomyces cerevisiae Nfu1p involved in homeostasis (by homology) |
| 8.569503 | IPF12209 | similar to Saccharomyces cerevisiae Eco1p involved in sister chromatid cohesion during replication (by homology) |
| 4.776716 | IPF12210 | quinolinate phosphoribosyltransferase (by homology) |
| 3.642419 | IPF1222 | Similar to superoxide dismutase (by homology) |
| 2.017049 | IPF12371 | extracellular alpha-1,4-glucan glucosidase (by homology) |
| 6.397637 | IPF12897 | putative oxidoreductase (by homology) |
| 3.023227 | IPF12963 | ubiquitin-mediated protein degradation (by homology) |
| 2.132334 | IPF13744 | protein involved in regulation of carbon metabolism (by homology) |
| 2.136691 | IPF1401 | similarity to aldehyde dehydrogenase (by homology) |
| 5.755182 | IPF14155 | similar to Saccharomyces cerevisiae Rim4p involved in sporulation (by homology) |
| 8.999195 | IPF14662 | D-xylose reductase (by homology) |
| 3.210271 | IPF1524 | putative multidrug resistance protein (by homology) |
| 4.897046 | IPF15494 | putative ribonuclease (by homology) |
| 2.369552 | IPF15679 | lipid transfer protein (by homology) |
| 4.246947 | IPF15741 | similar to Saccharomyces cerevisiae Cyk3p possibly involved in cytokinesis (by homology) |
| 2.203145 | IPF16300 | putative aldehyde dehydrogenase (by homology) |
| 2.671772 | IPF16795 | glycerate/formate-dehydrogenase (by homology) |
| 2.342162 | IPF1834 | probable syntaxin (by homology) |
| 3.623417 | IPF1839 | putative 1-Acyl dihydroxyacetone phosphate reductase (by homology) |
| 2.26642 | IPF18587 | putative methyltransferase (by homology) |
| 7.423685 | IPF191 | putative permease (by homology) |
| 2.466721 | IPF1960.3f | putative transcriptional activator, 3-prime end |
| 2.375255 | IPF20014 | oxidoreductase by homology |
| 4.592936 | IPF20065 | similar to Saccharomyces cerevisiae Yku70p high-affinity DNA-binding protein (by homology) |
| 3.162736 | IPF2130 | similar to 2-nitropropane dioxygenases |
| 3.136942 | IPF2431 | similar to Saccharomyces cerevisiae Tsa1p thiol-specific antioxidant-like protein (by homology) |
| 2.639101 | IPF2846 | similar to Saccharomyces cerevisiae Mps1p serine/threonine/tyrosine protein kinase (by homology) |
| 2.472021 | IPF3014 | weak similarity to S. cerevisiae DOS2 involved in genome stability |
| 2.141507 | IPF3032 | allantoate permease (by homology) |
| 3.364141 | IPF3094 | 4-nitrophenyl phosphatase (by homology) |
| 2.216177 | IPF3098 | Putative mannosyltransferase (by homology) |
| 2.448422 | IPF32 | similar to Saccharomyces cerevisiae Apg9p integral membrane protein required for Cvt and autophagy transport (by homology) |
| 2.387807 | IPF3227 | similar to Saccharomyces cerevisiae chromatin assembly complex, subunit p50 (by homology) |
| 2.762206 | IPF3282 | hexose transporter (by homology) |
| 6.591028 | IPF3415 | similar to Saccharomyces cerevisiae Yim1p mitochondrial inner membrane protease (by homology) |
| 2.175095 | IPF345 | FH1/FH2 involved in cytokinesis and polarity (by homology) |
| 2.950687 | IPF3485 | aldo/keto reductase (by homology) |
| 2.321794 | IPF3533 | putative GDP/GTP exchange factor (by homology) |
| 2.948443 | IPF3714 | similar to Saccharomyces cerevisiae Cdc12p septin (by homology) |
| 2.34084 | IPF3737 | similar to Saccharomyces cerevisiae Aps1p AP-1 complex subunit, sigma1 subunit (by homology) |
| 7.187521 | IPF3887 | similar to Saccharomyces cerevisiae Syg1p plasma membrane protein of the major facilitator superfamily (by homology) |
| 2.785842 | IPF3968 | similar to Saccharomyces cerevisiae pre-mRNA splicing factor RNA helicase of DEAD box family Prp28p (by homology) |
| 2.800977 | IPF4240 | similar to Saccharomyces cerevisiae Ycg1p condensin G (by homology) |
| 2.331546 | IPF428 | transport protein (by homology) |
| 2.310907 | IPF4477 | similar to Saccharomyces cerevisiae Doa1p involved in ubiquitin-dependent proteolysis (by homology) |
| 4.573695 | IPF4500 | putative GTP-binding protein (by homology) |
| 5.598647 | IPF4588 | putative aldehyde dehydrogenase (by homology) |
| 2.365355 | IPF4866 | similar to Saccharomyces cerevisiae Yuh1p ubiquitin-specific protease |
| 4.858511 | IPF4959 | D-xylulose reductase (by homology) |
| 2.829759 | IPF5234 | X-Pro dipeptidase (by homology) |
| 2.431345 | IPF5673 | similar to Saccharomyces cerevisiae Rex3p RNA exonuclease (by homology) |
| 2.517669 | IPF5915 | phosphatidyl synthase (by homology) |
| 2.661119 | IPF5972 | putative cysteine dioxygenase (by homology) |
| 4.647326 | IPF6037 | Similar to Legionella pneumophila sbpA |
| 3.82221 | IPF6041 | Similar to Legionella pneumophila sbpA |
| 3.011882 | IPF6079 | putative permease (by homology) |
| 2.600616 | IPF6181 | similar to Saccharomyces cerevisiae Fun 26p nucleoside transporter (by homology) |
| 2.550544 | IPF6716 | Phenylacetate 2-hydroxylase (by homology) |
| 4.071026 | IPF7297.3 | similar to Saccharomyces cerevisiae Mlc1p myosin ((Myo2p) light chain, 3-prime end (by homology) |
| 2.803205 | IPF7557 | similar to Saccharomyces cerevisiae Smi1p involved in beta-1,3-glucan synthesis (by homology) |
| 4.80198 | IPF7602 | oxidoreductase (by homology) |
| 2.746997 | IPF763 | putative transcription factor with a Cys4- zinc finger (by homology) |
| 3.572408 | IPF7732 | similar to Saccharomyces cerevisiae Hcm1p transcription factor with fork head domain (by homology) |
| 2.495406 | IPF7778 | putative carboxymethylenebutenolidase (dienelactone hydrolase, DLH) (by homology) |
| 3.768671 | IPF7874 | similar to Saccharomyces cerevisiae Esc4p involved in chromatin silencing (by homology) |
| 2.548296 | IPF7938 | similar to Saccharomyces cerevisiae Stu2p suppressor of cold-sensitive tubulin mutation (by homology) |
| 2.366921 | IPF823 | tRNA(5-methylaminomethyl-2-thiouridylate)-methyltransferase (by homology) |
| 3.379263 | IPF824 | 2-hydroxyhepta-2,4-diene-1,7-dioate isomerase (by homology) |
| 2.075218 | IPF8245 | putative chitinase |
| 2.163326 | IPF836.3 | regulation of G-protein function, 3-prime end (by homology) |
| 2.254484 | IPF8644 | maltase (by homology) |
| 3.919797 | IPF9108 | similar to Saccharomyces cerevisiae Ace2p transcription factor (by homology) |
| 17.22651 | IPF9136.5eoc | potassium transporter, 5-prime end (by homology) |
| 2.275838 | IPF9160 | similar to Saccharomyces cerevisiae Med4ptranscription regulation mediator (by homology) |
| 3.055366 | IPF928 | zinc-finger transcription factor of the Zn(2)-Cys(6) binuclear cluster domain type (by homology) |
| 8.353404 | IPF9490 | amino acid permease (by homology) |
| 2.29057 | IPF9758 | similar to Saccharomyces cerevisiae Dpb3p DNA-directed DNA polymerase epsilon, subunit C (by homology) |
| 3.215648 | IPF9808 | similar to Saccharomyces cerevisiae cse4p with strong similarity to histone H3 (by homology) |
| 2.222414 | IPF9901 | similar to Saccharomyces cerevisiae rad18p DNA repair protein (by homology) |

| Downregulated genes | | |
| --- | --- | --- |
| Ratio | Common | Description |
| 0.458804 | CaAAT22 | aspartate aminotransferase (by homology) |
| 0.321227 | CaABC1 | ubiquinol--cytochrome-c reductase(by homology) |
| 0.394036 | CaACC1 | acetyl-coenzyme-A carboxylase (by homology) |
| 0.441526 | CaADE2 | phosphoribosylaminoimidazole carboxylase |
| 0.239174 | CaAFG1 | ATPase family gene (by homology) |
| 0.160372 | CaAGP2 | amino-acid permease (by homology) |
| 0.482012 | CaALK6 | n-alkane inducible cytochrome P-450 (by homology) |
| 0.468946 | CaALS11.3f | agglutinin-like protein, 3-prime end |
| 0.331316 | CaALS12.3f | agglutinin-like protein, 3-prime end |
| 0.377711 | CaALS4.3f | agglutinin-like protein, 3-prime end |
| 0.320416 | CaALS4.5f | agglutinin-like protein, 5-prime end |
| 0.426458 | CaAMD1 | AMP deaminase (by homology) |
| 0.479291 | CaAPA2 | ATP adenylyltransferase II (by homology) |
| 0.410405 | CaAPM1 | AP-1 complex subunit, mu1 subunit (by homology) |
| 0.459651 | CaARH1 | adrenodoxin reductase and ferredoxin-NADP+ reductase (by homology) |
| 0.330071 | CaASP1 | L-asparaginase (by homology) |
| 0.359846 | CaATE1 | arginyl tRNA transferase (by homology) |
| 0.413903 | CaATH1 | acid trehalase, vacuolar |
| 0.303501 | CaAYR2 | 1-acyl dihydroxyacetone phosphate reductase (by homology) |
| 0.427663 | CaCCC2 | putative copper-transporting ATPase (by homology) |
| 0.320279 | CaCDC19 | pyruvate kinase (by homology) |
| 0.407179 | CaCDC23 | Subunit of anaphase-promoting complex (by homology) |
| 0.437065 | CaCDC34 | Ubiquitin-conjugating enzyme (by homology) |
| 0.39074 | CaCDR3.3eoc | Opaque-specific ABC transporter, 3-prime end |
| 0.456934 | CaCFL3 | ferric reductase-like protein |
| 0.476959 | CaCHA11 | L-serine/L-threonine deaminase (by homology) |
| 0.410422 | CaCHO2 | phosphatidylethanolamine N-methyltransferase (by homology) |
| 0.323996 | CaCirt4b | probable transposase (by homology) |
| 0.231764 | CaCLN2 | G1/S-SPECIFIC CYCLIN CLN2 |
| 0.407424 | CaCNA1 | cyclic nucleotide phosphodiesterase |
| 0.291201 | CaCNS1 | Cyclophilin Seven Suppressor (by homology) |
| 0.388836 | CaCPP1 | probable protein-tyrosine phosphatase |
| 0.304148 | CaCRK1.3f | Protein kinase, 3-prime end |
| 0.397669 | CaCYC3 | cytochrome C heme lyase |
| 0.326381 | CaCYS3 | cystathionine gamma-lyase by homology |
| 0.32725 | CaCZF1 | canal zinc finger protein |
| 0.361483 | CaDAL81 | Transcriptional activator for allantoin and GABA catabolic genes (by homology) |
| 0.389248 | CaDBP2.exon2 | ATP-dependent RNA helicase of DEAD box family, exon 2 (by homology) |
| 0.427849 | CaDOM34 | probable involvement in meiotic and mitotic divisions (by homology) |
| 0.264811 | CaDOT4 | derepression of telomeric silencing (by homology) |
| 0.310524 | CaEBP1 | NADPH dehydrogenase |
| 0.458043 | CaECM3 | Involved in cell wall biogenesis and architecture (by homology). |
| 0.34277 | CaECM7 | cell wall biogenesis and architecture (by homology) |
| 0.412019 | CaELF1 | Elongation-like factor |
| 0.426747 | CaENP1 | Essential nuclear protein (by homology) |
| 0.351876 | CaERC1 | ethionine resistance protein (by homology) |
| 0.434352 | CaERG25 | C-4 sterol methyl oxidase |
| 0.48533 | CaFAA22 | Long-chain-fatty-acid--CoA ligase (by homology) |
| 0.246947 | CaFAA4 | long-chain fatty acid--CoA ligase and synthetase 4 (by homology) |
| 0.496119 | CaFAS2.3f | fatty-acyl-CoA synthase, alpha chain, 3-prime end |
| 0.424267 | CaFEN11 | Fatty acid elongase required for sphingolipid formation (by homology) |
| 0.157513 | CaFET33 | cell surface ferroxidase (by homology) |
| 0.12138 | CaFET34.3eoc | iron transport multicopper oxidase, 3-prime end (by homology) |
| 0.153044 | CaFRE30.3 | Strong similarity to ferric reductase Fre2p, 3-prime end (by homology) |
| 0.312884 | CaFRE5 | ferric reductase transmembrane component (by homology) |
| 0.3786 | CaFRP2 | member of the FRP family of proteins related to Yarrowia lipolytica glyoxylate pathway regulator Gpr1p and Saccharomyces cerevisiae Fun34p |
| 0.433457 | CaFTH2 | iron transporter (by homology) |
| 0.248368 | CaFTR1 | high affinity iron permease |
| 0.32248 | CaGAP2 | general amino acid permease (by homology) |
| 0.261082 | CaGAP5 | General amino acid permease (by homology) |
| 0.467715 | CaGCN20 | Positive effector of Gcn2p (by homology) |
| 0.268515 | CaGDS1 | nam9-1 suppressor (by homology) |
| 0.408925 | CaGLR1 | by similarity to S. cerev. and C. albicans:glutathione reductase |
| 0.347927 | CaGPD2 | Glycerol 3-phosphate dehydrogenase (by homology) |
| 0.399923 | CaGRR1 | Required for glucose repression and for glucose and cation transport (by homology) |
| 0.442215 | CaGRX3 | glutaredoxin-like protein |
| 0.466968 | CaGSH2 | Glutathione synthetase (by homology) |
| 0.251673 | CaGSL21 | 1,3-beta-D-glucan synthase subunit |
| 0.469769 | CaGYP1 | GTPase activating protein (by homology) |
| 0.42449 | CaHCA4 | Can suppress the U14 snoRNA rRNA processing function |
| 0.2328 | CaHK1 | Histidine kinase |
| 0.400658 | CaHOM3 | Aspartokinase (by homology) |
| 0.320083 | CaHRR25 | casein kinase I (by homology) |
| 0.303569 | CaHXT61 | sugar transporter |
| 0.422878 | CaIFM2 | Glycerate-formate-dehydrogenases (by homology) |
| 0.413893 | CaIFN3 | glycerophosphoinositol transporter (by homology) |
| 0.227496 | CaILV3 | dihydroxyacid dehydratase (by homology) |
| 0.434146 | CaIMP4 | Ribonucleoprotein (by homology) |
| 0.150379 | CaKRE2.3f | secretory pathway protein, 3-prime end |
| 0.456507 | CaKTI12 | involved in resistance to K.lactis killer toxin (by homology) |
| 0.42848 | CaLCP5 | Ngg1p interacting protein (by homology) |
| 0.482982 | CaMAK21 | Ribosome biogenesis protein (by homology) |
| 0.380663 | CaMAP1 | methionine aminopeptidase, isoform 1 (by homology) |
| 0.282221 | CaMET1 | siroheme synthase (by homology) |
| 0.378052 | CaMET222 | protein ser/thr phosphatase (by homology) |
| 0.386141 | CaMEX67 | poly(A)+RNA binding protein involved in nuclear mRNA export (by homology) |
| 0.166833 | CaMNN2 | Golgi alpha-1,2-mannosyltransferase (by homology) |
| 0.48958 | CaMNT2 | Alpha-1,2-mannosyltransferase (by homology) |
| 0.415544 | CaMNT3.3eoc | Putative mannosyltransferase, 3-prime end |
| 0.482797 | CaMPP10 | component of the U3 small nucleolar ribonucleoprotein (by homology) |
| 0.291862 | CaMRS7 | suppressor splicing defects (by homology) |
| 0.429051 | CaMSS116 | RNA helicase of the DEAD box family (by homology) |
| 0.415329 | CaMSS51 | involved in maturation of COX1 and COB mRNA (by homology) |
| 0.457159 | CaMSW1 | Mitochondrial tryptophanyl-tRNA synthetase (by homology) |
| 0.370919 | CaMTF1 | RNA polymerase specific factor, mitochondrial (by homology) |
| 0.406439 | CaMTR | neutral amino acid permease-like by homology |
| 0.423228 | CaMTR10 | Involved in nuclear protein import |
| 0.417989 | CaMTR2.3 | mRNA transport protein, 3-prime end (by homology) |
| 0.36615 | CaMUM2 | ubiquitin C-terminal hydrolase (by homology) |
| 0.424675 | CaNAB3 | polyadenylated RNA-binding protein (by homology) |
| 0.402667 | CaNAT2 | N-acetyltransferase for N-terminal methionine (by homology) |
| 0.459335 | CaNCE11 | involved in non-classical protein export pathway (by homology) |
| 0.453953 | CaNHP2 | nucleolar rRNA processing protein (by homology) |
| 0.371364 | CaNIP1 | translation initiation factor subunit |
| 0.327431 | CaNMD5 | putative Nam7p/Upf1p-interacting protein (by homolgy) |
| 0.472963 | CaNMT1 | N-myristoyltransferase |
| 0.425848 | CaNOG1 | Nucleolar G-protein (by homology) |
| 0.367454 | CaNPL6 | Nuclear protein localization factor (by homology) |
| 0.461349 | CaNPR2 | nitrogen permease regulator (by homology) |
| 0.448197 | CaNSR1 | nuclear localization sequence binding protein (by homology) |
| 0.451866 | CaOSM2 | Osmotic growth protein (by homology) |
| 0.448636 | CaPDC11 | Pyruvate decarboxylase (by homology) |
| 0.404403 | CaPEL1 | CDP-diacylglycerol-serine-O-phosphatidyltransfer ase (by homology) |
| 0.453005 | CaPFK1 | 6-phosphofructokinase, alpha subunit |
| 0.085684 | CaPHO84.3eoc | Inorganic phosphate transport protein, 3-prime end (by homology) |
| 0.205314 | CaPHO89 | Na+-coupled phosphate transport (by homology) |
| 0.473973 | CaPIK1 | phosphatidylinositol 4-kinase |
| 0.461137 | CaPLC3 | phosphatidylinositol phospholipase C |
| 0.311589 | CaPPH21 | protein ser/thr phosphatase PP2A-1 (by homology) |
| 0.433705 | CaPPM2 | carboxy methyl transferase; homolog of PPM1 (by homology) |
| 0.422843 | CaPRP12 | involved in early maturation of pre-rRNA (by homology) |
| 0.388704 | CaPRP39.3 | pre-mRNA splicing factor, 3-prime end (by homology) |
| 0.469185 | CaPRS1 | Ribose-phosphate pyrophosphokinase |
| 0.303602 | CaPTC2 | Protein phosphatase type 2C (by homology) |
| 0.471777 | CaPTM1 | Possibly involved in the TCA cycle (by homology) |
| 0.322426 | CaPUF2.exon2 | RNA-binding protein, exon 2 (by homology) |
| 0.119489 | CaQDR1 | putative antibiotic resistance proteins (by homology) |
| 0.420821 | CaRAS1 | GTP-binding protein (by homology) |
| 0.066179 | CaRBT2 | Repressed by TUP1 protein 2; Rbt2p, Ferric reductase (by homology) |
| 0.289123 | CaRBT5 | repressed by TUP1 protein 5 |
| 0.215958 | CARHR2 | DL-glycerol phosphatase |
| 0.413663 | CaRIM2 | mitochondrial carrier protein (by homology) |
| 0.423223 | CaRNR2 | Ribonucleotide reductase (by homology) |
| 0.125161 | CaRNR22 | ribonucleoside-diphosphate reductase (by homology) |
| 0.264486 | CaROD1 | O-dinitrobenzene,calcium and zinc resistance protein (by homology) |
| 0.31288 | CaROT1 | Suppressor of TOR2 mutations (by homology) |
| 0.449668 | CaROX1 | Possible heme-dependent transcriptional repressor (by homology) |
| 0.437561 | CaRPA49 | DNA-directed RNA polymerase A (by homology) |
| 0.417505 | CaRPB4.3f | DNA-directed RNA polymerase II, 32 kDa subunit, 3-prime end (by homology) |
| 0.435123 | CaRPB5 | DNA-directed RNA polymerase I, II, III (by homology) |
| 0.332595 | CaRPB7 | DNA-directed RNA polymerase II,19KD subunit (by homology) |
| 0.483417 | CaRPL16A | ribosomal protein (by homology) |
| 0.461686 | CaRPL23B.3 | ribosomal protein L23.e, 3-prime end (by homology) |
| 0.472356 | CaRPL28.3f | Ribosomal protein, 3-prime end (by homology) |
| 0.454504 | CaRPL5 | ribosomal protein (by homology) |
| 0.433986 | CaRPO41 | Mitochondrial DNA-directed RNA polymerase (by homology) |
| 0.445865 | CaRPS7A | ribosomal protein (by homology) |
| 0.431481 | CaRPS9B | Ribosomal protein |
| 0.334471 | CaRRP9 | U3 small nucleolar ribonucleoprotein-associated protein involved in pre-ribosomal RNA processing (by homology) |
| 0.444047 | CaRSP5 | ubiquitin-protein ligase (by homology) |
| 0.282367 | CaSCH9 | strong similarity to S.pombe sck1 protein kinase (by homology) |
| 0.417227 | CaSDH42 | succinate dehydrogenase membrane anchor subunit for sdh2p (by homology) |
| 0.315928 | CaSEC14 | phosphatidylinositol(PI)/phosphatidylcholine(PC)transfer |
| 0.315676 | CaSEC20 | secretory pathway protein |
| 0.215105 | CaSEF1 | Putative transcription factor1 |
| 0.431509 | CaSFP1 | zinc finger protein (by homology) |
| 0.266939 | CaSIT1 | Ferrioxamine B permease by homology |
| 0.2905 | CaSKN1.3 | Glucan synthase subunit, 3-prime end |
| 0.361321 | CaSLN1 | Two-component signal transducer histidine kinase component |
| 0.377411 | CaSNG4 | Drug transporter (by homology) |
| 0.385344 | CaSNU71 | Associated with U1 snRNP (by homology) |
| 0.159392 | CaSOU2 | Sorbitol utilization protein Sou2p [Candida albicans] |
| 0.488427 | CaSPB1 | Putative methyltransferase by homology |
| 0.362234 | CaSPT7 | transcription factor, member of the histone acetyltransferase SAGA complex (by homology) |
| 0.252423 | CaSQT1 | suppresses dominant-negative mutants of the ribosomal protein QSR1 (by homology) |
| 0.463583 | CaSRP40 | RNA I and II supressor (by homology) |
| 0.372798 | CaSSK2 | MAP kinase kinase kinase of the high osmolarity signal transduction pathway (by homology) |
| 0.36205 | CaSSN8 | C-type cyclin associated with the Ssn3p cyclin-dependent kinase (by homology) |
| 0.429012 | CaSUN42 | Putative cell wall beta-glucosidase (by homology) |
| 0.461841 | CaSUP45 | Translational release factor (by homology) |
| 0.254056 | CaSUV3 | ATP-dependent RNA helicase, mitochondrial (by homology) |
| 0.468453 | CaTOM37 | Mitochondrial outer membrane import receptor subunit (by homology) |
| 0.310858 | CaTOM72 | mitochondrial import receptor (by homology) |
| 0.430249 | CaTRM3 | 2 -O-ribose methyltransferase (by homology) |
| 0.466937 | CaTYS1 | tyrosyl-tRNA synthetase by homology |
| 0.473662 | CaUAP1 | UDP-N-acetylglucosamine pyrophosphorylase |
| 0.436347 | CaURA1 | dihydroorotate dehydrogenase |
| 0.19016 | CaURA3 | orotidine-5 -monophosphate decarboxylase [Candida albicans] |
| 0.399445 | CaUTR1 | Associated with ferric reductase activity (by homology) |
| 0.411527 | CaVPS24 | endosomal Vps protein complex subunit |
| 0.451148 | CaVPS27 | Vacuolar protein sorting (by homology) |
| 0.338953 | CaYHB2 | flavohemoprotein (by homology) |
| 0.372922 | CaZorro2a.3f | Reverse transcriptase, 3-prime end |
| 0.263646 | CaZRC1 | Zinc and cadmium resistance protein (by homology) |
| 0.404392 | CaZRT1 | high-affinity zinc transport protein (by homology) |
| 0.454942 | IPF10045 | similar to Saccharomyces cerevisiae Taf61p TFIID and SAGA subunit (by homology) |
| 0.324445 | IPF10325 | molybdopterin-converting factor by homology |
| 0.393731 | IPF1067 | Putative glutamate decarboxylase (by homology) |
| 0.395744 | IPF11105 | probable quinone oxidoreductase |
| 0.406411 | IPF11548 | serine/threonine protein kinase (by homology) |
| 0.355903 | IPF11815 | similar to Saccharomyces cerevisiae Lys14 triacyglycerol lipase (by homology) |
| 0.440261 | IPF12101 | mycelial surface antigen precursor (by homology to Candida gene CSA1) |
| 0.13293 | IPF1218 | Similar to superoxide dismutase (by homology) |
| 0.424119 | IPF12201 | Na+-nucleoside cotransporter (by homology) |
| 0.35974 | IPF1334 | Conserved hypothetical protein |
| 0.44493 | IPF13448 | similar to Saccharomyces Ptp1p protein tyrosine phosphatase (by homology) |
| 0.204107 | IPF13825 | similarity to serine/threonine protein kinases (by homology) |
| 0.462176 | IPF13836 | probable heat shock protein (by homology) |
| 0.361717 | IPF14455 | similar to Saccharomyces cerevisiae Rrn7p polymerase I specific transcription initiation factor (by homology) |
| 0.335028 | IPF14763 | delta-12 fatty acid desaturase (by homology) |
| 0.369397 | IPF14782 | beta-transducin (by homology) |
| 0.32233 | IPF1500 | similar to Saccharomyces cerevisiae Nca2p regulating expression of mitochondrial ATP synthase (by homology) |
| 0.367405 | IPF1505 | similar to saccharomyces cerevisiae Kre6p glucan synthase subunit (by homology) |
| 0.36224 | IPF15646 | putative ATP-dependent RNA helicase (by homology) |
| 0.429638 | IPF15927.3f | similar to Saccharomyces cerevisiae Tfc3p transcription initiation factor TFIIIC, 3-prime end (by homology) |
| 0.282199 | IPF15927.5f | similar to Saccharomyces cerevisiae Tfc3p transcription initiation factor TFIIIC, 5-prime end (by homology) |
| 0.288505 | IPF17676 | similar to Saccharomyces cerevisiae Cst13p involved in chromosome stability (by homology) |
| 0.291445 | IPF18125 | similar to glutenin and glutamine-rich proteins |
| 0.412101 | IPF19614 | putative transcription factor similar to positive activator of the proline utilisation pathway (by homology) |
| 0.425023 | IPF19724 | similar to Saccharomyces cerevisiae Tbf1ptelomere repeat-binding factor 1 |
| 0.409119 | IPF20031 | similar to Saccharomyces cerevisiae Psr2p plasma membrane phosphatase required for sodium stress response (by homology) |
| 0.484312 | IPF2096 | putative acyltransferase (by homology) |
| 0.354511 | IPF3366 | Mitochondrial ribosomal protein (by homology) |
| 0.340364 | IPF3375 | similar to Saccharomyces cerevisiae Rer2p cis-prenyltransferase, a key enzyme in dolichol synthesis (by homology) |
| 0.36844 | IPF3594 | triglyceride lipase (by homology) |
| 0.402704 | IPF4087 | similar to Saccharomyces cerevisiae Pak1p protein kinase suppressing mutations in DNA polymerase alpha (by homology) |
| 0.398716 | IPF4089 | secretory aspartyl proteinase |
| 0.267734 | IPF4514 | putative alpha-1,3-mannosyltransferase (by homology) |
| 0.467876 | IPF4632 | similar to Saccharomyces cerevisiae Vps53p subunit of VP52-54 complex, required for protein sorting at the yeast late Golgi (by homology) |
| 0.361053 | IPF4814 | similar to Saccharomyces cerevisiae Mrp20p ribosomal protein of the large subunit, mitochondrial (by homology) |
| 0.466874 | IPF5052 | RNA-binding protein (by homology) |
| 0.132335 | IPF5185 | putative cell wall protein (by homology) |
| 0.176253 | IPF5268.exon2 | choline monooxygenase, exon 2 (by homology) |
| 0.179271 | IPF5291 | UDP-glucose 4-epimerase (by homology) |
| 0.474496 | IPF5533 | ABC transporter (by homology) |
| 0.248857 | IPF5981 | similar to Saccharomyces cerevisiae Gin3p (by homology) |
| 0.385946 | IPF6054 | unnown function |
| 0.312139 | IPF6067 | putative transcription factor (by homology) |
| 0.475083 | IPF6149 | similar to Saccharomyces cerevisiae Ubp8p deubiquinating enzyme (by homology) |
| 0.358148 | IPF7224 | putative telomere elongation protein (by homology) |
| 0.281961 | IPF7227 | putative fatty acid desaturase (by homology) |
| 0.462761 | IPF726 | rna binding protein (by homology) |
| 0.41085 | IPF7530 | ATP-binding-cassette protein (by homology to A.gambiae) |
| 0.439702 | IPF7616 | putative homoserine O-acetyltransferase (by homology) |
| 0.448116 | IPF7711 | related to Neurospora crassa AP-1-like transcription factor (by homology) |
| 0.232045 | IPF7817 | putative NADH-dependent flavin oxidoreductase (by homology) |
| 0.45327 | IPF7841 | similar to Saccharomyces cerevisiae Ndc1p nuclear envelope protein (by homology) |
| 0.444599 | IPF7858 | similar to Saccharomyces cerevisiae Prp24p U4/U6 splicing factor (by homology) |
| 0.408107 | IPF798 | transcriptional regulator (by homology) |
| 0.400567 | IPF8048 | probable succinate-semialdehyde dehydrogenase (by homology) |
| 0.396208 | IPF8267 | P-type ATPase |
| 0.352162 | IPF8307 | putative permease (by homology) |
| 0.46384 | IPF8326 | similar to Saccharomyces cerevisiae Rnc1p endo-exonuclease (by homology) |
| 0.284411 | IPF8405 | similar to Saccharomyces cerevisiae Nce103p involved in non-classical protein export pathway (by homology) |
| 0.432986 | IPF8576 | similar to Saccharomyces cerevisiae Ris1p DNA helicase (by homology) |
| 0.387075 | IPF8796 | putative GPI-anchhored protein related to Phr1, Phr2 and Phr3 (by homology) |
| 0.467294 | IPF9017 | similar to Saccharomyces cerevisiae She4p required for mother cell-specific gene expression (by homology) |
| 0.422444 | IPF9096 | probable mannosidase (by homology) |
| 0.405787 | IPF9099 | similar to Saccharomyces cerevisiae Aos1p activating enzyme for Smt3 (by homology) |
| 0.23174 | IPF9385 | similar to Saccharomyces cerevisiae Pho2p homeobox-domain containing transcription factor (by homology) |

Table S2 Upregulated genes classified based on ontology.

| Category | Genes in Category | % of Genes in Category | Genes in List in Category | % of Genes in List in Category | p-Value |
| --- | --- | --- | --- | --- | --- |
| GO:18456: aryl-alcohol dehydrogenase activity | 9 | 0.198 | 9 | 2.564 | 8.74E-11 |
| GO:16491: oxidoreductase activity | 420 | 9.225 | 67 | 19.09 | 1.75E-09 |
| GO:51187: cofactor catabolism | 33 | 0.666 | 15 | 4.132 | 2.15E-09 |
| GO:9109: coenzyme catabolism | 33 | 0.666 | 15 | 4.132 | 2.15E-09 |
| GO:46356: acetyl-CoA catabolism | 33 | 0.666 | 15 | 4.132 | 2.15E-09 |
| GO:6099: tricarboxylic acid cycle | 33 | 0.666 | 15 | 4.132 | 2.15E-09 |
| GO:6084: acetyl-CoA metabolism | 33 | 0.666 | 15 | 4.132 | 2.15E-09 |
| GO:6732: coenzyme metabolism | 67 | 1.351 | 21 | 5.785 | 4.62E-09 |
| GO:30312: external encapsulating structure | 130 | 2.736 | 30 | 8 | 4.50E-08 |
| GO:5618: cell wall | 130 | 2.736 | 30 | 8 | 4.50E-08 |
| GO:51186: cofactor metabolism | 76 | 1.533 | 21 | 5.785 | 5.58E-08 |
| GO:6092: main pathways of carbohydrate metabolism | 83 | 1.674 | 22 | 6.061 | 6.11E-08 |
| GO:15980: energy derivation by oxidation of organic compounds | 175 | 3.53 | 34 | 9.366 | 8.09E-08 |
| GO:5975: carbohydrate metabolism | 86 | 1.735 | 22 | 6.061 | 1.22E-07 |
| GO:44262: cellular carbohydrate metabolism | 86 | 1.735 | 22 | 6.061 | 1.22E-07 |
| GO:6091: generation of precursor metabolites and energy | 207 | 4.175 | 37 | 10.19 | 2.02E-07 |
| GO:45333: cellular respiration | 113 | 2.279 | 25 | 6.887 | 3.55E-07 |
| GO:9060: aerobic respiration | 113 | 2.279 | 25 | 6.887 | 3.55E-07 |
| GO:9986: cell surface | 261 | 5.494 | 44 | 11.73 | 6.73E-07 |
| GO:16614: oxidoreductase activity, acting on CH-OH group of donors | 84 | 1.845 | 21 | 5.983 | 8.39E-07 |
| GO:16616: oxidoreductase activity, acting on the CH-OH group of donors, NAD or NADP as acceptor | 72 | 1.581 | 19 | 5.413 | 1.16E-06 |
| GO:9277: cell wall (sensu Fungi) | 101 | 2.126 | 23 | 6.133 | 2.27E-06 |
| GO:5657: replication fork | 65 | 1.368 | 17 | 4.533 | 6.84E-06 |
| GO:30472: mitotic spindle organization and biogenesis in nucleus | 19 | 0.383 | 8 | 2.204 | 2.82E-05 |
| GO:45836: positive regulation of meiosis | 4 | 0.0807 | 4 | 1.102 | 2.83E-05 |
| GO:4582: dolichyl-phosphate beta-D-mannosyltransferase activity | 4 | 0.0879 | 4 | 1.14 | 3.48E-05 |
| GO:4514: nicotinate-nucleotide diphosphorylase (carboxylating) activity | 4 | 0.0879 | 4 | 1.14 | 3.48E-05 |
| GO:16903: oxidoreductase activity, acting on the aldehyde or oxo group of donors | 35 | 0.769 | 11 | 3.134 | 3.77E-05 |
| GO:45026: plasma membrane fusion | 7 | 0.141 | 5 | 1.377 | 3.80E-05 |
| GO:30471: spindle pole body and microtubule cycle (sensu Fungi) | 26 | 0.524 | 9 | 2.479 | 5.58E-05 |
| GO:6260: DNA replication | 141 | 2.844 | 24 | 6.612 | 7.02E-05 |
| GO:30446: hyphal cell wall | 56 | 1.179 | 14 | 3.733 | 7.70E-05 |
| GO:4033: aldo-keto reductase activity | 8 | 0.176 | 5 | 1.425 | 0.00012 |
| GO:16576: histone dephosphorylation | 5 | 0.101 | 4 | 1.102 | 0.00013 |
| GO:8863: formate dehydrogenase activity | 5 | 0.11 | 4 | 1.14 | 0.00016 |
| GO:4035: alkaline phosphatase activity | 5 | 0.11 | 4 | 1.14 | 0.00016 |
| GO:279: M phase | 306 | 6.172 | 40 | 11.02 | 0.00018 |
| GO:6118: electron transport | 24 | 0.484 | 8 | 2.204 | 0.0002 |
| GO:6261: DNA-dependent DNA replication | 108 | 2.178 | 19 | 5.234 | 0.00026 |
| GO:6281: DNA repair | 164 | 3.308 | 25 | 6.887 | 0.00031 |
| GO:51084: posttranslational protein folding | 20 | 0.403 | 7 | 1.928 | 0.00036 |
| GO:42026: protein refolding | 20 | 0.403 | 7 | 1.928 | 0.00036 |
| GO:6734: NADH metabolism | 6 | 0.121 | 4 | 1.102 | 0.00038 |
| GO:6735: NADH regeneration | 6 | 0.121 | 4 | 1.102 | 0.00038 |
| GO:15757: galactose transport | 3 | 0.0605 | 3 | 0.826 | 0.00039 |
| GO:9056: catabolism | 86 | 1.735 | 16 | 4.408 | 0.00041 |
| GO:44248: cellular catabolism | 86 | 1.735 | 16 | 4.408 | 0.00041 |
| GO:6271: DNA strand elongation | 33 | 0.666 | 9 | 2.479 | 0.00044 |
| GO:50625: 2-hydroxy-1,4-benzoquinone reductase activity | 3 | 0.0659 | 3 | 0.855 | 0.00046 |
| GO:4591: oxoglutarate dehydrogenase (succinyl-transferring) activity | 3 | 0.0659 | 3 | 0.855 | 0.00046 |
| GO:4335: galactokinase activity | 3 | 0.0659 | 3 | 0.855 | 0.00046 |
| GO:3869: 4-nitrophenylphosphatase activity | 3 | 0.0659 | 3 | 0.855 | 0.00046 |
| GO:4852: uroporphyrinogen-III synthase activity | 3 | 0.0659 | 3 | 0.855 | 0.00046 |
| GO:4610: phosphoacetylglucosamine mutase activity | 3 | 0.0659 | 3 | 0.855 | 0.00046 |
| GO:9003: signal peptidase activity | 6 | 0.132 | 4 | 1.14 | 0.00046 |
| GO:6470: protein amino acid dephosphorylation | 21 | 0.424 | 7 | 1.928 | 0.0005 |
| GO:5787: signal peptidase complex | 6 | 0.126 | 4 | 1.067 | 0.0005 |
| GO:3824: catalytic activity | 2726 | 59.87 | 239 | 68.09 | 0.00058 |
| GO:16620: oxidoreductase activity, acting on the aldehyde or oxo group of donors, NAD or NADP as acceptor | 27 | 0.593 | 8 | 2.279 | 0.00069 |
| GO:7127: meiosis I | 43 | 0.867 | 10 | 2.755 | 0.00084 |
| GO:51327: M phase of meiotic cell cycle | 169 | 3.409 | 24 | 6.612 | 0.00115 |
| GO:7126: meiosis | 169 | 3.409 | 24 | 6.612 | 0.00115 |
| GO:51321: meiotic cell cycle | 169 | 3.409 | 24 | 6.612 | 0.00115 |
| GO:16311: dephosphorylation | 24 | 0.484 | 7 | 1.928 | 0.00124 |
| GO:5694: chromosome | 158 | 3.326 | 24 | 6.4 | 0.00124 |
| GO:42773: ATP synthesis coupled electron transport | 18 | 0.363 | 6 | 1.653 | 0.00129 |
| GO:42775: ATP synthesis coupled electron transport (sensu Eukaryota) | 18 | 0.363 | 6 | 1.653 | 0.00129 |
| GO:16679: oxidoreductase activity, acting on diphenols and related substances as donors | 12 | 0.264 | 5 | 1.425 | 0.00133 |
| GO:5940: septin ring | 17 | 0.358 | 6 | 1.6 | 0.00136 |
| GO:7049: cell cycle | 452 | 9.117 | 50 | 13.77 | 0.00158 |
| GO:6273: lagging strand elongation | 25 | 0.504 | 7 | 1.928 | 0.00161 |
| GO:16682: oxidoreductase activity, acting on diphenols and related substances as donors, oxygen as acceptor | 4 | 0.0879 | 3 | 0.855 | 0.00171 |
| GO:9916: alternative oxidase activity | 4 | 0.0879 | 3 | 0.855 | 0.00171 |
| GO:42132: fructose-bisphosphatase activity | 4 | 0.0879 | 3 | 0.855 | 0.00171 |
| GO:6974: response to DNA damage stimulus | 184 | 3.711 | 25 | 6.887 | 0.00173 |
| GO:6289: nucleotide-excision repair | 47 | 0.948 | 10 | 2.755 | 0.00174 |
| GO:6268: DNA unwinding during replication | 19 | 0.383 | 6 | 1.653 | 0.00177 |
| GO:3677: DNA binding | 415 | 9.115 | 48 | 13.68 | 0.0022 |
| GO:9719: response to endogenous stimulus | 199 | 4.014 | 26 | 7.163 | 0.00247 |
| GO:6121: mitochondrial electron transport, succinate to ubiquinone | 9 | 0.182 | 4 | 1.102 | 0.00265 |
| GO:15719: allantoate transport | 9 | 0.182 | 4 | 1.102 | 0.00265 |
| GO:9636: response to toxin | 9 | 0.182 | 4 | 1.102 | 0.00265 |
| GO:16684: oxidoreductase activity, acting on peroxide as acceptor | 20 | 0.439 | 6 | 1.709 | 0.00308 |
| GO:4601: peroxidase activity | 20 | 0.439 | 6 | 1.709 | 0.00308 |
| GO:6769: nicotinamide metabolism | 15 | 0.303 | 5 | 1.377 | 0.00332 |
| GO:6950: response to stress | 446 | 8.996 | 48 | 13.22 | 0.00347 |
| GO:7131: meiotic recombination | 36 | 0.726 | 8 | 2.204 | 0.00375 |
| GO:6272: leading strand elongation | 22 | 0.444 | 6 | 1.653 | 0.00403 |
| GO:15238: drug transporter activity | 21 | 0.461 | 6 | 1.709 | 0.00403 |
| GO:15239: multidrug transporter activity | 21 | 0.461 | 6 | 1.709 | 0.00403 |
| GO:42995: cell projection | 34 | 0.716 | 8 | 2.133 | 0.00408 |
| GO:5937: mating projection | 34 | 0.716 | 8 | 2.133 | 0.00408 |
| GO:6284: base-excision repair | 10 | 0.202 | 4 | 1.102 | 0.00416 |
| GO:5662: DNA replication factor A complex | 5 | 0.105 | 3 | 0.8 | 0.00433 |
| GO:7059: chromosome segregation | 136 | 2.743 | 19 | 5.234 | 0.00442 |
| GO:16798: hydrolase activity, acting on glycosyl bonds | 59 | 1.296 | 11 | 3.134 | 0.00471 |
| GO:6310: DNA recombination | 80 | 1.614 | 13 | 3.581 | 0.0049 |
| GO:19825: oxygen binding | 10 | 0.22 | 4 | 1.14 | 0.00502 |
| GO:6266: DNA ligation | 2 | 0.0403 | 2 | 0.551 | 0.00535 |
| GO:46292: formaldehyde metabolism | 2 | 0.0403 | 2 | 0.551 | 0.00535 |
| GO:19649: formaldehyde assimilation | 2 | 0.0403 | 2 | 0.551 | 0.00535 |
| GO:6730: one-carbon compound metabolism | 2 | 0.0403 | 2 | 0.551 | 0.00535 |
| GO:15764: N-acetylglucosamine transport | 2 | 0.0403 | 2 | 0.551 | 0.00535 |
| GO:15002: heme-copper terminal oxidase activity | 2 | 0.0439 | 2 | 0.57 | 0.00593 |
| GO:4129: cytochrome-c oxidase activity | 2 | 0.0439 | 2 | 0.57 | 0.00593 |
| GO:4032: aldehyde reductase activity | 2 | 0.0439 | 2 | 0.57 | 0.00593 |
| GO:16675: oxidoreductase activity, acting on heme group of donors | 2 | 0.0439 | 2 | 0.57 | 0.00593 |
| GO:16676: oxidoreductase activity, acting on heme group of donors, oxygen as acceptor | 2 | 0.0439 | 2 | 0.57 | 0.00593 |
| GO:4327: formaldehyde dehydrogenase (glutathione) activity | 2 | 0.0439 | 2 | 0.57 | 0.00593 |
| GO:42083: 5,10-methylenetetrahydrofolate-dependent methyltransferase activity | 2 | 0.0439 | 2 | 0.57 | 0.00593 |
| GO:4799: thymidylate synthase activity | 2 | 0.0439 | 2 | 0.57 | 0.00593 |
| GO:4772: sterol O-acyltransferase activity | 2 | 0.0439 | 2 | 0.57 | 0.00593 |
| GO:16751: S-succinyltransferase activity | 2 | 0.0439 | 2 | 0.57 | 0.00593 |
| GO:4149: dihydrolipoyllysine-residue succinyltransferase activity | 2 | 0.0439 | 2 | 0.57 | 0.00593 |
| GO:285: 1-phosphatidylinositol-3-phosphate 5-kinase activity | 2 | 0.0439 | 2 | 0.57 | 0.00593 |
| GO:3964: RNA-directed DNA polymerase activity | 2 | 0.0439 | 2 | 0.57 | 0.00593 |
| GO:3720: telomerase activity | 2 | 0.0439 | 2 | 0.57 | 0.00593 |
| GO:3721: telomeric template RNA reverse transcriptase activity | 2 | 0.0439 | 2 | 0.57 | 0.00593 |
| GO:4244: mitochondrial inner membrane peptidase activity | 2 | 0.0439 | 2 | 0.57 | 0.00593 |
| GO:4037: allantoicase activity | 2 | 0.0439 | 2 | 0.57 | 0.00593 |
| GO:4351: glutamate decarboxylase activity | 2 | 0.0439 | 2 | 0.57 | 0.00593 |
| GO:45118: azole transporter activity | 2 | 0.0439 | 2 | 0.57 | 0.00593 |
| GO:15244: fluconazole transporter activity | 2 | 0.0439 | 2 | 0.57 | 0.00593 |
| GO:333: telomerase catalytic core complex | 2 | 0.0421 | 2 | 0.533 | 0.00621 |
| GO:7119: budding cell isotropic bud growth | 6 | 0.121 | 3 | 0.826 | 0.00659 |
| GO:8272: sulfate transport | 6 | 0.121 | 3 | 0.826 | 0.00659 |
| GO:917: barrier septum formation | 6 | 0.121 | 3 | 0.826 | 0.00659 |
| GO:7157: heterophilic cell adhesion | 6 | 0.121 | 3 | 0.826 | 0.00659 |
| GO:7052: mitotic spindle organization and biogenesis | 56 | 1.129 | 10 | 2.755 | 0.00665 |
| GO:6259: DNA metabolism | 497 | 10.02 | 51 | 14.05 | 0.00686 |
| GO:104: succinate dehydrogenase activity | 11 | 0.242 | 4 | 1.14 | 0.00741 |
| GO:15630: microtubule cytoskeleton | 114 | 2.399 | 17 | 4.533 | 0.00748 |
| GO:3777: microtubule motor activity | 17 | 0.373 | 5 | 1.425 | 0.00754 |
| GO:16624: oxidoreductase activity, acting on the aldehyde or oxo group of donors, disulfide as acceptor | 6 | 0.132 | 3 | 0.855 | 0.00762 |
| GO:19362: pyridine nucleotide metabolism | 18 | 0.363 | 5 | 1.377 | 0.0079 |
| GO:6766: vitamin metabolism | 18 | 0.363 | 5 | 1.377 | 0.0079 |
| GO:6767: water-soluble vitamin metabolism | 18 | 0.363 | 5 | 1.377 | 0.0079 |
| GO:6733: oxidoreduction coenzyme metabolism | 18 | 0.363 | 5 | 1.377 | 0.0079 |
| GO:16051: carbohydrate biosynthesis | 25 | 0.504 | 6 | 1.653 | 0.00793 |
| GO:46364: monosaccharide biosynthesis | 25 | 0.504 | 6 | 1.653 | 0.00793 |
| GO:19319: hexose biosynthesis | 25 | 0.504 | 6 | 1.653 | 0.00793 |
| GO:6094: gluconeogenesis | 25 | 0.504 | 6 | 1.653 | 0.00793 |
| GO:46165: alcohol biosynthesis | 25 | 0.504 | 6 | 1.653 | 0.00793 |
| GO:6090: pyruvate metabolism | 25 | 0.504 | 6 | 1.653 | 0.00793 |
| GO:16209: antioxidant activity | 24 | 0.527 | 6 | 1.709 | 0.00821 |
| GO:910: cytokinesis | 33 | 0.666 | 7 | 1.928 | 0.00861 |
| GO:6465: signal peptide processing | 12 | 0.242 | 4 | 1.102 | 0.00872 |
| GO:6518: peptide metabolism | 12 | 0.242 | 4 | 1.102 | 0.00872 |
| GO:40001: establishment of mitotic spindle localization | 12 | 0.242 | 4 | 1.102 | 0.00872 |
| GO:132: establishment of mitotic spindle orientation | 12 | 0.242 | 4 | 1.102 | 0.00872 |
| GO:51293: establishment of spindle localization | 12 | 0.242 | 4 | 1.102 | 0.00872 |
| GO:51294: establishment of spindle orientation | 12 | 0.242 | 4 | 1.102 | 0.00872 |
| GO:51653: spindle localization | 12 | 0.242 | 4 | 1.102 | 0.00872 |
| GO:5819: spindle | 89 | 1.873 | 14 | 3.733 | 0.00922 |
| GO:8194: UDP-glycosyltransferase activity | 32 | 0.703 | 7 | 1.994 | 0.0095 |
| GO:5199: structural constituent of cell wall | 18 | 0.395 | 5 | 1.425 | 0.00979 |
| GO:3690: double-stranded DNA binding | 25 | 0.549 | 6 | 1.709 | 0.0101 |
| GO:6119: oxidative phosphorylation | 34 | 0.686 | 7 | 1.928 | 0.0102 |
| GO:16859: cis-trans isomerase activity | 12 | 0.264 | 4 | 1.14 | 0.0104 |
| GO:3755: peptidyl-prolyl cis-trans isomerase activity | 12 | 0.264 | 4 | 1.14 | 0.0104 |
| GO:6301: postreplication repair | 13 | 0.262 | 4 | 1.102 | 0.0119 |
| GO:7051: spindle organization and biogenesis | 61 | 1.23 | 10 | 2.755 | 0.0122 |
| GO:87: M phase of mitotic cell cycle | 160 | 3.227 | 20 | 5.51 | 0.0122 |
| GO:3997: acyl-CoA oxidase activity | 7 | 0.154 | 3 | 0.855 | 0.0126 |
| GO:5783: endoplasmic reticulum | 348 | 7.325 | 39 | 10.4 | 0.0142 |
| GO:5625: soluble fraction | 171 | 3.599 | 22 | 5.867 | 0.0145 |
| GO:51640: organelle localization | 45 | 0.908 | 8 | 2.204 | 0.015 |
| GO:45117: azole transport | 3 | 0.0605 | 2 | 0.551 | 0.0153 |
| GO:15903: fluconazole transport | 3 | 0.0605 | 2 | 0.551 | 0.0153 |
| GO:6457: protein folding | 82 | 1.654 | 12 | 3.306 | 0.0153 |
| GO:16639: oxidoreductase activity, acting on the CH-NH2 group of donors, NAD or NADP as acceptor | 3 | 0.0659 | 2 | 0.57 | 0.0169 |
| GO:4808: tRNA (5-methylaminomethyl-2-thiouridylate)-methyltransferase activity | 3 | 0.0659 | 2 | 0.57 | 0.0169 |
| GO:16417: S-acyltransferase activity | 3 | 0.0659 | 2 | 0.57 | 0.0169 |
| GO:30523: dihydrolipoamide S-acyltransferase activity | 3 | 0.0659 | 2 | 0.57 | 0.0169 |
| GO:4581: dolichyl-phosphate beta-glucosyltransferase activity | 3 | 0.0659 | 2 | 0.57 | 0.0169 |
| GO:4076: biotin synthase activity | 3 | 0.0659 | 2 | 0.57 | 0.0169 |
| GO:4611: phosphoenolpyruvate carboxykinase activity | 3 | 0.0659 | 2 | 0.57 | 0.0169 |
| GO:4612: phosphoenolpyruvate carboxykinase (ATP) activity | 3 | 0.0659 | 2 | 0.57 | 0.0169 |
| GO:3994: aconitate hydratase activity | 3 | 0.0659 | 2 | 0.57 | 0.0169 |
| GO:15559: multidrug efflux pump activity | 3 | 0.0659 | 2 | 0.57 | 0.0169 |
| GO:7067: mitosis | 155 | 3.126 | 19 | 5.234 | 0.0174 |
| GO:5874: microtubule | 43 | 0.905 | 8 | 2.133 | 0.0175 |
| GO:5200: structural constituent of cytoskeleton | 80 | 1.757 | 12 | 3.419 | 0.0184 |
| GO:19203: carbohydrate phosphatase activity | 8 | 0.176 | 3 | 0.855 | 0.019 |
| GO:4568: chitinase activity | 8 | 0.176 | 3 | 0.855 | 0.019 |
| GO:7068: negative regulation of transcription, mitotic | 15 | 0.303 | 4 | 1.102 | 0.0202 |
| GO:45896: regulation of transcription, mitotic | 15 | 0.303 | 4 | 1.102 | 0.0202 |
| GO:30004: monovalent inorganic cation homeostasis | 31 | 0.625 | 6 | 1.653 | 0.0227 |
| GO:30641: hydrogen ion homeostasis | 31 | 0.625 | 6 | 1.653 | 0.0227 |
| GO:51453: regulation of cellular pH | 31 | 0.625 | 6 | 1.653 | 0.0227 |
| GO:51452: cellular pH reduction | 31 | 0.625 | 6 | 1.653 | 0.0227 |
| GO:7035: vacuolar acidification | 31 | 0.625 | 6 | 1.653 | 0.0227 |
| GO:45851: pH reduction | 31 | 0.625 | 6 | 1.653 | 0.0227 |
| GO:30427: site of polarized growth | 37 | 0.779 | 7 | 1.867 | 0.0235 |
| GO:19725: cell homeostasis | 58 | 1.17 | 9 | 2.479 | 0.0237 |
| GO:16868: intramolecular transferase activity, phosphotransferases | 15 | 0.329 | 4 | 1.14 | 0.024 |
| GO:16655: oxidoreductase activity, acting on NADH or NADPH, quinone or similar compound as acceptor | 9 | 0.198 | 3 | 0.855 | 0.0269 |
| GO:8379: thioredoxin peroxidase activity | 9 | 0.198 | 3 | 0.855 | 0.0269 |
| GO:3893: epsilon DNA polymerase activity | 9 | 0.198 | 3 | 0.855 | 0.0269 |
| GO:30894: replisome | 38 | 0.8 | 7 | 1.867 | 0.0269 |
| GO:15849: organic acid transport | 69 | 1.392 | 10 | 2.755 | 0.0273 |
| GO:46942: carboxylic acid transport | 69 | 1.392 | 10 | 2.755 | 0.0273 |
| GO:5828: kinetochore microtubule | 9 | 0.189 | 3 | 0.8 | 0.0286 |
| GO:723: telomere maintenance | 4 | 0.0807 | 2 | 0.551 | 0.029 |
| GO:16233: telomere capping | 4 | 0.0807 | 2 | 0.551 | 0.029 |
| GO:7034: vacuolar transport | 70 | 1.412 | 10 | 2.755 | 0.0299 |
| GO:6873: cell ion homeostasis | 33 | 0.666 | 6 | 1.653 | 0.0303 |
| GO:30003: cation homeostasis | 33 | 0.666 | 6 | 1.653 | 0.0303 |
| GO:6885: regulation of pH | 33 | 0.666 | 6 | 1.653 | 0.0303 |
| GO:4735: pyrroline-5-carboxylate reductase activity | 4 | 0.0879 | 2 | 0.57 | 0.032 |
| GO:16748: succinyltransferase activity | 4 | 0.0879 | 2 | 0.57 | 0.032 |
| GO:16307: phosphatidylinositol phosphate kinase activity | 4 | 0.0879 | 2 | 0.57 | 0.032 |
| GO:4038: allantoinase activity | 4 | 0.0879 | 2 | 0.57 | 0.032 |
| GO:3909: DNA ligase activity | 4 | 0.0879 | 2 | 0.57 | 0.032 |
| GO:3910: DNA ligase (ATP) activity | 4 | 0.0879 | 2 | 0.57 | 0.032 |
| GO:5244: voltage-gated ion channel activity | 4 | 0.0879 | 2 | 0.57 | 0.032 |
| GO:5247: voltage-gated chloride channel activity | 4 | 0.0879 | 2 | 0.57 | 0.032 |
| GO:5253: anion channel activity | 4 | 0.0879 | 2 | 0.57 | 0.032 |
| GO:5254: chloride channel activity | 4 | 0.0879 | 2 | 0.57 | 0.032 |
| GO:8509: anion transporter activity | 4 | 0.0879 | 2 | 0.57 | 0.032 |
| GO:3700: transcription factor activity | 287 | 6.304 | 31 | 8.832 | 0.032 |
| GO:5697: telomerase holoenzyme complex | 4 | 0.0842 | 2 | 0.533 | 0.0335 |
| GO:9117: nucleotide metabolism | 34 | 0.686 | 6 | 1.653 | 0.0346 |
| GO:16634: oxidoreductase activity, acting on the CH-CH group of donors, oxygen as acceptor | 10 | 0.22 | 3 | 0.855 | 0.0363 |
| GO:4042: amino-acid N-acetyltransferase activity | 10 | 0.22 | 3 | 0.855 | 0.0363 |
| GO:51082: unfolded protein binding | 10 | 0.22 | 3 | 0.855 | 0.0363 |
| GO:30445: yeast-form cell wall | 58 | 1.221 | 9 | 2.4 | 0.0363 |
| GO:16831: carboxy-lyase activity | 33 | 0.725 | 6 | 1.709 | 0.0377 |
| GO:747: conjugation with cellular fusion | 83 | 1.674 | 11 | 3.03 | 0.0382 |
| GO:3774: motor activity | 25 | 0.549 | 5 | 1.425 | 0.039 |
| GO:35251: UDP-glucosyltransferase activity | 25 | 0.549 | 5 | 1.425 | 0.039 |
| GO:3887: DNA-directed DNA polymerase activity | 25 | 0.549 | 5 | 1.425 | 0.039 |
| GO:5876: spindle microtubule | 17 | 0.358 | 4 | 1.067 | 0.0399 |
| GO:19953: sexual reproduction | 84 | 1.694 | 11 | 3.03 | 0.0412 |
| GO:50876: reproductive physiological process | 84 | 1.694 | 11 | 3.03 | 0.0412 |
| GO:48610: reproductive cellular physiological process | 84 | 1.694 | 11 | 3.03 | 0.0412 |
| GO:746: conjugation | 84 | 1.694 | 11 | 3.03 | 0.0412 |
| GO:7019: microtubule depolymerization | 11 | 0.222 | 3 | 0.826 | 0.0413 |
| GO:8655: pyrimidine salvage | 11 | 0.222 | 3 | 0.826 | 0.0413 |
| GO:31109: microtubule polymerization or depolymerization | 11 | 0.222 | 3 | 0.826 | 0.0413 |
| GO:6800: oxygen and reactive oxygen species metabolism | 74 | 1.493 | 10 | 2.755 | 0.0418 |
| GO:6979: response to oxidative stress | 74 | 1.493 | 10 | 2.755 | 0.0418 |
| GO:278: mitotic cell cycle | 262 | 5.284 | 27 | 7.438 | 0.0421 |
| GO:45005: maintenance of fidelity during DNA-dependent DNA replication | 27 | 0.545 | 5 | 1.377 | 0.0434 |
| GO:6298: mismatch repair | 27 | 0.545 | 5 | 1.377 | 0.0434 |
| GO:16866: intramolecular transferase activity | 18 | 0.395 | 4 | 1.14 | 0.0448 |
| GO:15749: monosaccharide transport | 55 | 1.109 | 8 | 2.204 | 0.045 |
| GO:8645: hexose transport | 55 | 1.109 | 8 | 2.204 | 0.045 |
| GO:752: agglutination during conjugation with cellular fusion | 5 | 0.101 | 2 | 0.551 | 0.0461 |
| GO:83: G1/S-specific transcription in mitotic cell cycle | 5 | 0.101 | 2 | 0.551 | 0.0461 |
| GO:46618: drug export | 5 | 0.101 | 2 | 0.551 | 0.0461 |
| GO:771: agglutination | 5 | 0.101 | 2 | 0.551 | 0.0461 |
| GO:16853: isomerase activity | 72 | 1.581 | 10 | 2.849 | 0.0478 |
| GO:8094: DNA-dependent ATPase activity | 44 | 0.966 | 7 | 1.994 | 0.049 |
| GO:6302: double-strand break repair | 28 | 0.565 | 5 | 1.377 | 0.0497 |
| GO:40020: regulation of meiosis | 28 | 0.565 | 5 | 1.377 | 0.0497 |
| GO:5880: nuclear microtubule | 11 | 0.232 | 3 | 0.8 | 0.05 |
| GO:5576: extracellular region | 71 | 1.494 | 10 | 2.667 | 0.0504 |
| GO:150: recombinase activity | 5 | 0.11 | 2 | 0.57 | 0.0507 |
| GO:4662: CAAX-protein geranylgeranyltransferase activity | 5 | 0.11 | 2 | 0.57 | 0.0507 |
| GO:1727: lipid kinase activity | 5 | 0.11 | 2 | 0.57 | 0.0507 |
| GO:217: DNA secondary structure binding | 5 | 0.11 | 2 | 0.57 | 0.0507 |
| GO:3680: AT DNA binding | 5 | 0.11 | 2 | 0.57 | 0.0507 |
| GO:46527: glucosyltransferase activity | 27 | 0.593 | 5 | 1.425 | 0.0522 |
| GO:3682: chromatin binding | 27 | 0.593 | 5 | 1.425 | 0.0522 |
| GO:15077: monovalent inorganic cation transporter activity | 19 | 0.417 | 4 | 1.14 | 0.0534 |
| GO:15078: hydrogen ion transporter activity | 19 | 0.417 | 4 | 1.14 | 0.0534 |
| GO:15718: monocarboxylic acid transport | 20 | 0.403 | 4 | 1.102 | 0.0537 |
| GO:45229: external encapsulating structure organization and biogenesis | 176 | 3.55 | 19 | 5.234 | 0.0548 |
| GO:7047: cell wall organization and biogenesis | 176 | 3.55 | 19 | 5.234 | 0.0548 |

Table S3 Downregulated genes classified based on annotation and sorted according to significance

| Category | Genes in Category | % of Genes in Category | Genes in List in Category | % of Genes in List in Category | p-Value |
| --- | --- | --- | --- | --- | --- |
| GO:5732: small nucleolar ribonucleoprotein complex | 54 | 1.137 | 17 | 6.719 | 1.05E-09 |
| GO:5730: nucleolus | 217 | 4.567 | 34 | 13.44 | 6.30E-09 |
| GO:30515: snoRNA binding | 55 | 1.208 | 15 | 5.119 | 1.07E-06 |
| GO:9451: RNA modification | 58 | 1.17 | 15 | 4.95 | 1.19E-06 |
| GO:31224: intrinsic to membrane | 384 | 8.083 | 42 | 16.6 | 3.52E-06 |
| GO:4067: asparaginase activity | 4 | 0.0879 | 4 | 1.365 | 1.68E-05 |
| GO:16021: integral to membrane | 368 | 7.746 | 39 | 15.42 | 1.76E-05 |
| GO:17150: tRNA dihydrouridine synthase activity | 7 | 0.154 | 5 | 1.706 | 2.01E-05 |
| GO:44010: single-species biofilm formation | 17 | 0.343 | 7 | 2.31 | 3.39E-05 |
| GO:154: rRNA modification | 26 | 0.524 | 8 | 2.64 | 0.000105 |
| GO:41: transition metal ion transport | 57 | 1.15 | 12 | 3.96 | 0.000128 |
| GO:16072: rRNA metabolism | 85 | 1.714 | 15 | 4.95 | 0.000159 |
| GO:15891: siderophore transport | 3 | 0.0605 | 3 | 0.99 | 0.000226 |
| GO:42928: ferrichrome transport | 3 | 0.0605 | 3 | 0.99 | 0.000226 |
| GO:16338: calcium-independent cell-cell adhesion | 3 | 0.0605 | 3 | 0.99 | 0.000226 |
| GO:51040: regulation of calcium-independent cell-cell adhesion | 3 | 0.0605 | 3 | 0.99 | 0.000226 |
| GO:51042: negative regulation of calcium-independent cell-cell adhesion | 3 | 0.0605 | 3 | 0.99 | 0.000226 |
| GO:30155: regulation of cell adhesion | 3 | 0.0605 | 3 | 0.99 | 0.000226 |
| GO:7162: negative regulation of cell adhesion | 3 | 0.0605 | 3 | 0.99 | 0.000226 |
| GO:5886: plasma membrane | 456 | 9.598 | 42 | 16.6 | 0.000226 |
| GO:4673: protein histidine kinase activity | 6 | 0.132 | 4 | 1.365 | 0.000227 |
| GO:16775: phosphotransferase activity, nitrogenous group as acceptor | 6 | 0.132 | 4 | 1.365 | 0.000227 |
| GO:44011: single-species biofilm formation on inanimate substrate | 16 | 0.323 | 6 | 1.98 | 0.000235 |
| GO:4727: prenylated protein tyrosine phosphatase activity | 3 | 0.0659 | 3 | 1.024 | 0.000264 |
| GO:42927: siderophore transporter activity | 3 | 0.0659 | 3 | 1.024 | 0.000264 |
| GO:42929: ferrichrome transporter activity | 3 | 0.0659 | 3 | 1.024 | 0.000264 |
| GO:31226: intrinsic to plasma membrane | 103 | 2.168 | 15 | 5.929 | 0.000309 |
| GO:42710: biofilm formation | 32 | 0.645 | 8 | 2.64 | 0.000512 |
| GO:6826: iron ion transport | 25 | 0.504 | 7 | 2.31 | 0.000548 |
| GO:33: alpha-1,3-mannosyltransferase activity | 12 | 0.264 | 5 | 1.706 | 0.00058 |
| GO:5665: DNA-directed RNA polymerase II, core complex | 21 | 0.442 | 6 | 2.372 | 0.000592 |
| GO:6493: protein amino acid O-linked glycosylation | 33 | 0.666 | 8 | 2.64 | 0.000641 |
| GO:7234: osmosensory signaling pathway via two-component system | 19 | 0.383 | 6 | 1.98 | 0.000682 |
| GO:15674: di-, tri-valent inorganic cation transport | 78 | 1.573 | 13 | 4.29 | 0.000766 |
| GO:9277: cell wall (sensu Fungi) | 101 | 2.126 | 14 | 5.534 | 0.000815 |
| GO:16720: delta12-fatty acid dehydrogenase activity | 4 | 0.0879 | 3 | 1.024 | 0.00101 |
| GO:5415: nucleoside:sodium symporter activity | 4 | 0.0879 | 3 | 1.024 | 0.00101 |
| GO:15932: nucleobase, nucleoside, nucleotide and nucleic acid transporter activity | 4 | 0.0879 | 3 | 1.024 | 0.00101 |
| GO:5337: nucleoside transporter activity | 4 | 0.0879 | 3 | 1.024 | 0.00101 |
| GO:5887: integral to plasma membrane | 94 | 1.979 | 13 | 5.138 | 0.00127 |
| GO:6829: zinc ion transport | 9 | 0.182 | 4 | 1.32 | 0.00135 |
| GO:15914: phospholipid transport | 9 | 0.182 | 4 | 1.32 | 0.00135 |
| GO:124: SAGA complex | 33 | 0.695 | 7 | 2.767 | 0.00145 |
| GO:160: two-component signal transduction system (phosphorelay) | 22 | 0.444 | 6 | 1.98 | 0.0016 |
| GO:16722: oxidoreductase activity, oxidizing metal ions | 44 | 0.966 | 9 | 3.072 | 0.0016 |
| GO:5034: osmosensor activity | 9 | 0.198 | 4 | 1.365 | 0.00163 |
| GO:16705: oxidoreductase activity, acting on paired donors, with incorporation or reduction of molecular oxygen | 29 | 0.637 | 7 | 2.389 | 0.00194 |
| GO:30001: metal ion transport | 87 | 1.755 | 13 | 4.29 | 0.00216 |
| GO:43412: biopolymer modification | 650 | 13.11 | 57 | 18.81 | 0.00236 |
| GO:30446: hyphal cell wall | 56 | 1.179 | 9 | 3.557 | 0.0025 |
| GO:7231: osmosensory signaling pathway | 24 | 0.484 | 6 | 1.98 | 0.00261 |
| GO:6400: tRNA modification | 32 | 0.645 | 7 | 2.31 | 0.00265 |
| GO:9986: cell surface | 261 | 5.494 | 25 | 9.881 | 0.00273 |
| GO:30312: external encapsulating structure | 130 | 2.736 | 15 | 5.929 | 0.00351 |
| GO:5618: cell wall | 130 | 2.736 | 15 | 5.929 | 0.00351 |
| GO:15743: malate transport | 2 | 0.0403 | 2 | 0.66 | 0.00372 |
| GO:15858: nucleoside transport | 6 | 0.121 | 3 | 0.99 | 0.00394 |
| GO:8785: alkyl hydroperoxide reductase activity | 2 | 0.0439 | 2 | 0.683 | 0.00413 |
| GO:4851: uroporphyrin-III C-methyltransferase activity | 2 | 0.0439 | 2 | 0.683 | 0.00413 |
| GO:3951: NAD+ kinase activity | 2 | 0.0439 | 2 | 0.683 | 0.00413 |
| GO:4743: pyruvate kinase activity | 2 | 0.0439 | 2 | 0.683 | 0.00413 |
| GO:9049: aspartic-type signal peptidase activity | 2 | 0.0439 | 2 | 0.683 | 0.00413 |
| GO:4806: triacylglycerol lipase activity | 2 | 0.0439 | 2 | 0.683 | 0.00413 |
| GO:43136: glycerol-3-phosphatase activity | 2 | 0.0439 | 2 | 0.683 | 0.00413 |
| GO:3876: AMP deaminase activity | 2 | 0.0439 | 2 | 0.683 | 0.00413 |
| GO:42562: hormone binding | 2 | 0.0439 | 2 | 0.683 | 0.00413 |
| GO:15293: symporter activity | 18 | 0.395 | 5 | 1.706 | 0.00456 |
| GO:15294: solute:cation symporter activity | 18 | 0.395 | 5 | 1.706 | 0.00456 |
| GO:5506: iron ion binding | 6 | 0.132 | 3 | 1.024 | 0.00456 |
| GO:18377: protein myristoylation | 12 | 0.242 | 4 | 1.32 | 0.00457 |
| GO:18319: protein amino acid myristoylation | 12 | 0.242 | 4 | 1.32 | 0.00457 |
| GO:6499: N-terminal protein myristoylation | 12 | 0.242 | 4 | 1.32 | 0.00457 |
| GO:4725: protein tyrosine phosphatase activity | 12 | 0.264 | 4 | 1.365 | 0.0055 |
| GO:30532: small nuclear ribonucleoprotein complex | 75 | 1.579 | 10 | 3.953 | 0.00594 |
| GO:15370: solute:sodium symporter activity | 7 | 0.154 | 3 | 1.024 | 0.0076 |
| GO:30529: ribonucleoprotein complex | 328 | 6.904 | 28 | 11.07 | 0.0079 |
| GO:3723: RNA binding | 211 | 4.634 | 23 | 7.85 | 0.00819 |
| GO:8610: lipid biosynthesis | 14 | 0.282 | 4 | 1.32 | 0.00839 |
| GO:5215: transporter activity | 350 | 7.687 | 34 | 11.6 | 0.00888 |
| GO:123: histone acetyltransferase complex | 45 | 0.947 | 7 | 2.767 | 0.00891 |
| GO:16337: cell-cell adhesion | 22 | 0.444 | 5 | 1.65 | 0.00917 |
| GO:18063: cytochrome c-heme linkage | 3 | 0.0605 | 2 | 0.66 | 0.0107 |
| GO:17196: N-terminal peptidyl-methionine acetylation | 3 | 0.0605 | 2 | 0.66 | 0.0107 |
| GO:17006: protein-tetrapyrrole linkage | 3 | 0.0605 | 2 | 0.66 | 0.0107 |
| GO:17003: protein-heme linkage | 3 | 0.0605 | 2 | 0.66 | 0.0107 |
| GO:18206: peptidyl-methionine modification | 3 | 0.0605 | 2 | 0.66 | 0.0107 |
| GO:6279: premeiotic DNA synthesis | 3 | 0.0605 | 2 | 0.66 | 0.0107 |
| GO:6824: cobalt ion transport | 3 | 0.0605 | 2 | 0.66 | 0.0107 |
| GO:293: ferric-chelate reductase activity | 30 | 0.659 | 6 | 2.048 | 0.0108 |
| GO:9013: succinate-semialdehyde dehydrogenase [NAD(P)+] activity | 3 | 0.0659 | 2 | 0.683 | 0.0119 |
| GO:4114: 3',5'-cyclic-nucleotide phosphodiesterase activity | 3 | 0.0659 | 2 | 0.683 | 0.0119 |
| GO:47555: 3',5'-cyclic-GMP phosphodiesterase activity | 3 | 0.0659 | 2 | 0.683 | 0.0119 |
| GO:4638: phosphoribosylaminoimidazole carboxylase activity | 3 | 0.0659 | 2 | 0.683 | 0.0119 |
| GO:8526: phosphatidylinositol transporter activity | 3 | 0.0659 | 2 | 0.683 | 0.0119 |
| GO:6811: ion transport | 144 | 2.904 | 16 | 5.281 | 0.0139 |
| GO:4721: phosphoprotein phosphatase activity | 71 | 1.559 | 10 | 3.413 | 0.0145 |
| GO:44419: interaction between organisms | 286 | 5.768 | 27 | 8.911 | 0.0146 |
| GO:44403: symbiosis, mutualism through parasitism | 286 | 5.768 | 27 | 8.911 | 0.0146 |
| GO:44404: symbiotic interaction between host and other organism | 286 | 5.768 | 27 | 8.911 | 0.0146 |
| GO:5697: telomerase holoenzyme complex | 4 | 0.0842 | 2 | 0.791 | 0.0158 |
| GO:6812: cation transport | 110 | 2.219 | 13 | 4.29 | 0.0159 |
| GO:4888: transmembrane receptor activity | 16 | 0.351 | 4 | 1.365 | 0.0165 |
| GO:16724: oxidoreductase activity, oxidizing metal ions, oxygen as acceptor | 9 | 0.198 | 3 | 1.024 | 0.0166 |
| GO:4322: ferroxidase activity | 9 | 0.198 | 3 | 1.024 | 0.0166 |
| GO:46906: tetrapyrrole binding | 9 | 0.198 | 3 | 1.024 | 0.0166 |
| GO:20037: heme binding | 9 | 0.198 | 3 | 1.024 | 0.0166 |
| GO:5742: mitochondrial outer membrane translocase complex | 11 | 0.232 | 3 | 1.186 | 0.0179 |
| GO:9405: pathogenesis | 251 | 5.063 | 24 | 7.921 | 0.018 |
| GO:2: mitochondrial genome maintenance | 26 | 0.524 | 5 | 1.65 | 0.0188 |
| GO:15290: electrochemical potential-driven transporter activity | 25 | 0.549 | 5 | 1.706 | 0.0195 |
| GO:15291: porter activity | 25 | 0.549 | 5 | 1.706 | 0.0195 |
| GO:16538: cyclin-dependent protein kinase regulator activity | 25 | 0.549 | 5 | 1.706 | 0.0195 |
| GO:6643: membrane lipid metabolism | 10 | 0.202 | 3 | 0.99 | 0.0197 |
| GO:6644: phospholipid metabolism | 10 | 0.202 | 3 | 0.99 | 0.0197 |
| GO:6656: phosphatidylcholine biosynthesis | 4 | 0.0807 | 2 | 0.66 | 0.0206 |
| GO:6657: CDP-choline pathway | 4 | 0.0807 | 2 | 0.66 | 0.0206 |
| GO:46470: phosphatidylcholine metabolism | 4 | 0.0807 | 2 | 0.66 | 0.0206 |
| GO:6831: low-affinity zinc ion transport | 4 | 0.0807 | 2 | 0.66 | 0.0206 |
| GO:723: telomere maintenance | 4 | 0.0807 | 2 | 0.66 | 0.0206 |
| GO:16233: telomere capping | 4 | 0.0807 | 2 | 0.66 | 0.0206 |
| GO:173: inactivation of MAPK activity during osmolarity sensing | 4 | 0.0807 | 2 | 0.66 | 0.0206 |
| GO:188: inactivation of MAPK activity | 4 | 0.0807 | 2 | 0.66 | 0.0206 |
| GO:43086: negative regulation of enzyme activity | 4 | 0.0807 | 2 | 0.66 | 0.0206 |
| GO:51348: negative regulation of transferase activity | 4 | 0.0807 | 2 | 0.66 | 0.0206 |
| GO:6469: negative regulation of protein kinase activity | 4 | 0.0807 | 2 | 0.66 | 0.0206 |
| GO:43407: negative regulation of MAPK activity | 4 | 0.0807 | 2 | 0.66 | 0.0206 |
| GO:42773: ATP synthesis coupled electron transport | 18 | 0.363 | 4 | 1.32 | 0.0212 |
| GO:42775: ATP synthesis coupled electron transport (sensu Eukaryota) | 18 | 0.363 | 4 | 1.32 | 0.0212 |
| GO:16723: oxidoreductase activity, oxidizing metal ions, NAD or NADP as acceptor | 35 | 0.769 | 6 | 2.048 | 0.0225 |
| GO:4559: alpha-mannosidase activity | 4 | 0.0879 | 2 | 0.683 | 0.0227 |
| GO:4737: pyruvate decarboxylase activity | 4 | 0.0879 | 2 | 0.683 | 0.0227 |
| GO:3978: UDP-glucose 4-epimerase activity | 4 | 0.0879 | 2 | 0.683 | 0.0227 |
| GO:4831: tyrosine-tRNA ligase activity | 4 | 0.0879 | 2 | 0.683 | 0.0227 |
| GO:7155: cell adhesion | 80 | 1.614 | 10 | 3.3 | 0.0228 |
| GO:26: alpha-1,2-mannosyltransferase activity | 26 | 0.571 | 5 | 1.706 | 0.0229 |
| GO:30880: RNA polymerase complex | 54 | 1.137 | 7 | 2.767 | 0.0232 |
| GO:1402: signal transduction during filamentous growth | 11 | 0.222 | 3 | 0.99 | 0.0258 |
| GO:6491: N-glycan processing | 20 | 0.403 | 4 | 1.32 | 0.0304 |
| GO:3899: DNA-directed RNA polymerase activity | 58 | 1.274 | 8 | 2.73 | 0.0308 |
| GO:6450: regulation of translational fidelity | 5 | 0.101 | 2 | 0.66 | 0.0329 |
| GO:17004: cytochrome complex assembly | 5 | 0.101 | 2 | 0.66 | 0.0329 |
| GO:6390: transcription from mitochondrial promoter | 5 | 0.101 | 2 | 0.66 | 0.0329 |
| GO:30445: yeast-form cell wall | 58 | 1.221 | 7 | 2.767 | 0.0329 |
| GO:4089: carbonate dehydratase activity | 5 | 0.11 | 2 | 0.683 | 0.0362 |
| GO:16854: racemase and epimerase activity | 5 | 0.11 | 2 | 0.683 | 0.0362 |
| GO:16857: racemase and epimerase activity, acting on carbohydrates and derivatives | 5 | 0.11 | 2 | 0.683 | 0.0362 |
| GO:16020: membrane | 1189 | 25.03 | 76 | 30.04 | 0.0364 |
| GO:15645: fatty-acid ligase activity | 12 | 0.264 | 3 | 1.024 | 0.0376 |
| GO:4467: long-chain-fatty-acid-CoA ligase activity | 12 | 0.264 | 3 | 1.024 | 0.0376 |
| GO:16070: RNA metabolism | 381 | 7.685 | 32 | 10.56 | 0.038 |
| GO:7530: sex determination | 13 | 0.262 | 3 | 0.99 | 0.0409 |
| GO:7531: mating type determination | 13 | 0.262 | 3 | 0.99 | 0.0409 |
| GO:7533: mating type switching | 13 | 0.262 | 3 | 0.99 | 0.0409 |
| GO:7534: gene conversion at mating-type locus | 13 | 0.262 | 3 | 0.99 | 0.0409 |
| GO:77: DNA damage checkpoint | 13 | 0.262 | 3 | 0.99 | 0.0409 |
| GO:16579: protein deubiquitination | 22 | 0.444 | 4 | 1.32 | 0.0418 |
| GO:6869: lipid transport | 22 | 0.444 | 4 | 1.32 | 0.0418 |
| GO:16491: oxidoreductase activity | 420 | 9.225 | 36 | 12.29 | 0.0424 |
| GO:42254: ribosome biogenesis and assembly | 153 | 3.086 | 15 | 4.95 | 0.0456 |
| GO:7028: cytoplasm organization and biogenesis | 153 | 3.086 | 15 | 4.95 | 0.0456 |
| GO:16591: DNA-directed RNA polymerase II, holoenzyme | 90 | 1.894 | 9 | 3.557 | 0.0487 |
| GO:148: 1,3-beta-glucan synthase complex | 7 | 0.147 | 2 | 0.791 | 0.0497 |
| GO:159: protein phosphatase type 2A complex | 7 | 0.147 | 2 | 0.791 | 0.0497 |
| GO:3724: RNA helicase activity | 64 | 1.406 | 8 | 2.73 | 0.0511 |
