# Supplementary material for: UDP-glucose 4, 6-dehydratase Activity Plays an Important Role in Maintaining Cell Wall Integrity and Virulence of Candida albicans
Source: PLoS Pathog. 2011 Nov 17;7(11):e1002384. doi: 10.1371/journal.ppat.1002384 (PMC3219719; doi:10.1371/journal.ppat.1002384)
Supplement: Text S2 — The Microarray data was submitted to the GEO database. The relevant information on the experimental conditions are supplied as the GEO submission file information. (DOC) [file ppat.1002384.s008.doc]

| Status |  |
| --- | --- |
| Title | Gene expression in wild type and Ca*gal102* knockout Candida strains |
| Organism(s) | [*Candida*](http://www.ncbi.nlm.nih.gov/Taxonomy/Browser/wwwtax.cgi?mode=Info&id=10090) *albicans* |
| Experiment type | Expression profiling by array |
| Summary | Ca*GAL102* is a sequence homolog of Rmlb. In Candida knock out of this gene causes abnormal hyphal morphogenesis and increased sensitivity to cell wall damaging agents. The knock out strain is also avirulent in mouse model of systemic infection. To get a larger insight into the function of the protein product of this gene we carried out global transcription analysis through micro array experiment. The gene is expressed under normal growth conditions and the knock out causes the cells to become hyphal under these conditions. Many of the cell wall proteins were upregulated recapitulating the cell morphology.  Keywords: Candida albicans, Gene knockout, genome wide transcription profiling study |
|  |  |
| Overall design | Wild type Sc5314 cells and the mutant CAS12 were grown in YPD till (OD 600nm) 1 following which total RNA was extracted using hot phenol method. The RNA was checked using Bioanalyser (Agilent). Single dye experiment was carried out using Cy3 labelled pooled wild type samples and two independent mutant RNA samples. |
