# Supplementary material for: UDP-glucose 4, 6-dehydratase Activity Plays an Important Role in Maintaining Cell Wall Integrity and Virulence of Candida albicans
Source: PLoS Pathog. 2011 Nov 17;7(11):e1002384. doi: 10.1371/journal.ppat.1002384 (PMC3219719; doi:10.1371/journal.ppat.1002384)
Supplement: Text S3 — Supplemental materials and methods. (DOC) [file ppat.1002384.s009.doc]

**Text S3**

**Supplemental Materials and Methods:**

**Disruption of *GAL102*:** Insertional inactivation of the open reading frames in both the alleles was achieved by PCR based gene targeting method as described previously [52]. Briefly, the pFA-URA3 and the pFA-HIS1 cassettes were PCR amplified using the Ca put. GAL10_del_F and Ca put GAL10_del_R primers (Table 3) and transformed in BWP17 using the PEG-Lithium acetate method. After the first round of transformation, transformants were plated on histidine minus plates and the genomic DNA of 10 independent transformants and BWP17 were extracted. The first allele deletion was confirmed by PCR, thus generating the strain *GAL102*/ *GAL102*::*HIS1.* In the second round of transformation, the transformants were plated on uridine and histidine minus plates and the second allele deletion was confirmed by PCR from genomic DNA thus generating the strain *GAL102*::*HIS1/ GAL102::URA3* (URA3+ HIS1+ arg4-). The *gal102Δ/Δ* strain thus obtained was subsequently transformed with pFA-ARG4-MET3 plasmid linearised by XbaI [52] thus generating an *gal102Δ/Δ* strain prototrophic for all the three markers. Genomic DNA was extracted from BWP17, *GAL102*/ *GAL102::HIS1*, *GAL102*:*:HIS1*/ *GAL102*:*:URA3* and was subjected to Southern Blotting analysis to confirm the correct integration at the desired locus.

**Southern and Northern hybridization**: The probe used for southern and northern blots was made as follows. The C- terminal part of the open reading frame was amplified using Taq DNA polymerase (Finnzymes) using the Ca Put Gal10 int (f) and the Ca Put Gal10 (r) primers (Table 3). The PCR product was cleaned using PCR cleanup Kit (Qiagen) and labeled using random hexamers and Klenow Polymerase (MBI) with 30µCi of (α-P32)-dATP at 37°C for 1 hr. The reaction was terminated by heat inactivation at 95°C for 10 min. The probe was purified using a 1ml sepharose G-50 column (Sigma). After determining incorporation, the probe was used at 1x 10^6^ cpm per ml of hybridization buffer.

5µg of total RNA was run on a 1% agarose-formaldehyde gel to check the quality. For RT, 10μg of total RNA was subjected to DNaseI (Roche) treatment at 42˚C for 2hrs followed by heat denaturation of the enzyme at 90˚C for 10 min. Following the DNaseI treatment, 5μg of RNA was reverse transcribed using M-Mulv RT (MBI ) using oligo dT primer at 42°C for 90 min following which PCR was done with gene specific primers for Ca*GAL10* and Ca put*GAL10* (Table 3). The mRNA levels were normalized against ACT1 which was amplified using ACT1 (f) and ACT1 (r) primers (Table 3).
